# Amide conjugates of the jasmonate precursor *cis*-(+)-12-oxo-phytodienoic acid regulate its homeostasis during plant stress responses

**DOI:** 10.1093/plphys/kiae636

**Published:** 2024-11-28

**Authors:** Jitka Široká, Anita Ament, Václav Mik, Tomáš Pospíšil, Michaela Kralová, Chao Zhang, Markéta Pernisová, Michal Karady, Vladimira Nožková, Yuho Nishizato, Takuya Kaji, Rina Saito, Mohamed Htitich, Kristýna Floková, Claus Wasternack, Miroslav Strnad, Minoru Ueda, Ondřej Novák, Federica Brunoni

**Affiliations:** Laboratory of Growth Regulators, Faculty of Science, Palacký University, Šlechtitelů 27, Olomouc CZ-77900, Czech Republic; Laboratory of Growth Regulators, Institute of Experimental Botany, The Czech Academy of Sciences, Šlechtitelů 27, Olomouc CZ-77900, Czech Republic; Laboratory of Growth Regulators, Faculty of Science, Palacký University, Šlechtitelů 27, Olomouc CZ-77900, Czech Republic; Laboratory of Growth Regulators, Institute of Experimental Botany, The Czech Academy of Sciences, Šlechtitelů 27, Olomouc CZ-77900, Czech Republic; Department of Experimental Biology,Faculty of Science, Palacký University, Šlechtitelů 27, Olomouc CZ-77900, Czech Republic; Laboratory of Growth Regulators, Faculty of Science, Palacký University, Šlechtitelů 27, Olomouc CZ-77900, Czech Republic; Laboratory of Growth Regulators, Institute of Experimental Botany, The Czech Academy of Sciences, Šlechtitelů 27, Olomouc CZ-77900, Czech Republic; Department of Chemical Biology, Faculty of Science, Palacký University, Šlechtitelů 27, CZ-77900 Olomouc, Czech Republic; Laboratory of Growth Regulators, Faculty of Science, Palacký University, Šlechtitelů 27, Olomouc CZ-77900, Czech Republic; Laboratory of Growth Regulators, Institute of Experimental Botany, The Czech Academy of Sciences, Šlechtitelů 27, Olomouc CZ-77900, Czech Republic; Laboratory of Growth Regulators, Faculty of Science, Palacký University, Šlechtitelů 27, Olomouc CZ-77900, Czech Republic; Laboratory of Growth Regulators, Institute of Experimental Botany, The Czech Academy of Sciences, Šlechtitelů 27, Olomouc CZ-77900, Czech Republic; Laboratory of Functional Genomics and Proteomics, National Centre for Biomolecular Research, Faculty of Science & Plant Sciences Core Facility, Mendel Centre for Plant Genomics and Proteomics, CEITEC, Masaryk University, Kamenice 5, CZ-62500 Brno, Czech Republic; Laboratory of Growth Regulators, Faculty of Science, Palacký University, Šlechtitelů 27, Olomouc CZ-77900, Czech Republic; Laboratory of Growth Regulators, Institute of Experimental Botany, The Czech Academy of Sciences, Šlechtitelů 27, Olomouc CZ-77900, Czech Republic; Department of Chemical Biology, Faculty of Science, Palacký University, Šlechtitelů 27, CZ-77900 Olomouc, Czech Republic; Graduate School of Science, Tohoku University, Aoba-ku, Sendai JP-980-8578, Japan; Graduate School of Science, Tohoku University, Aoba-ku, Sendai JP-980-8578, Japan; Graduate School of Science, Tohoku University, Aoba-ku, Sendai JP-980-8578, Japan; Department of Development and Environmental Studies, Faculty of Science, Palacký University, tř. 17. listopadu 12, CZ-77146 Olomouc, Czech Republic; Laboratory of Growth Regulators, Faculty of Science, Palacký University, Šlechtitelů 27, Olomouc CZ-77900, Czech Republic; Laboratory of Growth Regulators, Institute of Experimental Botany, The Czech Academy of Sciences, Šlechtitelů 27, Olomouc CZ-77900, Czech Republic; Department of Molecular Signal Processing, Leibniz Institute of Plant Biochemistry, Weinberg 3, Halle (Saale) D-06120, Germany; Laboratory of Growth Regulators, Faculty of Science, Palacký University, Šlechtitelů 27, Olomouc CZ-77900, Czech Republic; Laboratory of Growth Regulators, Institute of Experimental Botany, The Czech Academy of Sciences, Šlechtitelů 27, Olomouc CZ-77900, Czech Republic; Graduate School of Science, Tohoku University, Aoba-ku, Sendai JP-980-8578, Japan; Laboratory of Growth Regulators, Faculty of Science, Palacký University, Šlechtitelů 27, Olomouc CZ-77900, Czech Republic; Laboratory of Growth Regulators, Institute of Experimental Botany, The Czech Academy of Sciences, Šlechtitelů 27, Olomouc CZ-77900, Czech Republic; Laboratory of Growth Regulators, Faculty of Science, Palacký University, Šlechtitelů 27, Olomouc CZ-77900, Czech Republic; Laboratory of Growth Regulators, Institute of Experimental Botany, The Czech Academy of Sciences, Šlechtitelů 27, Olomouc CZ-77900, Czech Republic

## Abstract

Jasmonates are a family of oxylipin phytohormones regulating plant development and growth and mediating “defense versus growth” responses. The upstream JA biosynthetic precursor *cis*-(+)-12-oxo-phytodienoic acid (*cis*-OPDA) acts independently of CORONATIVE INSENSITIVE 1-mediated JA signaling in several stress-induced and developmental processes. However, its perception and metabolism are only partially understood. An isoleucine analog of the biologically active JA-Ile, OPDA-Ile, was detected years ago in wounded leaves of flowering plants, opening up the possibility that conjugation of *cis*-OPDA to amino acids might be a relevant mechanism for *cis*-OPDA regulation. Here, we extended the analysis of amino acid conjugates of *cis*-OPDA and identified naturally occurring OPDA-Val, OPDA-Phe, OPDA-Ala, OPDA-Glu, and OPDA-Asp accumulating in response to biotic and abiotic stress in Arabidopsis (*Arabidopsis thaliana*). The OPDA amino acid conjugates displayed *cis*-OPDA-related plant responses in a JA-Ile-dependent manner. We also showed that the synthesis and hydrolysis of *cis*-OPDA amino acid conjugates are mediated by members of the amidosynthetase GRETCHEN HAGEN 3 and the amidohydrolase INDOLE-3-ACETYL-LEUCINE RESISTANT 1/ILR1-like families. Thus, OPDA amino acid conjugates function in the catabolism or temporary storage of *cis*-OPDA in stress responses instead of acting as chemical signals per se.

## Teaser

Amide conjugate synthesis and hydrolysis control the homeostasis of a jasmonate precursor during stress responses in Arabidopsis.

## Introduction

Plant hormones are a structurally unrelated collection of small molecules derived from various essential metabolic pathways. Collectively these compounds control numerous aspects of plant life, from pattern formation during development to responses to biotic and abiotic stress throughout the plant kingdom (Brunoni et al. 2019b; Blázquez et al. 2020). In the pantheon of plant hormones, jasmonates are a family of oxylipin phytohormones produced by oxidative metabolism of polyunsaturated fatty acids, regulating aspects of plant development and growth, such as seed germination, root growth, flowering time, stamen development, and senescence (Wasternack and Hause 2013; Wasternack and Feussner 2018). Jasmonates are also produced markedly upon abiotic and biotic stresses, such as wounding, insect herbivory, and pathogen infection (Wasternack and Feussner 2018). The biosynthesis of jasmonates is a complex process mediated by sequential enzymatic reactions involving 3 subcellular compartments. Initially, the biosynthesis process occurs in the plastid, where oxidation of the unsaturated fatty acid α-linolenic acid (18:3) mediated by the enzyme 13-LIPOXYGENASE (LOX) takes place (Wasternack and Feussner 2018). Subsequent dehydration and cyclization reactions catalyzed by ALLENE OXIDE SYNTHASE (AOS) and ALLENE OXIDE CYCLASE (AOC), respectively, form *cis*-(+)-12-oxo-phytodienoic acid (*cis*-OPDA). A parallel biosynthetic pathway, starting with hexadecatrienoic acid (16:3) and undergoing similar reactions, produces dinor-OPDA (dnOPDA; Weber et al. 1997). *cis*-OPDA and dnOPDA are then imported through the ABC transporter COMATOSE (CTS; Theodoulou et al. 2005) to the peroxisome, where their cyclopentenone ring is reduced to 3-oxo-2-(2-(*Z*)-pentenyl)-cyclopentane-1-octanoic (OPC-8) and hexanoic (OPC-6) acids, respectively, by OPDA REDUCTASE 3 (OPR3), followed by 3 cycles of β-oxidation to generate 3-oxo-2-(2-(*Z*)-pentenyl)-cyclopentane-1-butanoic acid (OPC-4), and finally leading to jasmonic acid (JA) formation (Breithaupt et al. 2006). Alternatively, JA formation can occur by conversion of *cis*-OPDA to dnOPDA, which in turn undergoes peroxisomal β-oxidation, followed by OPR2-mediated cytosolic reduction, thus bypassing the OPR3 pathway (Chini et al. 2018). In all instances, once JA is formed, it is then conjugated in the cytosol with isoleucine to (+)-7-*iso*-jasmonyl-L-isoleucine (JA-Ile) or with other amino acids by the 2 members of the acyl acid amide synthetases belonging to the GRETCHEN HAGEN 3 (GH3) family, JASMONATE RESISTANT1 (JAR1/GH3.11) and GH3.10 (Staswick and Tiryaki 2004; Delfin et al. 2022). Upon stress or developmentally regulated processes, JA-Ile level increases, thus leading to its binding to the jasmonate coreceptor complex formed by the F-box CORONATIVE INSENSITIVE 1 (COI1) and the jasmonate ZIM domain (JAZ) in which JA-Ile acts as “molecular glue” (Fonseca et al. 2009; Yan et al. 2009; Sheard et al. 2010). JA-Ile-mediated COI1–JAZ interaction triggers ubiquitination of JAZ repressors and their degradation by the proteasome, thus activating the transcription factors that regulate the JA-specific physiological responses (Chini et al. 2007; Thines et al. 2007; Monte et al. 2022). JA-Ile is turned over by 2 inducible and intertwined catabolic pathways. One pathway proceeds via deconjugation by the 3 members of the amidohydrolase indole-3-acetic acid (IAA)-LEUCINE RESISTANT 1 (ILR1) and ILR1-like (ILL) family, ILL6 and IAR3 (Widemann et al. 2013; Zhang et al. 2016). The other one is oxidative, mediated by cytochrome P450 enzymes of the subfamily 94 (CYP94), and leads to the formation of 12-OH-JA-Ile and 12-COOH-JA-Ile (Koo et al. 2011, 2014). However, recently, one of the 12-OH-JA-Ile stereoisomers was found to bind to COI1–JAZ9 as effectively as JA-Ile (Saito et al. 2023), functioning as an active jasmonate signal rather than a catabolite in wound and defense responses (Jimenez-Aleman et al. 2019; Poudel et al. 2019). An additional catabolic pathway based on JA oxidation to OH-JA, catalyzed by members of the 2-oxoglutarate/Fe(II) dioxygenase (2OGD) family, regulates JA turnover upstream of the JA-Ile formation (Caarls et al. 2017; Smirnova et al. 2017).

Although *cis*-OPDA functions primarily as a JA precursor, it also possesses a signaling role distinct from JA-Ile. Any interaction of *cis*-OPDA via the COI1–JAZ coreceptor complex was excluded, thus supporting the OPDA perception via an alternative route (namely COI1-independent pathway; Thines et al. 2007; Fonseca et al. 2009; Yan et al. 2009). Remarkably, in contrast to vascular plants, the liverwort *Marchantia polymorpha* and the moss *Physcomitrium patens* produce *cis*-OPDA and dnOPDA, but not JA and JA-Ile, and isomeric forms of dnOPDA were identified as ligands of MpCOI1 (Monte et al. 2018; Kneeshaw et al. 2022; Široká et al. 2022). Several physiological processes, such as tendril coiling, seed germination, thermotolerance, stomatal opening, and response to pathogens, have been attributed to *cis*-OPDA (Weiler et al. 1993; Dave et al. 2011, 2016; Monte et al. 2020; Chang et al. 2023). Moreover, *cis*-OPDA and dnOPDA, but not JA and JA-Ile, contain α, β-unsaturated carbonyl group conferring reactive electrophilic properties (Mueller and Berger 2009; Farmer and Mueller 2013), and their chemical reactivity likely activates the COI1-independent thermotolerance response in streptophytes (Monte et al. 2020).

It has been proposed that *cis*-OPDA exerts its regulatory function by reversibly binding the cyclophilin 20-3 (CYP20-3), thus stabilizing enzymes involved in cysteine synthesis (Park et al. 2013). This event triggers the glutathione (GSH) level increase, thus determining redox changes in the plastid and cytosol and *cis*-OPDA-mediated TGA (basic leucine zipper, bZIP) transcription factor activation (Park et al. 2013; Jimenez-Aleman et al. 2022). Substantial steps have been taken to decipher the biological properties and biosynthesis of *cis*-OPDA. However, the mechanisms by which plants control the levels of *cis*-OPDA still need to be discovered. Very recently, a jasmonate-induced dioxygenase 1 (JID1) belonging to the 2OGD superfamily has been suggested to play an essential role in the JA-mediated regulation of plant defense responses as accumulation of *cis*-OPDA decreased in plants overexpressing JID1 upon wounding (Yi et al. 2023). Nonetheless, JID1 did not directly modify *cis*-OPDA; thus, it might indirectly affect the accumulation of *cis*-OPDA through an unknown pathway (Nishizato et al. 2024). Several conjugates of *cis*-OPDA, such as OPDA–GSH and OPDA amino acid conjugates (namely OPDA-aa), have been identified (Ohkama-Ohtsu et al. 2011; Floková et al. 2016; Shinya et al. 2022). On the one hand, conjugation of *cis*-OPDA with GSH is described as a possible detoxification mechanism of *cis*-OPDA after stress, as this conjugate accumulates in the vacuole, and its reactivity is linked to cellular redox homeostasis (Ohkama-Ohtsu et al. 2011; Park et al. 2013). On the other hand, since conjugation of JA with amino acids is required for its bioactivity and homeostasis, it is tempting to speculate that a similar scenario may occur in the case of *cis*-OPDA-specific response. In Arabidopsis (*Arabidopsis thaliana*), OPDA-Ile was identified as a low abundant metabolite in wounded leaves and proposed to act as a regulatory molecule in a JA-independent manner (Arnold et al. 2016; Floková et al. 2016). OPDA-Ile was shown to induce the expression of genes encoding for the C_2_H_2_-type zinc finger transcription factor, ZAT10, and the glutaredoxin, GRX480, that were previously identified as *cis*-OPDA-inducible (Taki et al. 2005; Park et al. 2013; Arnold et al. 2016). Several OPDA-aa have recently been putatively identified in rice and proposed as noncanonical signaling molecules for producing phytoalexins in coordination with innate chitin signaling (Shinya et al. 2022). Overall, these findings support the concept that plants need multiple metabolic pathways at disposal to modulate *cis*-OPDA action. However, the mechanism by which OPDA-aa are formed and their role and function in plants still need to be discovered and better understood.

Here, we described the accumulation and role of naturally occurring OPDA-Val, OPDA-Phe, OPDA-Ala, OPDA-Glu, and OPDA-Asp. These conjugates built up upon wounding stress, *cis*-OPDA homeostasis perturbation, and fungal pathogen infection in Arabidopsis. Like free *cis*-OPDA, the identified OPDA-aa exhibit a JA-Ile-dependent growth-inhibitory effect, trigger the JAZ1 repressor degradation, and influence the expression of JA- and *cis*-OPDA-responsive genes. Moreover, we showed that members of the GH3 and ILR1/ILL families catalyze the conjugation of *cis*-OPDA to amino acids and hydrolysis of the OPDA-aa, respectively, in response to mechanical wounding. Our data corroborate that several enzymes act redundantly in the metabolism of *cis*-OPDA, and amido synthetases and amidohydrolases specifically contribute to maintaining the balance between active and inactive forms of *cis*-OPDA. Thus, similarly to other phytohormones such as auxins and JA, one mechanism of regulating *cis*-OPDA homeostasis appears to be the synthesis and hydrolysis of amide conjugates, which function in the catabolism or temporary storage of *cis*-OPDA in stress responses.

## Results

### OPDA-aa accumulate in response to biotic and abiotic stress in Arabidopsis

**Figure 1.**
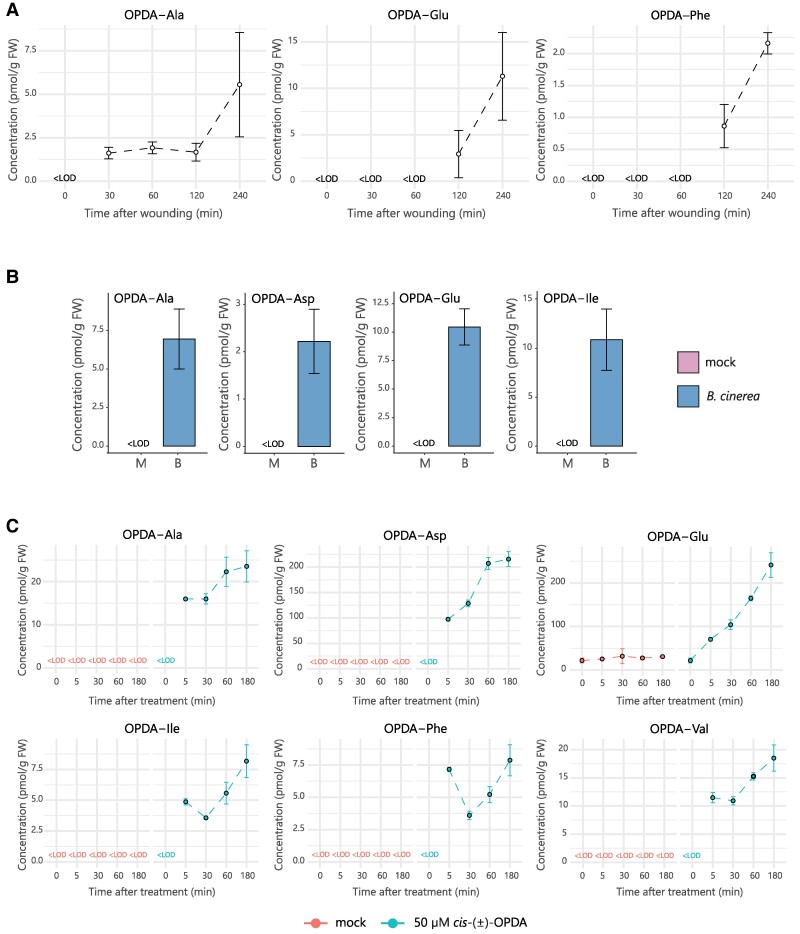
OPDA-aa accumulate in Arabidopsis plants during stress responses. **A)** Time-course accumulation of indicated OPDA-aa in Col-0 after leaf wounding. Six-week-old plants were wounded, and damaged leaves were collected after the indicated times. **B)** Accumulation of indicated OPDA-aa in Col-0 plants infected with *B. cinerea* 4 d after inoculation (B) or mock-inoculated (M). **C)** Time-course accumulation of indicated OPDA-aa after exogenous treatment with 50 µM *cis*-(±)-OPDA. Plants were sampled after the indicated times. Ala, Alanine; Asp, aspartate; Glu, glutamate; Ile, isoleucine; Phe, phenylalanine; Val, valine. OPDA-aa levels are expressed as pmoles per gram of plant FW. Mean ± SD (*n* = 3). Below the limit of detection, <LOD.

We previously demonstrated that OPDA-Asp, OPDA-Glu, OPDA-Ile, OPDA-Phe, and OPDA-Val were detected in Arabidopsis wounded leaves (Mik et al. 2023). To explore whether the occurrence of OPDA-aa is a specific or a broad-spectrum metabolic response to stressful events in Arabidopsis, we monitored the formation of OPDA-aa in several well-studied conditions known to alter JA and *cis*-OPDA homeostasis, including leaf wounding, pathogen infection, and chemical treatment. These OPDA-aa did not accumulate in the earliest time points postwounding but were instead found in the latest time points after injury, suggesting that OPDA-aa formation might follow a kinetics that differs from the rapid JA-Ile wounding response (Koo et al. 2009; Mik et al. 2023). Therefore, we performed an experiment and focused on OPDA-aa occurrence in the time points ranging from 30 min to 4 h after wounding. We found *cis*-OPDA conjugated with Ala, Glu, and Phe, while OPDA-Ile, OPDA-Val, OPDA-Asp, and OPDA-Trp were not detected in wounded wild-type Arabidopsis leaves under our experimental conditions (Fig. 1A). Except for OPDA-Ala, which accumulated 30 min after wounding and kept increasing after 4 h, OPDA-Glu and OPDA-Phe were detected by 2 h and peaked by 4 h postwounding. We also monitored the amino acid conjugation of JA. We observed that, upon wounding, OPDA-aa accumulated with kinetics that was more similar to the less abundant JA-Ala and JA-Val than the typical rapid JA-Ile accumulation (Supplementary Fig. S1A; Koo et al. 2009). OPDA-aa levels ranged between 1 and 10 pmol/g fresh weight (FW), consistent with previous results (Mik et al. 2023). Some differences were also observed (Fig. 1A; Mik et al. 2023). OPDA-Ile, OPDA-Val, and OPDA-Asp were not detected, and OPDA-Ala was found upon wounding in this study, while OPDA-Ile, OPDA-Val, and OPDA-Asp occurred and OPDA-Ala was not identified in the previous study. This could be explained by the different experimental settings adopted for the wounding experiment; first, older plants grown under a neutral photoperiod (12 h light/12 h dark) were used in this study, whereas younger plants grown under long-day photoperiod (16 h light/8 h dark) were used in the previous study. Secondly, mechanical stress was performed on leaves by wounding 3 times the midvein in this study, while leaves were wounded once on one side of the midvein in the previous experiment.

To assess whether the formation of OPDA-aa could be elicited upon fungal attack, we inoculated Arabidopsis plantlets with spores of *Botrytis cinerea*, a broad-spectrum pathogen widely used to study plants response to biotic stress (Chini et al. 2018). We found *cis*-OPDA conjugated to Ala, Asp, Glu, and Ile only in *B. cinerea*-inoculated plants, while none of the OPDA-aa was detected in mock-treated plants (Fig. 1B). OPDA-Phe, OPDA-Val, and OPDA-Trp were not detected in response to this pathogen or mock samples. We also quantified JA-aa and observed high levels of JA-Ala and JA-Glu, and that accumulation of JA-Ile, JA-Asp, JA-Gly, and JA-Val occurred to a lesser extent (Supplementary Fig. S1B). On the contrary, a similar range of OPDA-aa levels was recorded by wounding and fungal infection (Fig. 1A and B).

Compared with the response observed in wounded and infected plants, a wider variety of OPDA-aa was detected after exogenous treatment with *cis*-(±)-OPDA (Fig. 1). All the inspected OPDA-aa, except OPDA-Trp, were rapidly formed upon application of *cis*-(±)-OPDA 5 min after treatment, with OPDA-Asp and OPDA-Glu being the most abundant OPDA-aa (Fig. 1C). The range of accumulated OPDA-aa levels was much less than that of JA-Ile (Fig. 1A; Supplementary Fig. S1A; Koo et al. 2009). These results suggest that *cis*-OPDA amino acid conjugation is a metabolic mechanism which allows plants to adapt and respond to a wide range of physiological conditions perturbing the *cis*-OPDA and JA homeostasis.

### Study of the activity of OPDA-aa in *cis*-OPDA- and JA-regulated responses

**Figure 2.**
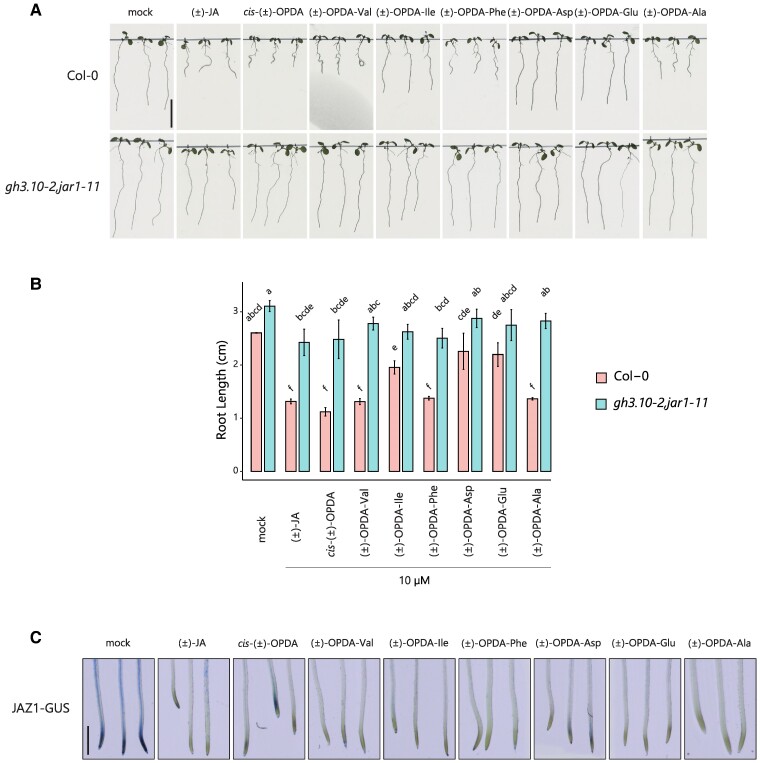
(±)-OPDA-aa exhibit *cis*-OPDA-like activity in a JA-Ile-dependent manner. **A)** Wild-type (Col-0) and *gh3.10-2,jar1-11* mutant seedlings grown on vertical plates in the absence (mock) or presence of 10 µM (±)-JA, *cis*-(±)-OPDA, and indicated (±)-OPDA-aa. Scale bar in the top left inset represents 1 cm and applies to all the insets of the panel. **B)** Root length of 14 to 15 seedlings was measured 7 d after germination. Data are shown as mean ± SD of 3 biological replicates (*n* = 3). Letters indicate significant differences, evaluated by 1-way ANOVA/Tukey HSD post hoc test (*P* < 0.05). **C)** Representative seedlings of the *35S:JAZ1-GUS* line treated with or without 10 µM (±)-JA, *cis*-(±)-OPDA, and indicated (±)-OPDA-aa for 2 h. Scale bar in the first inset represents 1 mm and applies to all the insets of the panel. Ala, Alanine; Asp, aspartate; Glu, glutamate; Ile, isoleucine; Phe, phenylalanine; Val, valine.

JA and *cis*-OPDA inhibit root growth (Monte et al. 2018). Therefore, a primary root-growth inhibition assay was carried out to examine possible *cis*-OPDA-related phenotype upon treatment with (±)-OPDA-aa. Since OPDA-Trp was not detected in any of the experiments described above (Fig. 1), this molecule was excluded in the following experiments. Overall root growth was significantly reduced on (±)-OPDA-aa-containing medium compared with mock-treated seedlings. Nonetheless, we observed that treatment with (±)-OPDA-Val, (±)-OPDA-Phe, and (±)-OPDA-Ala impaired root length similarly to *cis*-(±)-OPDA and significantly more than (±)-OPDA-Ile, (±)-OPDA-Asp, and (±)-OPDA-Glu (Fig. 2A and B). It should be noted that a racemic mixture of (±)-OPDA-aa, made from racemic *cis*-(±)-OPDA, was used when applied exogenously; thus, only 50% of the applied chemicals has the proper stereochemistry and is biologically active. To investigate whether the root-growth-inhibitory effect of these (±)-OPDA-aa required in planta conjugation of JA to Ile, we also treated *gh3.10-2,jar1-11* double mutant seedlings with (±)-OPDA-aa. We chose *gh3.10-2, jar1-11* rather than other JA biosynthetic mutants, such as *opr3-3*, because although both mutant lines are dramatically impaired in the JA-Ile synthesis, *gh3.10-2,jar1-11* is fertile (Chini et al. 2018; Delfin et al. 2022). Not (±)-OPDA-aa nor *cis*-(±)-OPDA and (±)-JA inhibited the root growth of *gh3.10-2,jar1-11* mutant, demonstrating that, likewise to JA and *cis*-OPDA, the (±)-OPDA-aa-induced root-growth-inhibitory effect requires JA-Ile formation (Fig. 2A and B).

**Figure 3.**
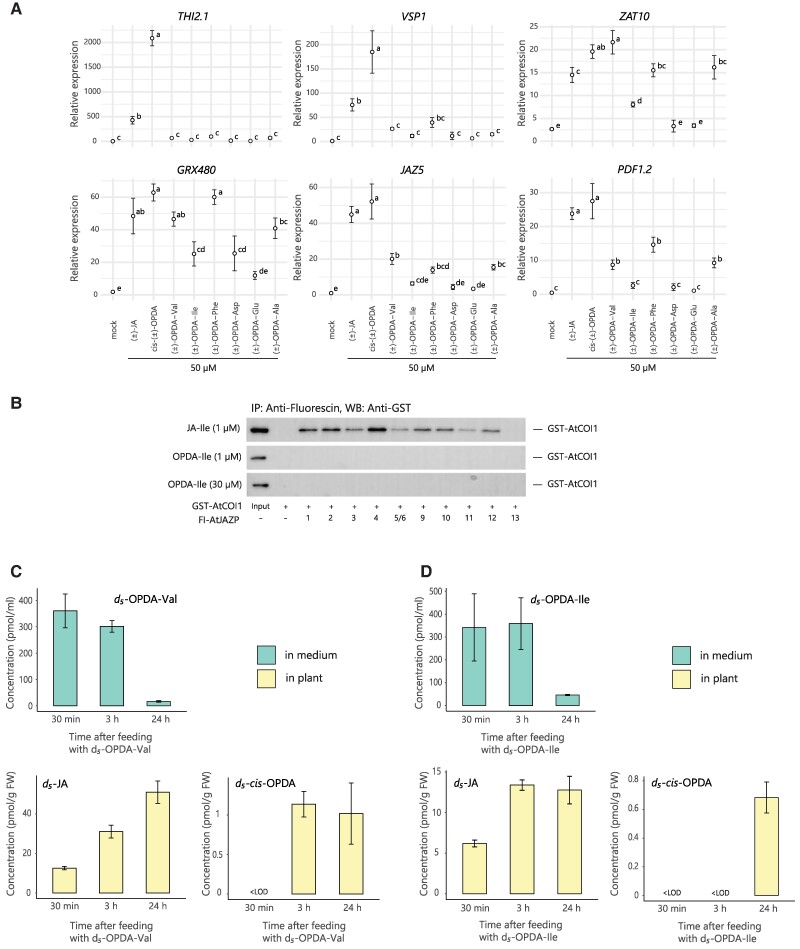
Analyses of gene expression in response to (±)-OPDA-aa, affinity between OPDA-Ile and COI-JAZs, and conversion of OPDA-aa into *cis*-OPDA and JA. **A)** Expression of *THI2.1*, *VSP1*, *ZAT10*, *GRX480*, *JAZ5*, and *PDF1.2* after 3 h-treatment with or without 50 µM (±)-JA, *cis*-(±)-OPDA, and indicated (±)-OPDA-aa in Col-0. Gene expression was measured by RT-qPCR. Data are expressed as relative fold change normalized by *ACT2*. Letters indicate significant differences, evaluated by 1-way ANOVA/Tukey HSD post hoc test (*P* < 0.05). **B)** Pull-down assay of GST-AtCOI1 with Fl-AtJAZPs in the presence of JA-Ile (1 µM) or OPDA-Ile (1 or 30 µM). GST-AtCOI1 bound to Fl-AtJAZPs was pulled-down with an antifluorescein antibody and Protein G magnetic beads and analyzed by immunoblotting (anti-GST-HRP conjugate for detection of GST-AtCOI1) (95 kDa). Fl-AtJAZ13 was used as a negative control because JAZ13 had no canonical JAZ degron sequence, which is necessary for JA-Ile perception. This experiment was repeated 3 times with similar results. **C, D**) Time-course accumulation of stable isotope-labeled derivatives of OPDA-Val and OPDA-Ile in liquid ½ MS medium (pmol/mL) and 7-d-old Col-0 seedlings (pmol/g FW) after feeding with 10 µM *d_5_*-OPDA-Val (**C**) and *d_5_*-OPDA-Ile (**D**). Samples were collected at the indicated times. Ala, Alanine; Asp, aspartate; Glu, glutamate; Ile, isoleucine; Phe, phenylalanine; Val, valine. Mean ± Sd (*n* = 3). Below the limit of detection, <LOD.

To test whether these molecules could impact the JA signaling pathway, we investigated the effect of (±)-OPDA-aa on hormone-induced degradation of JAZ repressors, a typical COI1-mediated response, using the Arabidopsis *35S:JAZ1*-GUS reporter line (Thines et al. 2007). All the (±)-OPDA-aa triggered JAZ1 protein degradation, similarly to (±)-JA and *cis*-(±)-OPDA (Fig. 2C). We further monitored the effect of (±)-OPDA-aa treatment on the expression of proposed *cis*-OPDA-markers, such as *ZAT10* and *GRX480*, JA-markers, such as *JAZ5* and *VEGETATIVE STORAGE PROTEIN1* (*VSP1*), and defense-related genes, such as *PLANT DEFENSIN1.2* (*PDF1.2*) and *thionin* (*THI2.1*; Staswick and Tiryaki 2004; Taki et al. 2005; Arnold et al. 2016; Chini et al. 2018), and compared their expression level upon treatment with (±)-JA, *cis*-(±)-OPDA or mock (Fig. 3A). None of the (±)-OPDA-aa triggered the expression of *THI2.1* and *VSP1*, whereas the transcripts of the other marker genes increased, compared with the mock treatment, although with some differences. On the one hand, (±)-OPDA-Val, (±)-OPDA-Phe, and (±)-OPDA-Ala were found equally effective in upregulating the expression of *ZAT10*, *GRX480*, and *PDF1.2* genes, with the induction of the *ZAT10* and *GRX480* expression comparable to *cis*-(±)-OPDA and (±)-JA. On the other hand, no significant upregulation of *PDF1.2* and *JAZ5* expression levels was observed upon (±)-OPDA-Ile, (±)-OPDA-Asp, and (±)-OPDA-Glu, and in general, the response exerted by this group of (±)-OPDA-aa was consistently lower than the conjugates with Val, Phe, and Ala (Fig. 3A).

A previous study proposed for OPDA-Ile a role as a signal molecule in planta, as negligible cleavage of OPDA-Ile followed by conversion to JA was recorded (Arnold et al. 2016). Besides, a screening of JA precursors and derivatives for binding capacity to the COI1–JAZ coreceptor complex revealed no activity for *cis*-OPDA (Thines et al. 2007; Fonseca et al. 2009). However, the possible interaction of OPDA-Ile with this complex was not tested. Thus, we performed a pull-down experiment with OPDA-Ile using GST-tagged AtCOI1 and fluorescein-tagged AtJAZ1-6/9-13 and did not observe any affinity of OPDA-Ile for the COI1–JAZ coreceptor pairs regardless of the OPDA-Ile tested concentration, confirming that OPDA-Ile is not a ligand for functional COI1–JAZ coreceptors (Fig. 3B).

Our phenotypic results on root growth by (±)-OPDA-aa (Fig. 2A and B) hinted that these conjugates likely exerted their inhibitory function upon hydrolysis of the amino acid moiety followed by conversion to JA-Ile. Hence, to investigate whether plants can hydrolyze OPDA-aa in planta, we treated Col-0 seedlings with deuterium-labeled (*d_5_*) OPDA-Ile and OPDA-Val and monitored *d_5_*-derivatives accumulation over time. Both *d_5_*-OPDA-aa were taken up by Arabidopsis seedlings, as their level dropped in the culture medium within 24 h (Fig. 3C and D). In plants, *d_5_*-OPC4, *d_5_*-JA, and *d_5_*-JA-Ile were detected 30 min after treatment with both *d_5_*-OPDA-aa, while *d_5_*-*cis*-OPDA accumulated at following time points (Fig. 3C and D; Supplementary Fig. S2), indicating that *d_5_*-OPDA-Ile and *d_5_*-OPDA-Val are hydrolyzed to *d_5_*-*cis*-OPDA, and rapidly converted into JA/JA-Ile. In parallel, several other *d_5_*-OPDA-aa were detected upon treatment with labeled OPDA-aa. Seedlings treated with *d_5_*-OPDA-Val accumulated *d_5_*-OPDA-Ile after 30 min, and *d_5_*-OPDA-Asp and *d_5_*-OPDA-Glu at following time points (Supplementary Fig. S2A), while only *d_5_*-OPDA-Glu was detected 24 h after treatment with *d_5_*-OPDA-Ile (Supplementary Fig. S2B), suggesting that not all the *d_5_*-*cis*-OPDA originated from the hydrolysis of the labeled OPDA-aa is channeled into the JA synthesis (Supplementary Fig. S2C).

Together, these findings showed that (±)-OPDA-aa exhibit *cis*-OPDA-like activity in a JA-Ile-dependent manner and exert their function upon hydrolysis of the amino acid moiety and subsequent conversion to JA-Ile.

### Members of the Groups II and III GH3 family conjugate *cis*-(±)-OPDA with amino acids in an enzymatic assay and in planta

Arabidopsis possesses 19 different GH3 proteins classified into 3 groups (I, II, and III) based on substrate specificity and sequence homology (Staswick et al. 2005). To study how *cis*-(±)-OPDA is conjugated, we first tested the enzymatic activity of Arabidopsis Group I GH3s with *cis*-(±)-OPDA, as the GH3.10- and GH3.11-mediated JA conjugation is well-documented (Staswick and Tiryaki 2004; Delfin et al. 2022). Both recombinant enzymes could conjugate preferentially (±)-JA with Ile and, to a minor extent, with other amino acids (Supplementary Data Set 1 and Table S1), confirming published results (Staswick and Tiryaki 2004; Delfin et al. 2022). Surprisingly, none of these enzymes displayed activity toward *cis*-(±)-OPDA, and only a minimal amount above the limit of detection (LOD) of (±)-OPDA-Ile was found (Supplementary Data Set 1). To investigate whether GH3.11 could mediate conjugation of *cis*-OPDA in planta, we fed wild-type and *jar1-11* mutant seedlings with (±)-JA and *cis*-(±)-OPDA and inspected the formation of OPDA-aa after 3 h. While the accumulation of JA-aa, such as JA-Val and JA-Ile, was significantly reduced in *jar1-11* mutant upon treatment with (±)-JA compared with wild-type, as expected (Staswick and Tiryaki 2004; Delfin et al. 2022), OPDA-aa levels were not decreased in the *jar1-11* mutant (Supplementary Fig. S3), thus confirming that GH3.11 catalyzes the conjugation of JA but not of *cis*-OPDA.

**Figure 4.**
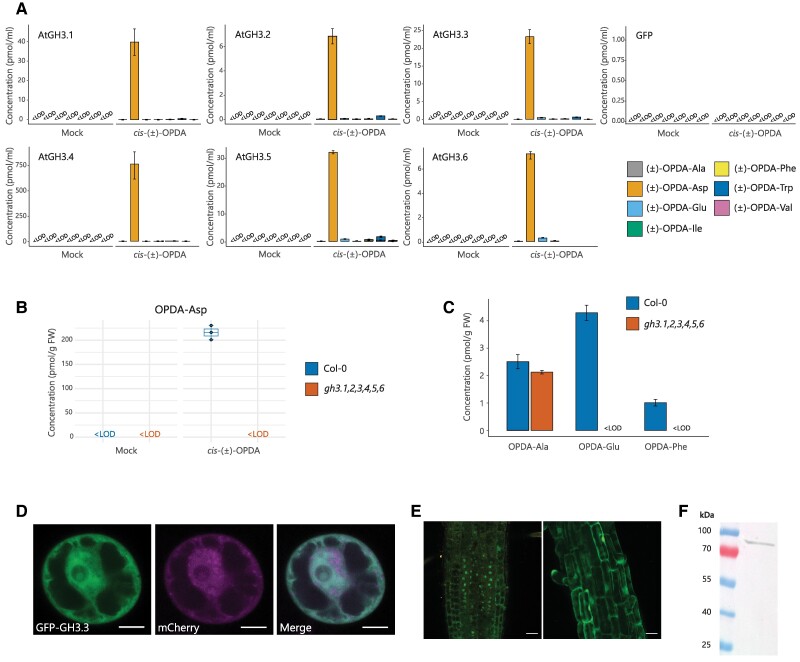
Members of the Group II GH3 family conjugate *cis*-OPDA with amino acids in planta. **A)** Analysis of (±)-OPDA-aa synthesized by recombinant Arabidopsis GH3.1, GH3.2, GH3.3, AtGH3.4, GH3.5, and GH3.6 in the bacterial assay. The cell lysate was incubated with or without 0.1 mm*cis*-(±)-OPDA and GH3 cofactor mixture for 5 h at 30 °C. The bacterial assay carried out with cell lysate from GFP-producing bacteria was used as a negative control. Cell lysate without *cis*-(±)-OPDA and cofactor mixture was used as a mock sample. (±)-OPDA-aa level is expressed as pmol/mL. The conjugation assay was performed in triplicate and repeated 3 times with similar results. **B)** Formation of OPDA-Asp after feeding of 7-d-old Arabidopsis Col-0 and *gh3* sextuple mutant (*gh3.1*,*gh3.2*,*gh3.3*,*gh3.4*,*gh3.5*,*gh3.6*) with or without 50 µM *cis*-(±)-OPDA for 3 h. OPDA-Asp level is expressed as pmol/g FW. Horizontal lines in the box plots are medians, boxes show the upper and lower quartiles, and whiskers show the entire data range. **C)** Accumulation of indicated OPDA-aa in Col-0 and *gh3* sextuple mutant after leaf wounding. Six-week-old plants were wounded, and damaged leaves were collected 2 h postwounding. **D**, **E)** GH3.3 has dual cytoplasmic/nuclear localization. **D)** Subcellular colocalization of GFP–GH3.3 fusion protein (left panel) with marker for cytosol/nucleus (mCherry; middle panel) in Arabidopsis root culture protoplasts. The right panel shows the overlay of the GFP–GH3.3 and mCherry signals. **E)** Confocal images of roots from 7-d-old Arabidopsis transgenic *UBQ10-GFP–GH3.3* seedlings, showing cortex cells (left) and epidermal cells in the mature root region (right). Confocal microscopy was performed on the third generation of at least 8 stable transgenic lines derived from different first-generation plants, with a consistent subcellular localization pattern observed across all lines. Scale bars in **D)** and **E)** represent 20 µm. **F)** Western Blot analysis of GFP–GH3.3 fusion protein in *GFP–GH3.3* overexpression Arabidopsis plants using anti-GFP antibody. The predicted molecular weight of the GH3.3 fusion protein is 84.5 kDa (GFP: 27 kDa; GH3.3: 67.5 kDa). Ala, Alanine; Asp, aspartate; Glu, glutamate; Ile, isoleucine; Phe, phenylalanine; Trp, tryptophan; Val, valine. Mean ± Sd (*n* = 3). Below the limit of detection, <LOD.

We then investigated whether the other members of the GH3 family could conjugate *cis*-(±)-OPDA with amino acids. Among Group II, known to conjugate mainly auxins (Staswick et al. 2005; Brunoni et al. 2023), GH3.1, GH3.2, GH3.3, GH3.4, GH3.5, and GH3.6 displayed the highest activity with *cis*-(±)-OPDA and Asp, and GH3.17 conjugated mainly *cis*-(±)-OPDA with Glu (Fig. 4A; Supplementary Fig. S4A). Among Group III, AtGH3.12 could conjugate *cis*-(±)-OPDA with Glu primarily and secondly with Val, Ile, Phe, and Trp, whereas AtGH3.14 and AtGH3.15 displayed a very similar activity in forming (±)-OPDA-Ala, (±)-OPDA-Trp, and (±)-OPDA-Ile primarily, and to a lesser extent (±)-OPDA-Phe (Supplementary Fig. S4A). Notably, among the GH3s catalyzing the conjugation of *cis*-(±)-OPDA, only those belonging to Group III were found highly active toward (±)-JA (Supplementary Data Set 1 and Table S1), supporting previous findings (Westfall et al. 2016; Sherp et al. 2018). To assess whether GH3s conjugate *cis*-OPDA in plants and to overcome gene redundancy interference, we fed wild-type Col-0 and *gh3.1,2,3,4,5,6* (*gh3* sextuple) mutant seedlings with *cis*-(±)-OPDA and followed the formation of OPDA-aa after 3 h. Similar levels of the OPDA-aa were recorded in the wild-type and the *gh3* sextuple mutant (Supplementary Fig. S4B), except for OPDA-Asp, whose formation was abolished entirely in the *gh3* sextuple mutant (Fig. 4B). To assess whether these conjugating enzymes contributed to the formation of OPDA-aa in stress responses, we carried out an OPDA-aa profiling of *gh3* sextuple mutant upon leaf wounding. OPDA-Glu and OPDA-Phe were not detected in *gh3* sextuple mutant 2 h postwounding, while these compounds accumulated in the wild-type Col-0 (Fig. 4C). On the contrary, OPDA-Ala equally accumulated in the mutant and the wild-type (Fig. 4C).

Altogether, these data indicate that GH3.1, GH3.2, GH3.3, GH3.4, GH3.5, and GH3.6 are the GH3 enzymes uniquely responsible for the conjugation of *cis*-OPDA with Asp in Arabidopsis plants, while the formation of other OPDA-aa results from the activity of GH3.17 and members of the Group III. Nonetheless, GH3.1–6 enzymes are also determinants for the accumulation of OPDA-Glu and OPDA-Phe in response to leaf wounding.

The endoplasmic reticulum (ER) and the cytosol have been hypothesized for a long time as compartments of activity of GH3 enzymes (Barbez and Kleine-Vehn 2013). Nonetheless, only direct proof of subcellular localization in the cytosol has been reported for the Glu-conjugating enzyme GH3.17 thus far (Di Mambro et al. 2019). To investigate the intracellular localization of Asp-conjugating Group II GH3s, we first transiently expressed a GFP–GH3.3 fusion construct in Arabidopsis protoplasts (Fig. 4D). GFP–GH3.3 colocalized with the cytosolic/nuclear mCherry marker, indicating that GH3.3 localizes in the cytosol and nucleus. The dual localization was further corroborated in roots of Arabidopsis transgenic plants stably expressing *pUBQ10-GFP–GH3.3* (Fig. 4E) and immunoblotting confirmed that the GFP-derived signal originated from GFP–GH3.3 fusion protein and not free GFP (Fig. 4F). These results suggest that the cytoplasm and nucleus are potential sites for GH3 activity.

### Members of the ILR1/ILL family hydrolyze OPDA-aa in an enzymatic assay and in planta upon wounding

**Figure 5.**
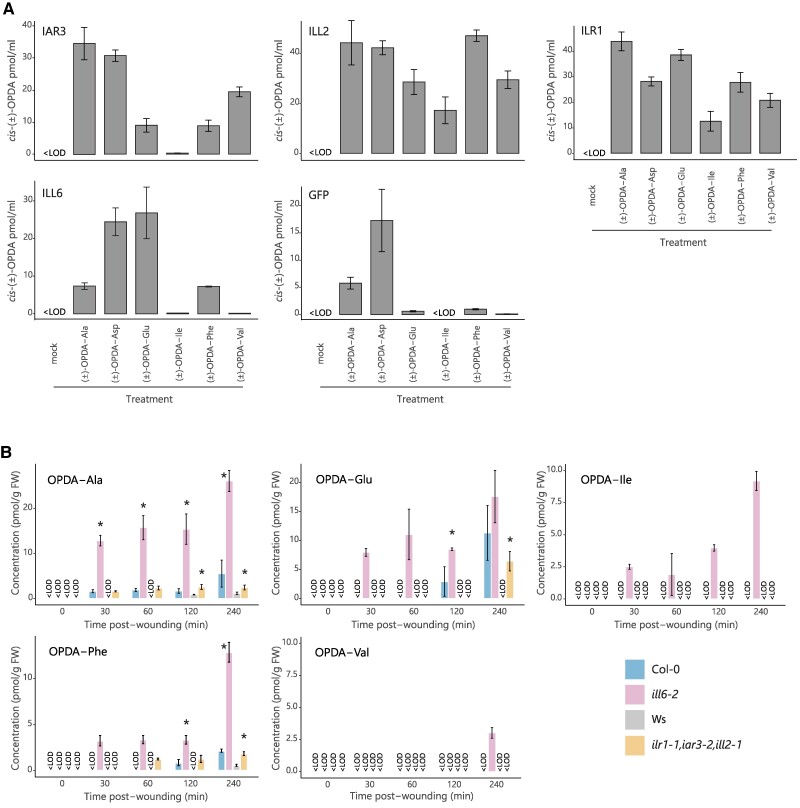
ILR1/ILL enzymes are involved in *cis*-OPDA release from OPDA-aa in Arabidopsis upon wounding. **A)** Analysis of release of *cis*-(±)-OPDA by recombinant IAR3, ILL2, ILR1, and ILL6 in the bacterial assay. The cell lysate was incubated with or without 0.1 mm (±)-OPDA-aa and 1 mm MgCl_2_ for 5 h at 30 °C. The bacterial assay carried out with the cell lysate from GFP-producing bacteria was used as a negative control. Cell lysate without (±)-OPDA-aa and MgCl_2_ was used as a mock sample. *cis*-(±)-OPDA level is expressed as pmol/mL. The hydrolysis assay was performed in triplicate and repeated 3 times with similar results. **B)** Time-course accumulation of indicated OPDA-aa in wild-types (Col-0, Ws), *ill6-2* single (Col-0 background), and *ilr1-1*,*iar3-2*,*ill2-1* triple (Ws background) knockout mutants after leaf wounding. Six-week-old plants were wounded, and damaged leaves were collected after the indicated times. Asterisk indicates statistically significant differences, as determined by Student's *t*-test (Col-0 vs *ill6-2*, Ws vs *ilr1-1,iar3-2,ill2-1*; *P* < 0.05). OPDA-aa concentrations are given as pmoles per gram FW. Ala, Alanine; Asp, aspartate; Glu, glutamate; Ile, isoleucine; Phe, phenylalanine; Val, valine. Mean ± Sd (*n* = 3). Below the limit of detection, <LOD.

Results from the feeding experiment with deuterium-labeled OPDA-aa showed that OPDA-aa can be hydrolyzed in planta (Fig. 3C and D). A subset of JA-inducible amidohydrolases of the ILR1/ILL family was previously described to catalyze the hydrolysis of JA-aa upon wounding in Arabidopsis leaves (Widemann et al. 2013; Zhang et al. 2016). Therefore, in the following experiments, we investigated the possible involvement of Arabidopsis ILR1, IAR3, ILL2, and ILL6 in the cleavage of OPDA-aa. To study their catalytic activities, recombinant Arabidopsis amidohydrolases were produced in *Escherichia coli*, and conditions for the hydrolysis assay were first determined by incubating the recombinant protein-producing cell lysate with IAA-Ala, confirming previously published results (Supplementary Data Set 1; LeClere et al. 2002; Zhang et al. 2016). Next, the activity of these 4 hydrolases was tested against (±)-OPDA-aa. ILL2 and ILR1 hydrolyzed all 6 (±)-OPDA-aa comparably well (Fig. 5A). While ILL2 displayed the highest activity toward (±)-OPDA-Ala, within 5 h which was tested as a proper time, (±)-OPDA-Asp, and (±)-OPDA-Phe, ILR1 hydrolyzed preferentially (±)-OPDA-Ala, (±)-OPDA-Asp, and (±)-OPDA-Glu. IAR3 was able to hydrolyze primarily (±)-OPDA-Ala, (±)-OPDA-Asp, and (±)-OPDA-Val, and to a lesser extent (±)-OPDA-Glu and (±)-OPDA-Phe. ILL6 showed the highest activity toward (±)-OPDA-Glu and secondly (±)-OPDA-Phe. The observed activity toward (±)-OPDA-Ala and (±)-OPDA-Asp could not be clearly ascribed to the recombinant amidohydrolases, as similar *cis*-(±)-OPDA levels were recorded in the GFP-producing bacteria after incubation with (±)-OPDA-Ala and (±)-OPDA-Asp, suggesting the possible presence of an endogenous substrate-associated bacterial machinery (Brunoni et al. 2019a). This enzymatic activity study showed that all the 4 tested members of the ILR1/ILL family can cleave several OPDA-aa, highlighted overlapping but distinct substrate specificities for various amino acid conjugates of *cis*-(±)-OPDA and predicted their possible involvement in *cis*-OPDA homeostasis.

**Figure 6.**
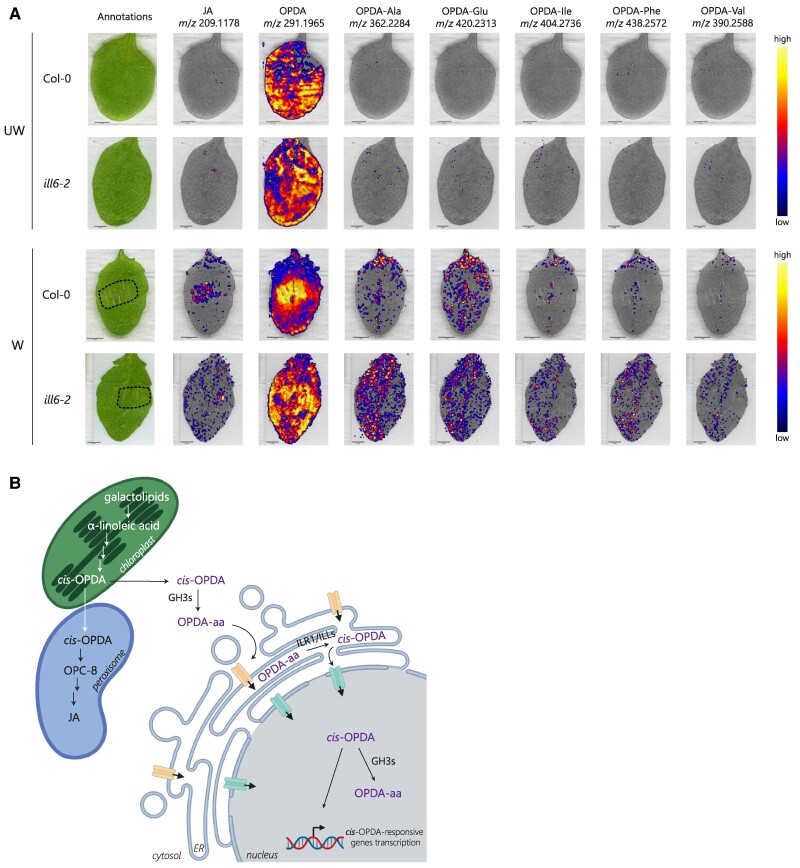
*In situ* visualization of OPDA-aa from wounded leaves and model for *cis*-OPDA and OPDA-aa subcellular pathways. **A)** DESI-MSI images showing the localization of OPDA-aa in Col-0 and *ill6-2* knockout mutant in wounded (W) and unwounded (UW) leaves. Three-week-old plants were wounded, and damaged leaves were collected after 4 h. Annotated images of the JA, OPDA, OPDA-Ala, OPDA-Glu, OPDA-Ile, OPDA-Phe, and OPDA-Val distribution in control (top) and wounded leaves (bottom) are reported. The wounded region in damaged leaves is delimited with a dashed line. The peak intensity levels are displayed on the scale at the right side of the panels. Scale bars in the insets represent 2 mm. Ala, Alanine; Asp, aspartate; Glu, glutamate; Ile, isoleucine; Phe, phenylalanine; Val, valine. **B)** Proposed model for *cis*-OPDA and OPDA-aa subcellular pathways. Once synthesized in the chloroplast, *cis*-OPDA is mainly reduced and β-oxidized to produce OPC-8 and JA in the peroxisome. Part of the chloroplast-derived *cis*-OPDA reaches the cytosol, where GH3 enzymes conjugate it with amino acids to form OPDA amino acid conjugates (OPDA-aa). OPDA amide conjugates are hydrolyzed back to *cis*-OPDA in the ER by ILR1/ILL enzymes and *cis*-OPDA is then released in the nucleus. Nuclear *cis*-OPDA can activate the expression of *cis*-OPDA-responsive genes or be conjugated with amino acids by GH3s. According to this model, *cis*-OPDA amino acid conjugation might be an essential step in regulating nuclear *cis*-OPDA levels through the ER. Unknown transporters likely mediate OPDA-aa fluxes among the cytosol, the ER, and the nucleus. Jasmonic acid, JA; 3-oxo-2-(2-(*Z*)-pentenyl)-cyclopentane-1-octanoic, OPC-8.

To assess whether these hydrolases contributed to regulating the OPDA-aa levels in plants, we carried out a time-course OPDA-aa profiling on *ill6-2* single and *ilr1-1*,*iar3-2,ill2-1* triple knockout mutants upon leaf wounding. OPDA-Ala, OPDA-Glu, OPDA-Ile, and OPDA-Phe accumulated in *ill6-2* mutant already 30 min postwounding; their level was always higher than Col-0 throughout the time course, whereas OPDA-Val was detected only in the *ill6-2* mutant as its level was always below the LOD in the Col-0 (Fig. 5B). Considering the low hydrolytic activity of recombinant ILL6 against OPDA-Ala and OPDA-Ile, it was surprising that these 2 OPDA-aa accumulated in the *ill6-2* mutant (Fig. 5A and B). Nonetheless, a similar discrepancy between in vitro and in vivo assays was already reported for ILL6 (Zhang et al. 2016). OPDA-Ala and OPDA-Phe were detected in the *ilr1-1*,*iar3-2*,*ill2-1* triple mutant 30 min and 1 h after wounding, respectively, and their level was steady over time and greater in the triple mutant than Ws. OPDA-Glu was found 4 h after injury only in the triple mutant, and no accumulation in Ws was found. OPDA-Asp was not detected in any of the mutant lines or the wild-types. Moreover, leaf wounding of the *ilr1-1*, *iar3-2*, and *ill2-1* single knockout mutants showed that OPDA-Ala and OPDA-Phe accumulated in all 3 single mutants. In contrast, OPDA-Glu was detected only in the *iar3-2* and *ill2-1* mutants (Supplementary Fig. S5). Considering that OPDA-aa are endogenous low abundant *cis*-OPDA derivatives only formed upon stress and rapidly hydrolyzed back to *cis*-OPDA in response to wounding, their formation might be localized at the damaged site of the leaf. To explore this hypothesis, we monitored the spatial distribution of endogenous OPDA-aa in wounded leaves from Col-0 and *ill6-2* mutant using desorption electrospray ionization mass spectrometry imaging (DESI-MSI), a technique that was previously adopted for the in situ visualization of jasmonates in wounded Arabidopsis leaves (Zhang et al. 2021). Representative MS/MS spectra of endogenous JA, OPDA, OPDA-Ala, OPDA-Glu, OPDA-Ile, and OPDA-Val acquired from the wounded leaf samples are shown in Supplementary Fig. S6, and their major fragmentation ions are included with their molecular formula. As expected, OPDA-aa were detected only in wounded leaves, and no signal was recorded in the control undamaged leaves (Fig. 6A). We found that OPDA-aa were equally distributed in the wounded and unwounded areas of Col-0 and *ill6-2* mutant damaged leaves 4 h postwounding (Fig. 6A), with OPDA-Ile and OPDA-Val only detected in wounded leaves of *ill6-2* mutant, confirming that these OPDA-aa are detectable upon wounding only in the absence of ILL6 (Fig. 5B). Nevertheless, *cis*-OPDA already evenly distributed on the whole leaf surface of Col-0 and *ill6-2* mutant unwounded leaves (Fig. 6A). Notably, upon wounding, *cis*-OPDA accumulated predominantly at the damaged site in Col-0 leaves, whereas the *cis*-OPDA distribution was widespread all over the leaf in the *ill6-2* mutant, albeit the leaf damage induced an increase of *cis*-OPDA level in both genotypes. This suggests that ILL6 is required to channel *cis*-OPDA at the damaged site in response to wounding.

Altogether, these findings suggest that ILR1, IAR3, ILL2, and ILL6 are all involved in the hydrolysis of several OPDA-aa and that OPDA-aa are rapidly cleaved by these amidohydrolases upon wounding.

## Discussion

Conjugation of *cis*-OPDA to amino acids has been reported to occur in plants, as OPDA-Ile and OPDA-Asp were identified in Arabidopsis wounded leaves and in chitooligosaccharide-treated rice cell culture, respectively (Floková et al. 2016; Shinya et al. 2022). Although it was proposed that these conjugates might play a role in forming *cis*-OPDA active ligands, the mechanism by which OPDA-aa are formed, their role and function in plants remain elusive and poorly understood. Here, we investigated the occurrence and meaning of *cis*-OPDA amino acid conjugation using a liquid chromatography (LC)–MS/MS-based method to study OPDA-aa formation under different physiological conditions that alter *cis*-OPDA and JA homeostasis in plants. Our results show that both biotic and abiotic stress elicits the conjugation of *cis*-OPDA to amino acids in Arabidopsis, very likely because of increased *cis*-OPDA levels (Fig. 1; Supplementary Fig. S1). We further demonstrated that the root-growth inhibition mediated by (±)-OPDA-Val, (±)-OPDA-Ile, (±)-OPDA-Phe, and (±)-OPDA-Ala, requires functional conversion of JA into JA-Ile, thus hydrolysis of these OPDA-aa to *cis*-OPDA might be needed to exert their function. On the contrary, (±)-OPDA-Asp and (±)-OPDA-Glu were found ineffective in inhibiting root growth (Fig. 2A and B). In fact, the first group of OPDA-aa-induced OPDA-responsive genes equally to *cis*-OPDA, while the second group did not (Fig. 3A). To address the possible conjugate hydrolysis, we performed a feeding assay using isotopically labeled OPDA-Ile and OPDA-Val and corroborated that both conjugates are cleaved into *cis*-OPDA and converted to OPC-4, JA, and JA-Ile by plants already 30 min after application (Fig. 3C and D; Supplementary Fig. S2). Notably, not all the *cis*-OPDA deriving from the OPDA-aa hydrolysis is channeled into the JA biosynthesis. Instead, it can be conjugated with Glu, Asp, and Ile (Supplementary Fig. S2). Collectively, these findings and the confirmation that OPDA-Ile is not a ligand for functional COI1–JAZ coreceptors (Fig. 3B) suggest that OPDA-aa direct the *cis*-OPDA to catabolism or storage for subsequent hydrolysis, instead of acting as chemical signals per se as originally speculated for OPDA-Ile (Arnold et al. 2016).

Further biochemical and physiological analyses were conducted to investigate the pathway for OPDA-aa formation and hydrolysis. We addressed the possible involvement of members of the GH3 and the ILR1/ILL families known to catalyze amino acid conjugation of acidic phytohormones and conjugate hydrolysis, respectively. Functional analysis of the Arabidopsis GH3 enzymes combined with in planta-feeding assay of *gh3* knockout mutants revealed that the JA-conjugating GH3s are not involved in the conjugation of *cis*-OPDA (Supplementary Data Set 1 and Fig. S3), OPDA-Asp formation depends exclusively on the activity of the IAA-conjugating enzymes, and members of the Group III GH3s are likely involved in the conjugation of *cis*-OPDA with the other amino acids (Fig. 4A and B; Supplementary Fig. S4A and Data Set 1). Moreover, GH3 enzymes from Groups II and III feasibly act redundantly in conjugating of endogenous *cis*-OPDA after wounding (Fig. 4C).

Three enzymes, ILR1, ILL6, and IAR3 of the ILR1/ILL family, were previously identified as JA-amidohydrolases that are induced upon wounding (Widemann et al. 2013; Zhang et al. 2016). Here, we undertook a functional analysis of JA-amidohydrolases and ILL2 coupled with an in vivo study of their activity in wounding response and showed that ILR1, IAR3, ILL6, and ILL2 are *cis*-OPDA-amidohydrolases and redundantly contribute to regulating active *cis*-OPDA level upon leaf damage (Fig. 5; Supplementary Fig. S5). Altogether, given that the levels of accumulated OPDA-aa in the stress responses inspected in this study are much less than that of JA-Ile, we have to consider homeostasis of OPDA-aa as an additional determinant in different stress responses and developmental processes.

Considering the low abundance of OPDA-aa and the tissue-specific and developmentally controlled expression of *GH3* and *ILR1/ILL* genes (Rampey et al. 2004; http://bar.utoronto.ca/), the regulation of *cis*-OPDA levels mediated by amido synthetases and amidohydrolases could be restricted to specific tissue types (e.g. in the damaged site of plants) upon wounding or pathogen infection. We investigated this hypothesis by in situ DESI-MSI detection of endogenous OPDA-aa in wounded leaves. OPDA-aa did not exclusively localize in the damaged area of wild-type and *ill6-2* mutant leaves but equally accumulated all over the leaf (Fig. 6A). On the whole, the characteristic expression patterns of *GH3* and *ILR1/ILL* genes and the finding that *cis*-OPDA is a mobile signal that can be relocated from wounded shoots into undamaged roots (Schulze et al. 2019) could imply that the OPDA-aa formation and hydrolysis may become more critical for localized *cis*-OPDA increase/decrease in distal sites other than harmed leaves. Furthermore, a possible translocation of endogenous OPDA-aa is a hypothesis that should be evaluated.

GH3 enzymes localize in the cytosol and the nucleus (Fig. 4D and E; Di Mambro et al. 2019), while ILR1/ILL enzymes in the ER (Zhang et al. 2016). Thus, part of the *cis*-OPDA synthesized in the chloroplast must access the cytosol. Our data reveal that not all the *cis*-OPDA synthesized in the chloroplast is metabolized in the peroxisome or binds to CYP20-3 in the chloroplast stroma. Cytosolic *cis*-OPDA imported into peroxisomes was already postulated to represent a parallel “leak” pathway for the passive import alternative to the CTS-dependent active transport (Theodoulou et al. 2005). Besides, the dual cytoplasmic/nuclear localization of GH3s (Fig. 4D and E) further corroborates that the *cis*-OPDA presence is not restricted to chloroplasts and peroxisomes. Given that the COI1-dependent expression of *IAR3*, *ILL6*, and *ILR1* genes in the first few hours postwounding (Zhang et al. 2016), and the accumulation of OPDA-aa already 30 min after wounding in *ill6-2* and *ilr1-1,iar3-2,ill2-1* knockout mutants (Fig. 5B), it is likely that the rapid synthesis and hydrolysis of OPDA-aa coordinately contributes to the modulation of the *cis*-OPDA homeostasis, which in turn can be depleted in favor of JA biosynthesis in the peroxisome, catabolized in the cytosol/nucleus or trigger its autonomous pathway. The existence of such a level of regulation provides further evidence that some of the chloroplast-derived *cis*-OPDA “leaks” in the cytosol, where it can be temporarily conjugated to amino acids, thus likely revoking *cis*-OPDA downstream effects (Fig. 6B). However, the hypothetical activity of the conjugation of *cis*-OPDA with amino acids as reactive electrophile species, such as *cis*-OPDA, should be further investigated.

Oppositely to JA, and similarly to IAA, amino acid conjugation of *cis*-OPDA is not required for plant hormone activation, and OPDA-aa serve as reservoirs of inactive *cis*-OPDA. Accordingly, OPDA-aa are hydrolyzed by ILR1/ILLs in the ER. Consequently, free *cis*-OPDA can reach the nucleus, acting as a signaling molecule or be inactivated by GH3 enzymes. Thus, an ER-to-nucleus flux of *cis*-OPDA might underpin nuclear *cis*-OPDA uptake (Fig. 6B). Overall, our findings confirm the existence of a homeostatically regulated *cis*-OPDA pool between the cytosol and the ER and hint at a subcellular pathway that might directly control nuclear *cis*-OPDA levels by ER-to-nucleus flux of *cis*-OPDA. Further study on the proposed framework would shed insight into *cis*-OPDA intracellular routes.

This work points to the contribution of synthesis and hydrolysis of OPDA-aa to the temporary storage of *cis*-OPDA. Thus, *cis*-OPDA, similar to other signaling molecules, including IAA and JA, appear to share a similar level of regulation that relies on standard metabolic machinery to modulate the homeostasis of the active phytohormone. Interestingly, conjugation of the signaling molecule dn-*iso*-OPDA with different amino acids was also found to deactivate the hormone in bryophytes and lycophytes (Liang et al. 2024), suggesting that the inactivation of the OPDA hormonal activity mediated by the conjugative pathway is not restricted to flowering plants but rather is ancestral.

### Limitations of the study

There is a significant limitation inherent to studying the endogenous occurrence of OPDA-aa in plants due to the low abundance of these compounds, which are not detected under steady-state growth conditions but only upon stress. In this study, we inspected the endogenous accumulation of OPDA-aa in response to biotic and abiotic stress using 2 different mass spectrometry techniques. OPDA-Ile was not detected in wounded 6-wk-old plants by LC–MS/MS but was identified in mechanically stressed 3-wk-old leaves by DESI-MSI, although its signal was extremely low. Differences in the experimental conditions of wounding experiments may have caused different endogenous levels of OPDA-Ile detected by the LC–MS/MS and DESI-MSI methods, respectively. Moreover, MS-based measurements can be affected by higher matrix effects, potentially obscuring low-abundance compounds such as OPDA-Ile. Both techniques have their strengths for determination in a complex plant matrix and can complement each other depending on the needs of the analysis. However, DESI-MSI can sometimes raise questions about signal specificity. It is necessary to mention that high-resolution measurements, determination of exact analyte masses, and subsequent MS/MS fragmentation guarantee unequivocal identification. On the other hand, LC–MS/MS typically offers high specificity due to the separation step in LC, but surprisingly did not demonstrate sufficient sensitivity in our experimental setup.

Fluorescent tagging of proteins and confocal imaging techniques have become routine in cellular and subcellular localization studies. Here, we have investigated the intracellular localization of fluorescently tagged GH3.3 protein by both transiently and stably overexpressing it in Arabidopsis protoplasts and root epidermis, respectively, with both strategies returning the same GFP–GH3.3 subcellular localization pattern. Nonetheless, when expressed ectopically at high levels, both stable and transient expression methods might give rise to the mislocalization of the target protein. Thus, a comparative study, including the expression of the fluorescently-tagged GH3.3 protein driven by the native promoter and the impact of the tag on the localization signal, should also be assessed in further experiments as a proof of concept.

## Materials and methods

### Plant material

*Arabidopsis thaliana* ecotype Col-0 was used as wild-type for all the experiments, except for Fig. 5B and Supplementary Fig. S5, where Wassilewskija (Ws) was also used. Knockout lines in the Col-0 background used were *jar1-11* (SALK_034543), *gh3.10-2,jar1-11* (Delfin et al. 2022) *gh3* sextuple (*gh3.1*,*gh3.2*,*gh3.3*,*gh3.4*,*gh3.5*,*gh3.6*; Porco et al. 2016), and *ill6-2* (SALK_024894) whereas in the Ws background were *ilr1-1*,*iar3-2*,*ill2-1* triple and corresponding single mutants (Bartel and Fink 1995; Davies et al. 1999; Rampey et al. 2004).

### Mechanical wounding

Arabidopsis plants were grown in soil under neutral-day conditions (12 h light/12 h dark) in cultivation chambers maintained at 21 °C, with a light intensity of ∼150 μmol m^−1^ s^−1^ and 40% to 60% relative humidity. Wounding was conducted on fully expanded rosette leaves of 6-wk-old plants by crushing across the midvein 3 times using forceps (Widemann et al. 2013). At increasing time points following mechanical damage, leaf samples were quickly harvested and flash-frozen in liquid nitrogen before storing at −80 °C until use. Wounded leaves were pooled from 3 to 4 plants for each sample, and the experiment was repeated 3 times (*n* = 3).

### Fungal infection analysis

Arabidopsis Col-0 seeds were gas sterilized with chlorine gas (10 mL of 35% [v/v] HCl in 50 mL of bleach) for at least 2 h in an airtight box. Seeds were then sown under sterile conditions on Petri dishes containing ½ Murashige–Skoog (½ MS) medium (2.15 g salts including vitamins per 1 L) with 1% sucrose and 0.5 g L^−1^ MES monohydrate at pH 5.7 and solidified with 0.8% (w/v) Gellan gum. Stratification was carried out for 3 d at 4 °C and then, plates were transferred to light at 22 ± 1 °C, under long-day conditions (16 h light/8 h dark; 100 µmol^−2^ s^−1^) and grown vertically. Wild-type *B. cinerea* CCF2361 isolated from a garden strawberry (Department of Botany, Faculty of Science, Charles University, Czech Republic) was grown on potato-dextrose agar (PDA; HiMedia Laboratories) at 22 °C. After 3 wk, spores were collected with sterile distilled water and filtered through glass wool (Sigma-Aldrich). Two-week-old in vitro-grown Arabidopsis seedlings were inoculated with a 10-µL drop of fungal spores (17.10 × 6 spores/mL); infected plants were harvested 4 d postinoculation and flash-frozen in liquid nitrogen before storing at −80 °C until LC–MS/MS analysis. About 16 infected Arabidopsis seedlings per replicate were used, and the experiment was repeated 3 times (*n* = 3).

### Feeding experiments

Plant growth conditions and feeding experiments of Arabidopsis were carried out as described in Brunoni et al. (2023). For feeding with unlabeled standards, 7-d-old Arabidopsis seedlings were cultivated in liquid ½ MS medium supplemented with or without 50 µM (±)-JA, *cis*-(±)-OPDA, (±)-OPDA-Ala, (±)-OPDA-Val, (±)-OPDA-Phe, (±)-OPDA-Asp, (±)-OPDA-Glu, and (±)-OPDA-Phe for 3 h, (±)-OPDA-aa were prepared following (Mik et al. 2023). The feeding experiment was repeated 3 times (*n* = 3).

For feeding with standards labeled with stable isotopes, 7-d-old Arabidopsis seedlings were cultivated in liquid ½ MS medium supplemented with 10 µM *d_5_*-OPDA-Val and *d_5_*-OPDA-Ile. One milliliter of liquid medium and 10 mg FW of plant samples were collected after 30 min, 3, and 24 h. *d_5_*-OPDA-aa were synthesized as described in Supplementary Note S1. The feeding experiment was repeated 3 times (*n* = 3).

### Root-growth inhibition assay

Three-day-old Col-0 and *gh3.10-2,jar1-11* Arabidopsis seedlings germinated in ½ MS vertical plates under the conditions described above were transferred onto vertical ½ MS medium in the presence of 10 µM (±)-JA, *cis*-(±)-OPDA, (±)-OPDA-Ala, (±)-OPDA-Val, (±)-OPDA-Phe, (±)-OPDA-Asp, (±)-OPDA-Glu, and (±)-OPDA-Phe for 4 d under long-day conditions (16 h light/8 h dark) in cultivation chambers maintained at 21 °C, with a light intensity of ∼100 μmol m^−2^ s^−1^ and 60% relative humidity. Root length of 14 to 15 seedlings was measured 7 d after germination. Plates were scanned using a scanner (Epson Perfection V550 Photo), and roots were quantified using ImageJ/Fiji software. Three independent biological replicates were measured for each sample (*n* = 3). Data were analyzed by 1-way ANOVA/Tukey HSD post hoc test (*P* < 0.05).

### Promoter-GUS assay

Arabidopsis seeds of *35S:JAZ1-GUS* marker line (Thines et al. 2007) were germinated and vertically grown in the conditions described above. Seven-day-old in vitro-grown seedlings were treated with or without 10 µM (±)-JA, *cis*-(±)-OPDA, (±)-OPDA-Ala, (±)-OPDA-Val, (±)-OPDA-Phe, (±)-OPDA-Asp, (±)-OPDA-Glu, and (±)-OPDA-Phe for 2 h and the visualization of GUS was carried out as described in Chini et al. (2018). Root imaging was carried out with a camera (QuickPHOTO CAMERA 3.2) connected to a stereo microscope (Olympus DP72). The experiment was repeated twice with similar results.

### RNA extraction and reverse transcription quantitative PCR

Total RNA was isolated using Spectrum Total RNA kit (Sigma-Aldrich), and DNA-free DNA Removal Kit (Invitrogen) was used to prepare DNA-free RNA according to the manufacturer's instructions. One microgram of total RNA was reverse transcribed for each sample with RevertAid H Minus Reverse Transcriptase (Thermo Scientific). Reverse transcription quantitative PCR (RT-qPCR) was performed on a CFX384 Touch Real-Time PCR Detection System (Bio-Rad) using 2× SYBR Green Real-Time PCR Master Mix (Applied Biosystems). The 3-step cycling program was as follows: 95 °C for 2 min, followed by 40 cycles at 95 °C for 5 s, 60.5 °C for 20 s, and 72 °C for 10 s. Melting curve analysis was conducted between 75 and 95 °C. The *ACT2* (AT3G18780) gene was used as a constitutive internal standard to normalize the obtained gene expression results. Primer sequences are listed in Supplementary Table S2. Expression levels were calculated using the ΔΔCt method (Pfaffl 2001). Three biological replicates were performed for each test (*n* = 3). Data were analyzed by 1-way ANOVA/Tukey HSD post hoc test (*P* < 0.05).

### Pull-down assay

For the pull-down experiments using fluorescein-tagged JAZ peptides (Fl-AtJAZPs), purified GST-AtCOI1 (5 nm), Fl-AtJAZP (10 nm; Takaoka et al. 2018, 2019), and each compound, JA-Ile (1 µM) or OPDA-Ile (1 or 30 µM) in 350 µL of incubation buffer (50 mm Tris-HCl buffer, pH 7.8, 100 mm NaCl, 10% [v/v] glycerol, 0.1% [v/v] Tween20, 100 nm inositol-1,2,4,5,6-pentakisphosphate [IP5]) were combined with antifluorescein antibody (0.2 µL, GeneTex, GTX26644, USA), and incubated for 10 to 15 h at 4 °C with rotation. After incubation, the samples were combined with SureBeads Protein G (10 µL in 50% [v/v] incubation buffer slurry, Bio-Rad, USA). After 3 h of incubation at 4 °C with rotation, the samples were washed 3 times with 350 µL of wash buffer (phosphate-buffered saline containing 0.1% [v/v] tween20). The washed beads were resuspended in 35 µL of SDS–PAGE loading buffer containing DTT (100 mm). After heating for 10 min at 60 °C, the samples were subjected to SDS–PAGE and analyzed by western blotting. The bound GST–COI1 protein was detected using anti-GST-HRP conjugate (RPN1236, GE Healthcare, USA), 5,000-fold dilution in blocking buffer (Nakalai Tesque, Inc., Japan).

### Cloning, protein production, and bacterial enzyme assay

*Escherichia coli* BL21 (DE3) strains expressing recombinant GH3s used in this work were previously generated (Brunoni et al. 2019a; 2023). Recombinant protein production and enzymatic assay of GH3s were performed as described previously (Brunoni et al. 2023). IAR3, ILL2, ILL6, and ILR1 open reading frame sequences deleted of the 25-N-terminal signal peptide-encoding codons were PCR-amplified from cDNA by adding *Bsa*I restriction sites using *Phusion* Taq Polymerase (Thermo Fisher) and the primer sequences listed in Supplementary Table S2 prior to cloning into pETM11 plasmid in the BL21 (DE3) *E. coli* strain. Recombinant protein production was performed as described by Brunoni et al. (2019a). For conjugation assay, 500 µL of clarified cell lysate from GH3-producing bacterial cultures was incubated with GH3 cofactors and with or without 0.1 mm (±)-JA and *cis*-(±)-OPDA for 5 h at 30 °C with constant shaking at 50 rpm in darkness. The conjugation assay was performed in triplicate and repeated 3 times with similar results (*n* = 3). For hydrolysis assay, 500 µL of clarified cell lysate from amidohydrolase-producing bacterial cultures was incubated with 1 mm MgCl_2_ and 0.1 mm IAA-Ala, (±)-OPDA-Ala, (±)-OPDA-Val, (±)-OPDA-Phe, (±)-OPDA-Asp, (±)-OPDA-Glu, and (±)-OPDA-Ile in the same conditions as those for the conjugation assay. The hydrolysis assay was performed in triplicate and repeated 3 times with similar results (*n* = 3). (±)-JA and *cis*-(±)-OPDA were purchased from Olchemim, (±)-OPDA-aa were prepared following (Mik et al. 2023), and IAA-Ala was kindly provided by Dr. Asta Žukauskaitė. Vectors used and generated are listed in Supplementary Table S3.

### DNA vectors for subcellular localization study

Constructs for plant transformation were produced by GreenGate cloning (Lampropoulos et al. 2013). The coding sequence of *GH3.3* was PCR-amplified from cDNA by adding *Bsa*I restriction sites and cloned into the empty module C using the primers listed in Supplementary Table S2. The generated C-module in combination with A-module harboring either *p35S* or *pUBQ10*, B-module (*eGFP*), D-module (dummy sequence), E-module (*rcbs* terminator), and F-module (hygromycin resistance) were used to build *pGREEN-IIS* final vectors *p35S-GFP–GH3.3* and *pUBQ10-GFP–GH3.3*. Vectors used and generated are listed in Supplementary Table S3.

### Transient expression in root suspension culture protoplasts

Protoplasts were isolated from 4-d-old Arabidopsis *Ler* root suspension culture and transfected by the PEG method as described by Hurný et al. (2020). Protoplasts were diluted with B5 glucose-mannitol solution to a final concentration of 4 × 10^6^ protoplasts/mL. Two micrograms of DNAs (Wave1R; Geldner et al. 2009 and *p35S-GFP–GH3.3*) were mixed together with 50 μL of protoplast suspension and 60 μL of PEG solution (0.1 m Ca(NO_3_)_2_, 0.45 m mannitol, 25% [w/v] PEG 6000) and incubated in the dark for 16 h. Then 140 µL of 0.275 m Ca(NO_3_)_2_ solution was added to wash off PEG, wait for sedimentation of protoplasts, and remove 240 µL of supernatant. The protoplast pellet was resuspended in 200 µL of B5 glucose-mannitol solution and incubated for 16 h in the dark at room temperature. Transfected protoplasts were mounted on the slides and viewed with Zeiss LSM 900 confocal scanning microscope.

### Plant transformation

To generate *GFP–GH3.3* transgenic Arabidopsis lines, the *pUBQ10-GFP–GH3.3* final construct was introduced by electroporation in *Agrobacterium tumefaciens* and then transformed into Arabidopsis Col-0 by the floral dip method. Transformants were selected on plates containing 15 µg/mL hygromycin.

### Protein extraction and western blot

Approximately 300 mg of frozen ground plant material was mixed with 1 mL of extraction buffer (150 mm NaCl, 50 mm Tris-HCl pH 7.5, 0.1% [v/v] Tween 20, 10% glycerol, 1 mm DTT, 1 mm Pefabloc SC [Roche], 1× Protease Inhibitor Cocktail [Roche], incubated in ice for 1 h, and clarified by centrifugation at 14,000 rpm for 45 min at 4 °C). Proteins were precipitated by mixing 200 µL of supernatant with methanol–chlorophorm, precipitated total protein was resuspended in 8 m urea and 4× Laemmli sample buffer (Bio-Rad). Samples were loaded on 12% (w/v) SDS/PAGE gel and, after gel run, transferred to a PVDF membrane by wet electro blotting. For the detection of recombinant proteins, the PVDF membrane was blocked in 1× TBS-T with 5% skimmed milk for 2 h at room temperature. After blocking, the membrane was incubated with anti-GFP antibody horseradish peroxidase conjugate (Miltenyi Biotech, 1: 10,000 dilution in 1× TBS-T with 5% skimmed milk) for 2 h at room temperature and washed for 10 min, 4 times, in 1× TBS-T. Signals were detected by an enhanced chemiluminescent (ECL) detection system (GE Healthcare RPN2232). ECL signals were captured by a charge-coupled device camera (Azure c600).

### Confocal imaging and image analysis

Zeiss LSM 900 confocal scanning microscope using either ×20 or ×40 (water immersion) objectives were employed to monitor the expression of fluorescent reporters. eGFP and mCherry signals were detected either at 488 nm excitation/509 nm emission or 587 nm excitation/610 nm emission wavelengths, respectively. For Fig. 4D, the lasers excitation wavelength (intensity) 488 nm (0.8%) and 587 nm (0.2%); the detector gain 664 and 650 V; detection wavelength 410 to 546 and 595 to 700. For Fig. 4E (left panel), laser excitation wavelength (intensity) 488 (4%); detector gain 845 V; detection wavelength 410 to 546; (right panel), laser excitation wavelength (intensity) 488 (4%); detector gain 839 V; detection wavelength 410 to 546. Zeiss ZEN blue software was used for image processing.

### Chemical synthesis, liquid chromatography tandem mass spectrometry phytohormone measurements, and DESI-MSI

Detailed procedures for synthesis, phytohormone measurements and in situ visualization of OPDA-aa are reported in Supplementary Note S1.

### Accession numbers

Sequence data from this article can be found in the GenBank/EMBL data libraries under accession numbers listed in Supplementary Table S4.

## Supplementary Material

kiae636_Supplementary_Data

## Data Availability

The data that support the findings of this study are available on request from the corresponding authors.

## Supplementary data

The following materials are available in the online version of this article.

**Supplementary Figure S1**. Accumulation of *cis*-OPDA, JA, and JA-aa in Arabidopsis plants during stress responses.

**Supplementary Figure S2**. Conversion of OPDA-aa into JA-Ile and *cis*-OPDA derivatives.

**Supplementary Figure S3**. Amino acid conjugation of *cis*-OPDA is not mediated by JAR1/GH3.11 *in planta*.

**Supplementary Figure S4**. Conjugating activity with *cis*-(±)-OPDA of recombinant GH3s and accumulation of OPDA-aa in gh3 sextuple mutant upon *cis*-(±)-OPDA feeding.

**Supplementary Figure S5**. Accumulation of OPDA-aa in ilr1/ill single knockout mutants upon wounding.

**Supplementary Figure S6**. In situ DESI-MSI/MS spectra of JA, OPDA, OPDA-Ala, OPDA-Glu, OPDA-Ile, OPDA-Phe, and OPDA-Val from wounded leaves.

**Supplementary Table S1**. Semi-quantitative comparison of enzymatic activities with (±)-JA and amino acids of recombinant *A. thaliana* GH3 proteins resulting from the bacterial assay.

**Supplementary Table S2**. List of primers sequences used for qPCR analysis and cloning.

**Supplementary Table S3**. List of vectors used and generated in this study.

**Supplementary Table S4**. List of accession numbers of sequence data of genes/proteins mentioned in this study in order of appearance.

**Supplementary Data Set 1**. Raw mass spectrometry data of (±)-OPDA-aa and (±)-JA-aa with recombinant GH3 enzymes, and *cis*-(±)-OPDA and IAA with recombinant ILR1/ILL enzymes from the bacterial assay.

**Supplementary Note S1**. Synthetic procedures, phytohormone measurements, and DESI-MSI.

## Dive Curated Terms

The following phenotypic, genotypic, and functional terms are of significance to the work described in this paper:

ILL6 Gramene: AT1G44350
ILL6 Araport: AT1G44350

ILR1 Gramene: AT3G02875
ILR1 Araport: AT3G02875

IAR3 Gramene: AT1G51760
IAR3 Araport: AT1G51760

ILL2 Gramene: AT5G56660
ILL2 Araport: AT5G56660

GRX480 Gramene: AT1G28480
GRX480 Araport: AT1G28480

JAZ1 Gramene: AT1G19180
JAZ1 Araport: AT1G19180

JAZ5 Gramene: AT1G17380
JAZ5 Araport: AT1G17380

ZAT10 Gramene: AT1G27730
ZAT10 Araport: AT1G27730

OPR3 Gramene: AT2G06050
OPR3 Araport: AT2G06050

COI1 Gramene: AT2G39940
COI1 Araport: AT2G39940
