# Supplementary material for: Amide conjugates of the jasmonate precursor cis-(+)-12-oxo-phytodienoic acid regulate its homeostasis during plant stress responses
Source: Plant Physiol. 2024 Nov 28;197(1):kiae636. doi: 10.1093/plphys/kiae636 (PMC11663710; doi:10.1093/plphys/kiae636)
Supplement: kiae636_Supplementary_Data [file kiae636_supplementary_data.zip › Supplementary Figures 16 Supplementary Tables 14.pdf]

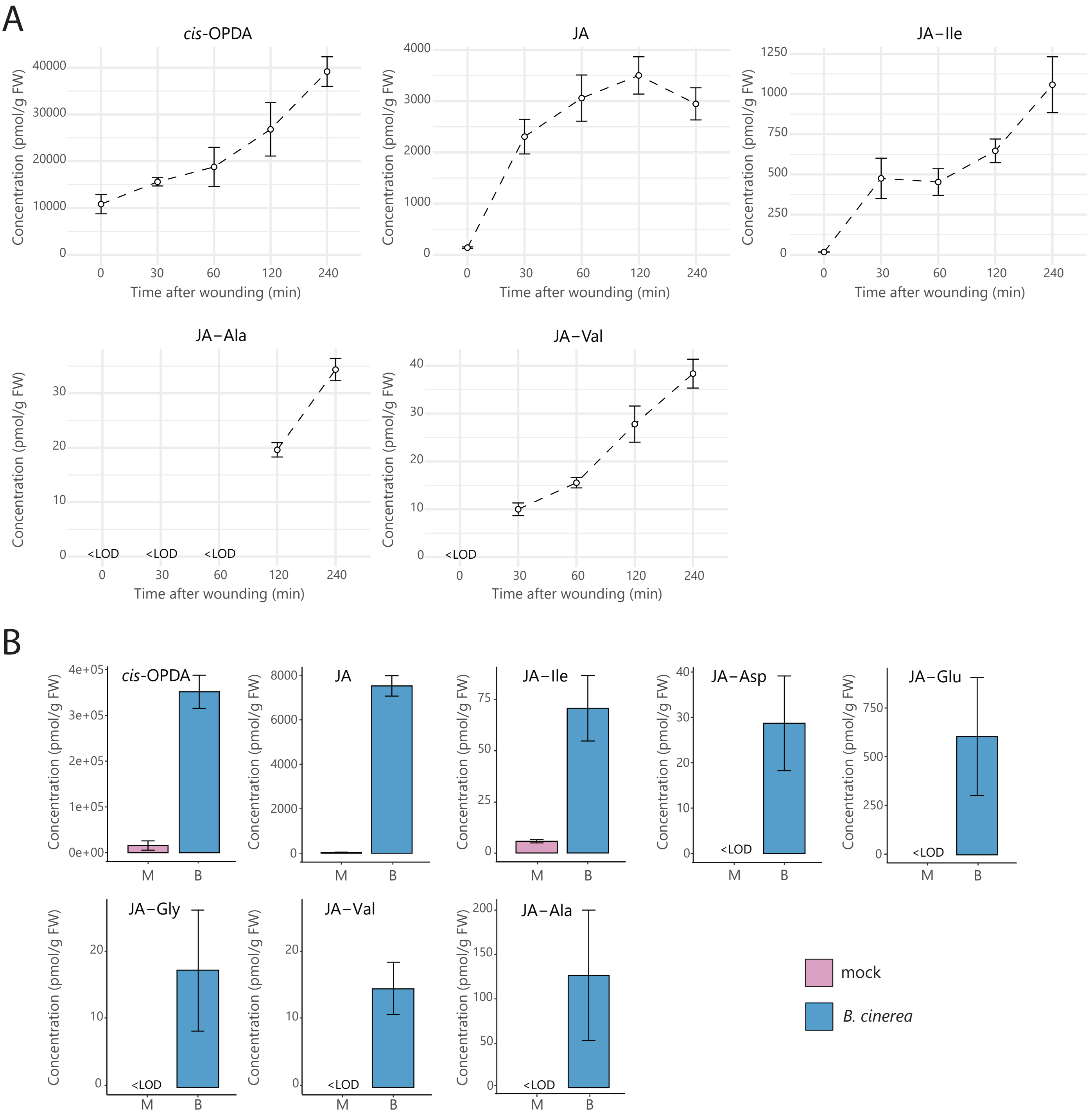

Supplementary Figure S1 Accumulation of *cis*-OPDA, JA, and JA-aa in Arabidopsis plants during stress responses. (A) Time-course accumulation of *cis*-OPDA, JA, and indicated JA-aa in Col-0 after leaf wounding. Six-week-old plants were wounded, and damaged leaves were collected at the indicated times. (B) Accumulation of *cis*-OPDA, JA, and indicated JA-aa in Col-0 plants infected with *Botrytis cinerea* 4 days after inoculation (B) or mock-inoculated (M).

Jasmonic acid, JA; isoleucine, Ile; aspartate, Asp; glutamate, Glu; glycine, Gly; valine, Val; alanine, Ala. Metabolite levels are expressed as pmoles per gram fresh weight (FW). Mean ± SD (n=3). Below the limit of detection, <LOD.

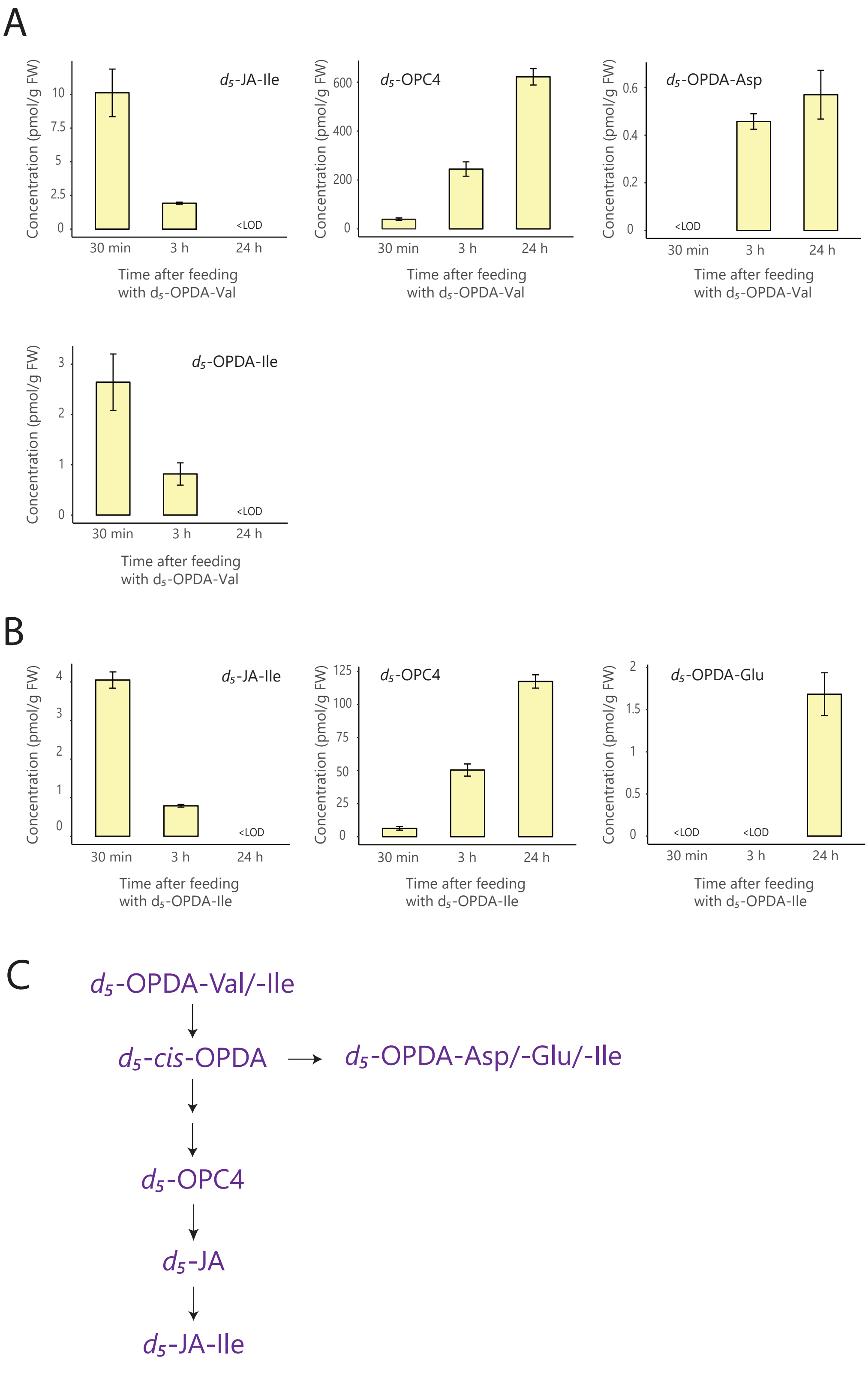

Supplementary Figure S2 Conversion of OPDA-aa into JA-Ile and *cis*-OPDA derivatives. (A, B) Time-course accumulation of stable isotope-labeled derivatives JA-Ile, OPC4, OPDA-Asp, OPDA-Glu, and OPDA-Ile of OPDA-Val and OPDA-Ile in seven-day-old Col-0 seedlings after feeding with 10 µM d_5-OPDA-Val (A) and d_5-OPDA-Ile (B). Samples were collected at the indicated times. Metabolite levels are expressed as pmoles per gram fresh weight (FW). Mean ± SD (n=3). Below the limit of detection, <LOD. (C) Scheme of the *in planta* metabolic conversion of stable isotope-labeled OPDA-Val and OPDA-Ile.

Jasmonic acid, JA; 3-oxo-2-(2-(*Z*)-pentenyl)-cyclopentane-1-butanoic acid, OPC-4; aspartate, Asp; glutamate, Glu; isoleucine, Ile; valine, Val.

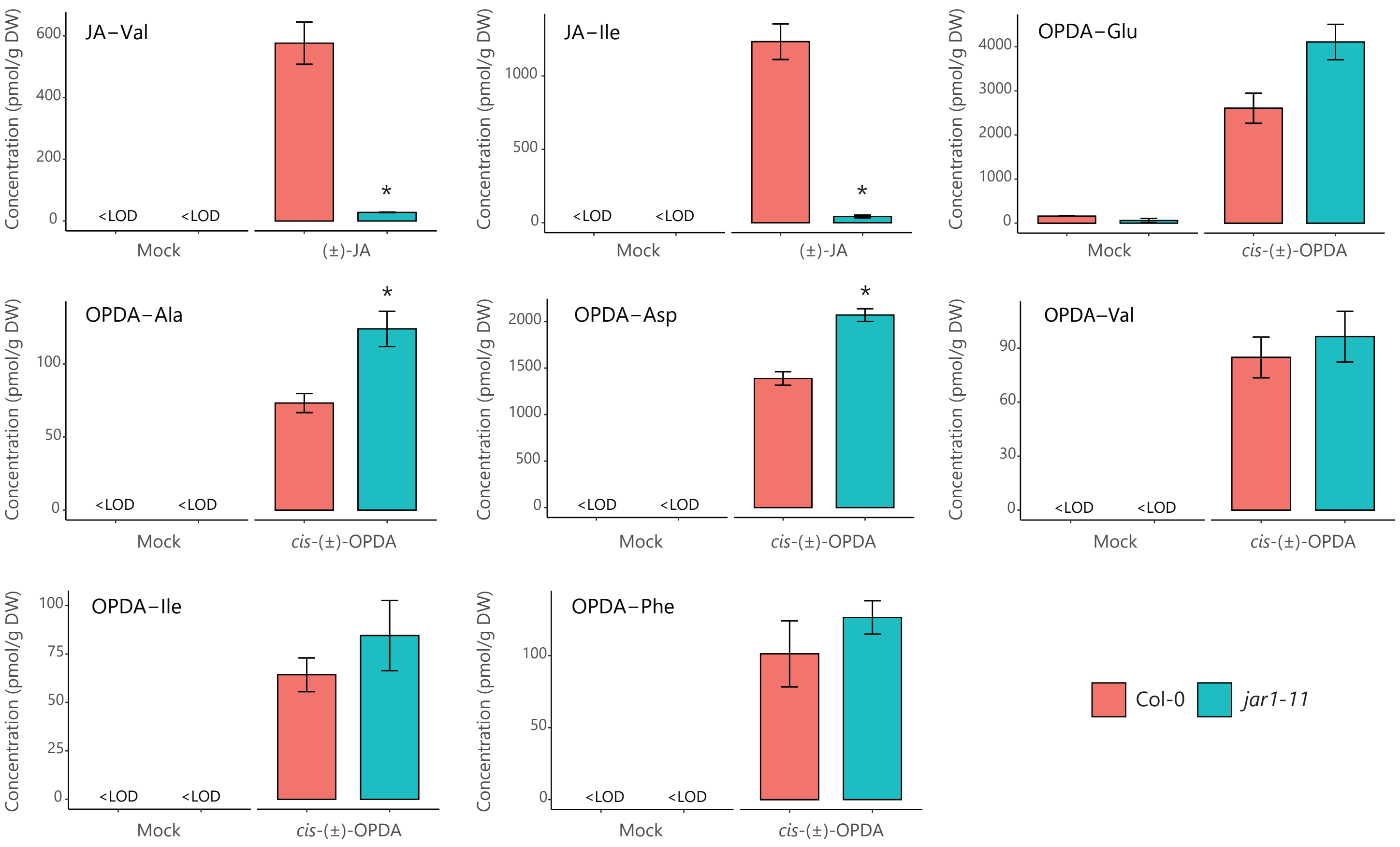

Supplementary Figure S3 Amino acid conjugation of *cis*-OPDA is not mediated by JAR1/GH3.11 *in planta*. Accumulation of indicated JA-aa and OPDA-aa after exogenous treatment with or without 50 µM (±)-JA and *cis*-(±)-OPDA in *jar1-11* mutant. Asterisk indicates statistically significant differences, as determined by Student's *t*-test (Col-0 vs *jar1-11*; $P<0.05$). Jasmonic acid, JA; alanine, Ala; aspartate, Asp; glutamate, Glu; isoleucine, Ile; phenylalanine, Phe; valine, Val. Metabolite concentrations are given as pmoles per gram dry weight (DW). Mean ± SD (n=3). Below the limit of detection, <LOD.

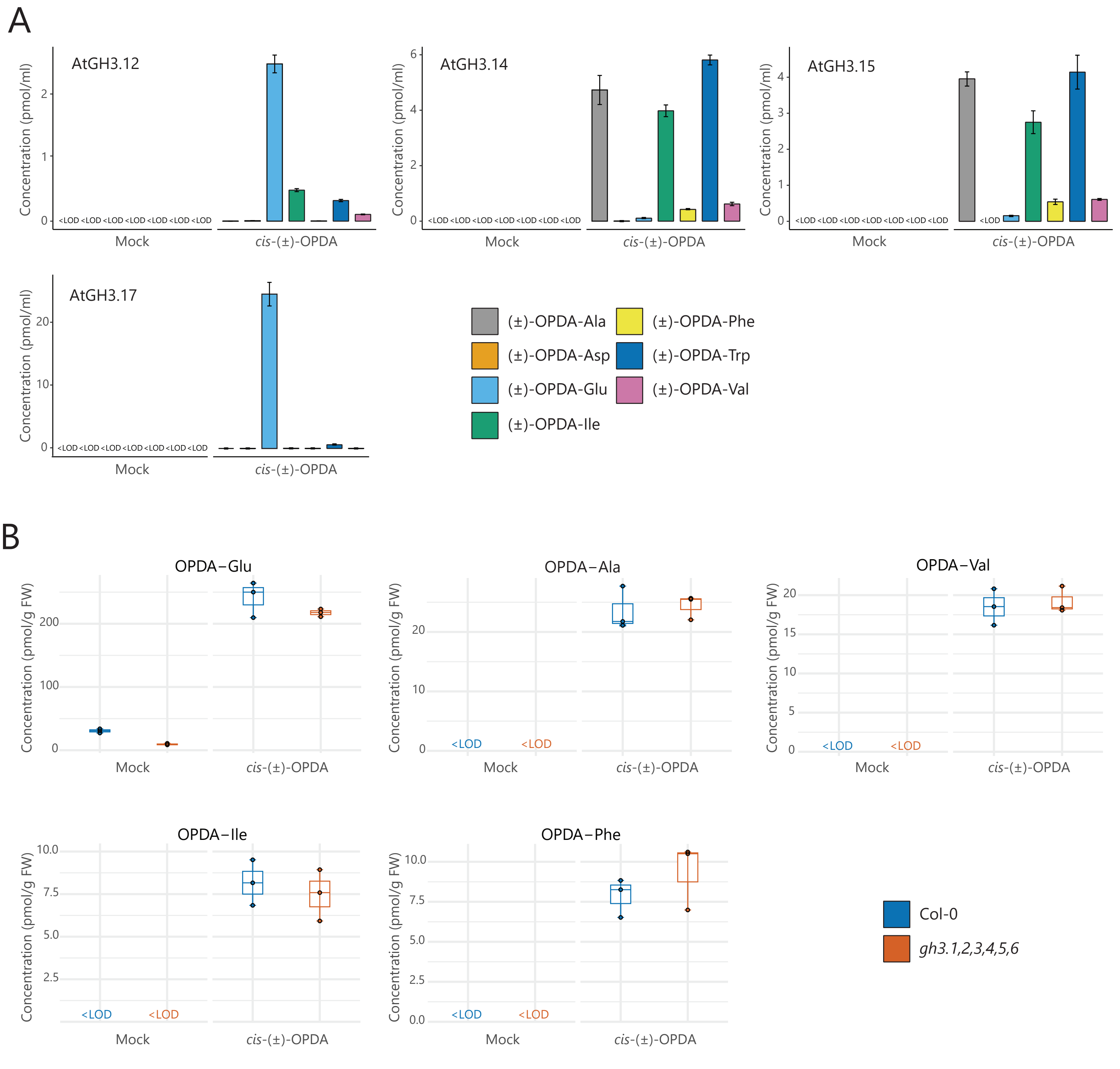

Supplementalry Figure S4 Conjugating activity with *cis*-(±)-OPDA of recombinant GH3s and accumulation of OPDA-aa in *gh3* sextuple mutant upon *cis*-(±)-OPDA feeding. (A) Analysis of OPDA-aa synthesized by recombinant GH3.12, GH3.14, GH3.15, and GH3.17 in the bacterial assay. The cell lysate was incubated with or without 0.1 mM *cis*-(±)-OPDA and GH3 cofactor mixture for 5 h at 30 °C. The bacterial assay carried out with cell lysate from GFP-producing bacteria was used as a negative control. Cell lysate without *cis*-(±)-OPDA and cofactor mixture was used as a mock sample. OPDA-aa level is expressed as pmol/ml. The conjugation assay was performed in triplicate and repeated three times with similar results. (B) Formation of OPDA-Glu, OPDA-Ala, OPDA-Val, OPDA-Ile, and OPDA-Phe after feeding of 7-day-old Arabidopsis Col-0 and *gh3* sextuple mutant (*gh3.1,gh3.2,gh3.3,gh3.4,gh3.5,gh3.6*) with or without 50 μM *cis*-(±)-OPDA for 3 h. OPDA-aa concentration is given as pmoles per gram fresh weight (FW). Horizontal lines in the box plots are medians, boxes show the upper and lower quartiles, and whiskers show the full data range. Alanine, Ala; aspartate, Asp; glutamate, Glu; isoleucine, Ile; phenylalanine, Phe; valine, Val; tryptophan Trp. Mean ± SD (n=3). Below the limit of detection, <LOD.

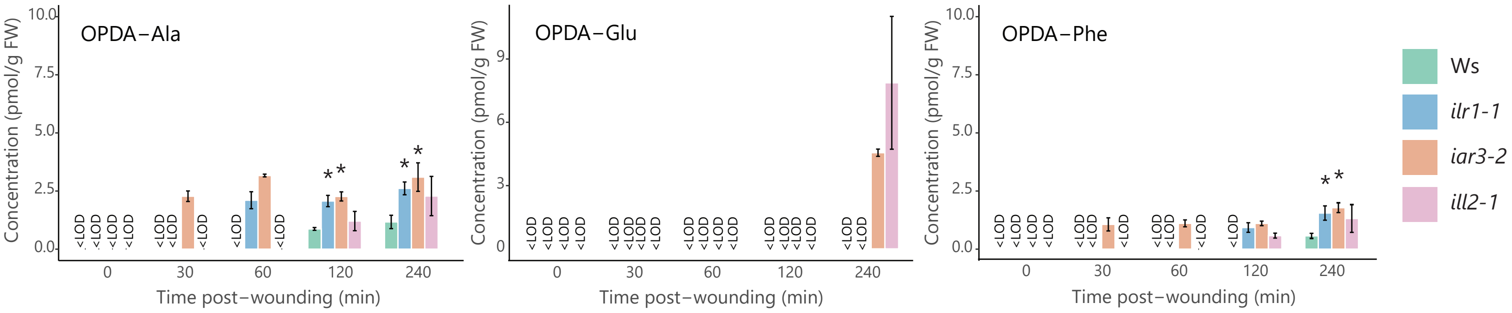

Supplementary Figure S5 Accumulation of OPDA-aa in *ilr1*/*ill* single knockout mutants upon wounding. Time-course accumulation of indicated OPDA-aa in Ws, *ilr1-1*, *iar3-2*, and *ill2-1* single knockout mutants after leaf wounding. Six-week-old plants were wounded, and damaged leaves were collected after the indicated times. Asterisk indicates statistically significant differences, as determined by Student's *t*-test (Ws vs *ilr1-1*, *iar3-2*, or *ill2-1*; $P<0.05$). OPDA-aa concentrations are given as pmoles per gram fresh weight (FW). Alanine, Ala; glutamate, Glu; phenylalanine, Phe. Mean ± SD ($n=3$). Below the limit of detection, <LOD.

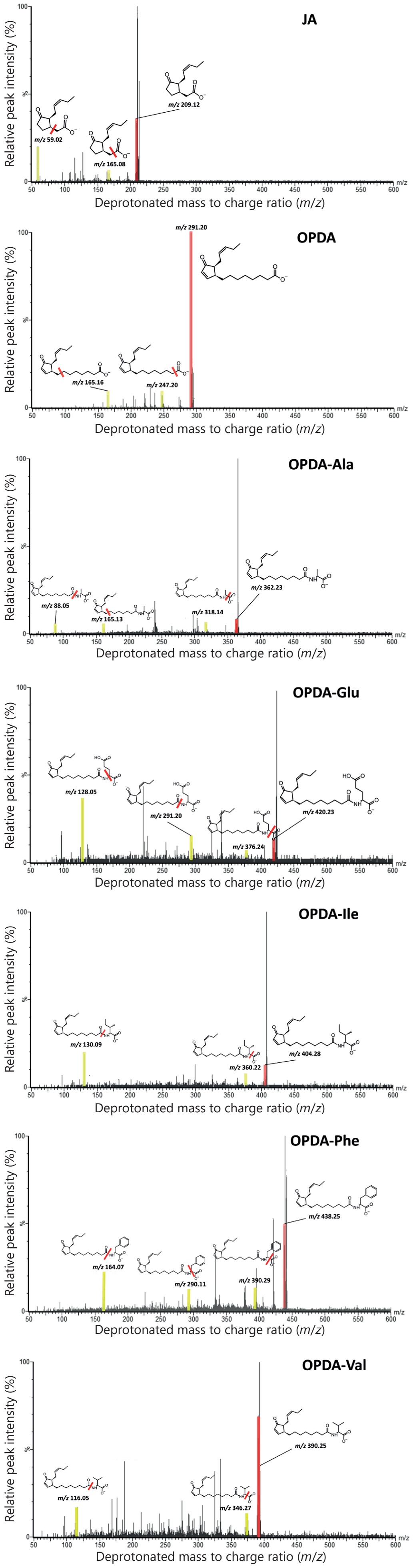

Supplementary Figure S6 *In situ* DESI-MSI/MS spectra of JA, OPDA, OPDA-Ala, OPDA-Glu, OPDA-Ile, OPDA-Phe, and OPDA-Val from wounded leaves. Red and yellow bars indicate the precursor and fragmentation ion peaks, respectively. Jasmonic acid, JA; alanine, Ala; glutamate, Glu; isoleucine, Ile; phenylalanine, Phe; valine, Val.

Supplementary Table S1 Semi-quantitative comparison of enzymatic activities with (±)-JA and amino acids of recombinant *Arabidopsis thaliana* GH3 proteins resulting from the bacterial assay.

Species	GH3	Group	Σ(+)-JA-Gly/ (-)-JA-Gly	Σ(+)-JA-Glu/ (-)-JA-Glu	Σ(+)-JA-Asp/ (-)-JA-Asp	(+)-JA-Ala	(-)-JA-Ala	(+)-JA-Val	(-)-JA-Val	(+)-JA-Met	(-)-JA-Met	Σ(+)-JA-Trp/ (-)-JA-Trp	(+)-JA-Ile	(-)-JA-Ile	(+)-JA-Phe	(-)-JA-Phe
Arabidopsis	GH3.1	II	–	–	–	–	–	–	–	–	–	–	–	–	–	–
	GH3.2	II	–	–	+	–	–	–	–	+	–	–	–	–	–	–
	GH3.3	II	–	–	+	–	–	–	–	–	–	–	–	–	–	–
	GH3.4	II	–	–	–	–	–	–	–	–	–	–	–	–	–	–
	GH3.5	II	–	–	–	–	–	–	–	–	–	–	–	–	–	–
	GH3.6	II	–	–	–	–	–	–	–	–	–	–	–	–	–	–
	GH3.7	III	+	+	–	++	–	+	–	+	–	++	+	–	–	–
	GH3.8	III	–	–	–	–	–	–	–	–	–	–	–	–	–	–
	GH3.9	II	–	–	–	–	–	–	–	–	–	–	–	–	–	–
	GH3.10	I	–	–	–	–	–	+	+	+	+	–	+++	+++	–	–
	GH3.11	I	–	–	–	+	+	++	++	+++	+ +	++	++++	++++	+++	+
	GH3.12	III	–	++	–	–	–	+	+	+	+	–	+	+ +	–	–
	GH3.13	III	–	–	–	–	–	–	–	–	–	–	–	–	–	–
	GH3.14	III	–	–	–	+	–	+	–	++	+	++	+	+	–	–
	GH3.15	III	+	++	–	+++	++	++	+	++++	+++	++++	+++	+	++	–
	GH3.16	III	–	–	–	–	–	–	–	–	–	–	–	–	–	–
	GH3.17	II	–	+	–	–	–	–	–	–	–	–	–	–	–	–
	GH3.19	III	–	–	–	–	–	–	–	–	–	–	–	–	–	–
Negative control	GFP		–	–	–	–	–	–	–	–	–	–	–	–	–	–

Range of detected (±)-JA-aa levels: < 0.1 pmol/mL (+), 0.1 – 1 pmol/mL (++), 1 – 10 pmol/mL (+++), > 10 pmol/mL (++++), and not detected (–).

Supplementary Table S2 List of primers sequences used for qPCR analysis and cloning.

Name	Gene ID	Purpose	Primer name	Sequence (5'->3')
GRX480	AT1G28480	qPCR	qGRX480_Fw	TGATTGTGATTGGACGGAGA
			qGRX480_Rv	TAAACCGCCGGTAACTTCAC
ZAT10	AT1G27730	qPCR	qZAT10_Fw	ATCAACACTAGTAGCGTGTCC
			qZAT10_Rv	AGTCAAACCGAGGCTTCTTCG
THI2.1	AT1G72260	qPCR	qTHI2.1_Fw	GTTGGGTAAACGCCATTCTCG
			qTHI2.1_Rv	GTGGGACTACATAGCTCTTGG
PDF1.2	AT5G44420	qPCR	qPDF1.2_Fw	CACCCTTATCTTCGCTGCTC
			qPDF1.2_Rv	GTTGCATGATCCATGTTTGG
JAZ5	AT1G17380	qPCR	qJAZ5_Fw	CCAAGCCAGAGATTGTAACCG
			qJAZ5_Rv	CGAGATCTTTCGAACTTTGGC
VSP1	AT5G24780	qPCR	qVSP1_Fw	TCTCATACTCAAGCCAAACGG
			qVSP1_Rv	AGTATCCTCAACCAAATCAGC
ACT2	AT3G18780	qPCR	qAtACTIN2_Fw	GACCAGCTCTTCCATCGAGAA
			qAtACTIN2_Rv	CAAACGAGGGCTGGAACAAG
IAR3	AT1G51760	Cloning in pETM11	IAR3_Δ25N_Fw	AACAGGTCTCACGCGTCCTCTAATGGGTTATCTCAA
			IAR3_Δ25N_Rv	AACAGGTCTCTATTATCAAAGTTCATCTTTTTTGTT
ILL6	AT1G44350	Cloning in pETM11	ILL6_Δ25N_Fw	AACAGGTCTCACGCGACCAACTTACCTTTCTTTGAA
			ILL6_Δ25N_Rv	AACAGGTCTCTATTATTATGAATGTTTATCATTTAA
ILR1	AT3G02875	Cloning in pETM11	ILR1_Δ25N_Fw	AACAGGTCTCACGCGTACGATTCTGGTTCGGGTCTC
			ILR1_Δ25N_Rv	AACAGGTCTCTATTACTATAATTCACTCTTAACCTCT
ILL2	AT5G56660	Cloning in pETM11	ILL2_Δ25N_Fw	AACAGGTCTCACGCGTGGATCGCCGAAGATACGTCT
			ILL2_Δ25N_Rv	AACAGGTCTCTATTATTAGAGTTCTTCATGAAAGCC
GH3.3	AT2G23170	Cloning in pGGC000	GH3.3_GG_Fw	AACAGGTCTCAGGCTCAACAATGACCGTTGATTCAGCTCTGCG
			GH3.3_GG_Rv	AACAGGTCTCTCTGATCAACGACGACGTTCTGGTGA

Supplementary Table S3 List of vectors used and generated in this study.

Name	Type	Description	Overhangs	Bacterial resistance	Reference
pGGA004	Plant promoter	*35S* (*Cauliflower mosaic virus 35S*; internal *Bsa*I site removed) promoter	A-B	Ampicillin	Lampropoulos *et al.*, 2013
pGGA004	Plant promoter	*UBQ10* (*UBIQUITIN10*) promoter	A-B	Ampicillin	Lampropoulos *et al.*, 2013
pSC147	N-tag	*eGFP-linker*	B-C	Ampicillin	Kindly donated by Dr. Silvio Collani
pGGC000	Empty entry vector	CDS	C-D	Ampicillin	Lampropoulos *et al.*, 2013
pGGD002	C-tag	*D-dummy* (default random sequence with stop codon)	D-E	Ampicillin	Lampropoulos *et al.*, 2013
pGGE001	Plant terminator	*RBCS* terminator (from pea)	E-F	Ampicillin	Lampropoulos *et al.*, 2013
pGGF005	Plant resistance cassette	*pUBQ10:HygrR:tOCS*	F-G	Ampicillin	Lampropoulos *et al.*, 2013
pGGZ003	Empty destination vector	Plant resistance at LB	A-G	Spectinomycin	Lampropoulos *et al.*, 2013
pGGC-GH3.3	Coding sequence	At*GH3.3* coding sequence in module C	C-D	Ampicillin	This study
p35S-GFP-GH3.3	Plant expression vector	*p35S:GFP::GH3.3cds:D-dummy:tRBCS:HygR*	A-G	Spectinomycin	This study
pUBQ10-GFP-GH3.3	Plant expression vector	*pUBQ10:GFP::GH3.3cds:D-dummy:tRBCS:HygR*	A-G	Spectinomycin	This study
Wave1R	Plant expression vector	*pUNI51* (mCherry only, cytosol/nucleus localization)	-	Kanamycin	Geldner *et al.*, 2009
pETM11-ccdB-cat	Empy entry vector	Vector for recombinant protein expression in *E. coli* harboring *Bsa*I-*ccdB-cat*-*Bsa*I	cgcg-/-atta	Kanamycin/ Chloramphenicol	Brunoni *et al.*, 2023
pETM11-Δ25N-AtILR1	Recombinant protein expression in *E. coli*	*pETM11::6xHis-Δ25N AtILR1cds:KanR*	cgcg-/-atta	Kanamycin	This study
pETM11-Δ25N-AtILL2	Recombinant protein expression in *E. coli*	*pETM11::6xHis-Δ25N AtILL2cds:KanR*	cgcg-/-atta	Kanamycin	This study

pETM11-Δ25N-AtILL6	Recombinant protein expression in *E. coli*	*pETM11::6xHis-Δ25N AtILL6cds:KanR*	cgcg-/-atta	Kanamycin	This study
pETM11-Δ25N-AtIAR3	Recombinant protein expression in *E. coli*	*pETM11::6xHis-Δ25N AtIAR3cds:KanR*	cgcg-/-atta	Kanamycin	This study
pETM11-AtGH3.1	Recombinant protein expression in *E. coli*	*pETM11::6xHis-AtGH3.1cds:KanR*	-	Kanamycin	Brunoni *et al.*, 2023
pETM11-AtGH3.2	Recombinant protein expression in *E. coli*	*pETM11::6xHis-AtGH3.2cds:KanR*	-	Kanamycin	Brunoni *et al.*, 2023
pETM11-AtGH3.3	Recombinant protein expression in *E. coli*	*pETM11::6xHis-AtGH3.3cds:KanR*	-	Kanamycin	Brunoni *et al.*, 2023
pETM11-AtGH3.4	Recombinant protein expression in *E. coli*	*pETM11::6xHis-AtGH3.4cds:KanR*	-	Kanamycin	Brunoni *et al.*, 2023
pETM11-AtGH3.5	Recombinant protein expression in *E. coli*	*pETM11::6xHis-AtGH3.5cds:KanR*	-	Kanamycin	Brunoni *et al.*, 2023
pETM11-AtGH3.6	Recombinant protein expression in *E. coli*	*pETM11::6xHis-AtGH3.6cds:KanR*	-	Kanamycin	Brunoni *et al.*, 2019
pETM11-AtGH3.7	Recombinant protein expression in *E. coli*	*pETM11::6xHis-AtGH3.7cds:KanR*	cgcg-/-atta	Kanamycin	Brunoni *et al.*, 2023
pETM11-AtGH3.8	Recombinant protein expression in *E. coli*	*pETM11::6xHis-AtGH3.8cds:KanR*	cgcg-/-atta	Kanamycin	Brunoni *et al.*, 2023
pETM11-AtGH3.9	Recombinant protein expression in *E. coli*	*pETM11::6xHis-AtGH3.9cds:KanR*	cgcg-/-atta	Kanamycin	Brunoni *et al.*, 2023
pETM11-AtGH3.10	Recombinant protein expression in *E. coli*	*pETM11::6xHis-AtGH3.10cds:KanR*	cgcg-/-atta	Kanamycin	Brunoni *et al.*, 2023
pETM11-AtGH3.11	Recombinant protein expression in *E. coli*	*pETM11::6xHis-AtGH3.11cds:KanR*	-	Kanamycin	Brunoni *et al.*, 2023
pETM11-AtGH3.12	Recombinant protein expression in *E. coli*	*pETM11::6xHis-AtGH3.12cds:KanR*	cgcg-/-atta	Kanamycin	Brunoni *et al.*, 2023
pETM11-AtGH3.13	Recombinant protein expression in *E. coli*	*pETM11::6xHis-AtGH3.13cds:KanR*	cgcg-/-atta	Kanamycin	Brunoni *et al.*, 2023
pETM11-AtGH3.14	Recombinant protein expression in *E. coli*	*pETM11::6xHis-AtGH3.14cds:KanR*	cgcg-/-atta	Kanamycin	Brunoni *et al.*, 2023

pETM11-AtGH3.15	Recombinant protein expression in *E. coli*	*pETM11::6xHis-AtGH3.15cds:KanR*	cgcg-/-atta	Kanamycin	Brunoni *et al.*, 2023
pETM11-AtGH3.16	Recombinant protein expression in *E. coli*	*pETM11::6xHis-AtGH3.16cds:KanR*	cgcg-/-atta	Kanamycin	Brunoni *et al.*, 2023
pETM11-AtGH3.17	Recombinant protein expression in *E. coli*	*pETM11::6xHis-AtGH3.17cds:KanR*	-	Kanamycin	Brunoni *et al.*, 2019
pETM11-AtGH3.19	Recombinant protein expression in *E. coli*	*pETM11::6xHis-AtGH3.19cds:KanR*	cgcg-/-atta	Kanamycin	Brunoni *et al.*, 2023
pETM11-GFP	Recombinant protein expression in *E. coli*	*pETM11::6xHis-GFP:KanR*	-	Kanamycin	Brunoni *et al.*, 2019

References:

Lampropoulos A, *et al.* (2013). GreenGate-a novel, versatile, and efficient cloning system for plant transgenesis. PloS one 8: e83043.
Geldner N, *et al.* (2009) Rapid, combinatorial analysis of membrane compartments in intact plants with a multicolor marker set. Plant J 59:169-178.
Brunoni F, *et al.* (2023) Amino acid conjugation of oxIAA is a secondary metabolic regulation involved in auxin homeostasis. New Phytol 238: 2264-2270.
Brunoni F, *et al.* (2019) A bacterial assay for rapid screening of IAA catabolic enzymes. Plant Methods 15: 126.

Supplementary Table S4 List of accession numbers of sequence data of genes/proteins mentioned in this study in order of appearance.

Name	Locus	GenBank accession
LOX1	AT1G55020	AY093104
LOX2	AT3G45140	NM_114383
LOX3	AT1G17420	NM_101603
LOX4	AT1G72520	AY091193
LOX5	AT3G22400	NM_113137
LOX6	AT1G67560	NM_105423
AOS	AT5G42650	AY128755
AOC1	AT3G25760	NM_113475
AOC2	AT3G25770	NM_113476
AOC3	AT3G25780	NM_113477
AOC4	AT1G13280	NM_101199
CTS	AT4G39850	NM_001204043
OPR2	AT1G76690	NM_106319
OPR3	AT2G06050	NM_001084415
GH3.10	AT4G03400	NM_001340446
GH3.11/JAR1	AT2G46370	NM_180122
COI1	AT2G39940	NM_129552
JAZ1	AT1G19180	NM_001332386
JAZ2	AT1G74950	NM_10615
JAZ3	AT3G17860	NM_001084705
JAZ4	AT1G48500	NM_001123979
JAZ5	AT1G17380	NM_101599
JAZ6	AT1G72450	NM_105904
JAZ7	AT2G34600	NM_129014
JAZ8	AT1G30135	NM_102753
JAZ9	AT1G70700	NM_105738
JAZ10	AT5G13220	NM_203046
JAZ11	AT3G43440	NM_114212

JAZ12	AT5G20900	NM_122098
ILR1	AT3G02875	AY081499
ILL6	AT1G44350	AY074380
IAR3	AT1G51760	NM_104055
CYP94B3	AT3G48520	NM_114710
JAO1	AT3G11180	NM_111954
JAO2	AT5G05600	NM_120642
JAO3	AT3G55970	NM_115455
JAO4	AT2G38240	NM_129381
CYP20-3	AT3G62030	AY093284
JID1	AT1G06620	NM_100539
ZAT10	AT1G27730	AY063006
GRX480	AT1G28480	BT029336
VSP1	AT5G24780	AY044328
PDF1.2	AT5G44420	AY133787
THI2.1	AT1G72260	AY080781
GH3.1	AT2G14960	NM_127059
GH3.2	AT4G37390	NM_119902
GH3.3	AT2G23170	NM_127881
GH3.4	AT1G59500	NM_104643
GH3.5	AT4G27260	NM_118860
GH3.6	AT5G54510	NM_124831
GH3.7	AT1G23160	NM_102164
GH3.8	AT5G51470	NM_124526
GH3.9	AT2G47750	NM_130342
GH3.12	AT5G13320	NM_121335
GH3.13	AT5G13350	NM_001343270
GH3.14	AT5G13360	NM_001036797
GH3.15	AT5G13370	NM_121340
GH3.16	AT5G13380	NM_121341

GH3.17	AT1G28130	NM_179387
GH3.18	AT1G48670	NM_001333347
GH3.19	AT1G48660	NM_001333346
ILL2	AT5G56660	AY072084
