# Supplementary material for: Amide conjugates of the jasmonate precursor cis-(+)-12-oxo-phytodienoic acid regulate its homeostasis during plant stress responses
Source: Plant Physiol. 2024 Nov 28;197(1):kiae636. doi: 10.1093/plphys/kiae636 (PMC11663710; doi:10.1093/plphys/kiae636)
Supplement: kiae636_Supplementary_Data [file kiae636_supplementary_data.zip › Supplementary Note S1.pdf]

Supplementary Note S1

- Synthesis of deuterium labeled cis-OPDA amino acid conjugates – (+)-OPDA-d_5-Val, (+)-OPDA-d_5-Ile

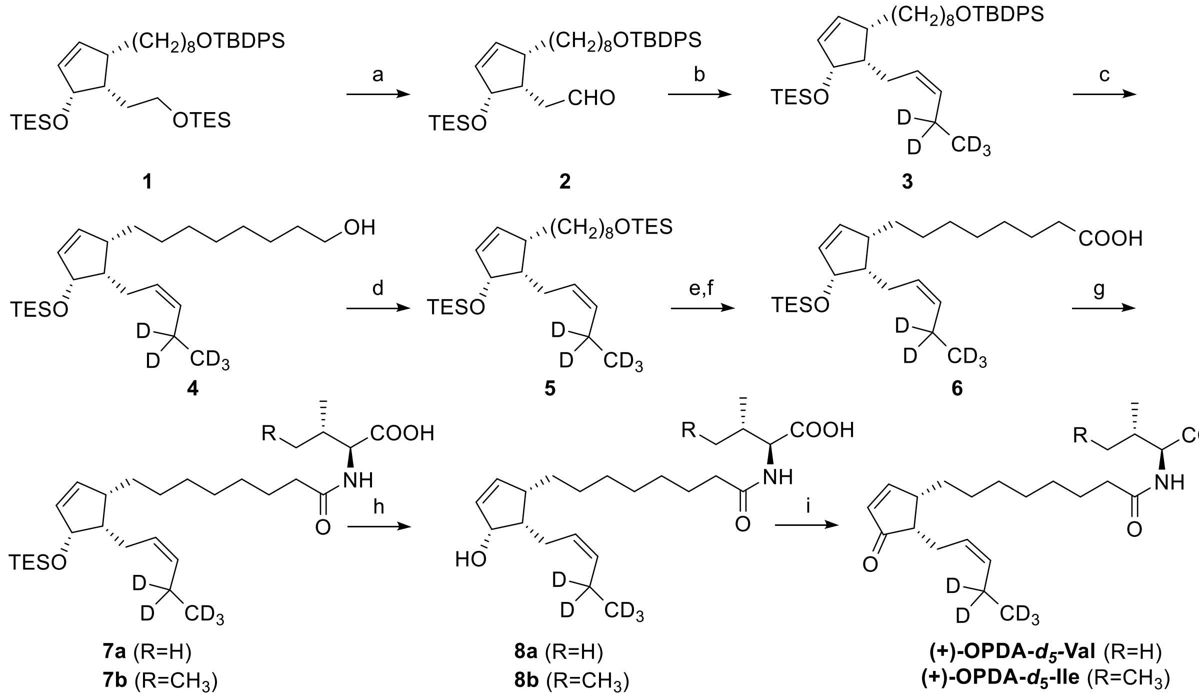

Scheme S1. Synthesis of (+)-OPDA-*d*$_5$-Val and (+)-OPDA-*d*$_5$-Ile. *Reagent and conditions*: (a) $(COCl)_2$, DMSO, CH_2Cl_2; Et_3N; (b) $[Ph_3PCH_2CD_2CD_3]^+Br^-$, NaHMDS, THF; (c) TBAF, THF, reflux; (d) TESCl, imidazole, DMF; (e) $(COCl)_2$, DMSO, CH_2Cl_2, Et_3N; (f) $NaClO_2$, NaH_2PO_4, 2-methyl-2-butene, *t*-BuOH, H_2O; (g) $ClCO_2Et$, Et_3N, THF, 0 °C; L-Val/L-Ile, NaOH, H_2O; (h) TBAF, THF; (i) Jones reagent, acetone, -20 ℃.

To a solution of DMSO (1.1 ml, 15.5 mmol) in CH_2Cl_2 (9.7 ml) was added oxalyl chloride (0.58 ml, 6.71 mmol) at -78 °C under an argon atmosphere. After the reaction mixture was stirred at -78 °C for 10 min, a solution of **1** (Nonaka et al., 2010) (961 mg, 1.32 mmol) in CH_2Cl_2 (11 ml) was slowly added. After the reaction mixture was stirred at -65 ℃ for 1 h, Et_3N (2.0 mL, 14.3 mmol) was slowly added and stirred at -65 ℃ for 3 h. Then the reaction mixture was gradually warmed to room temperature. The reaction was quenched with saturated aq. NH_4Cl. The mixture was extracted with *n*-hexane. The organic layer was washed with brine, dried over Na_2SO_4, and concentrated under reduced pressure to afford crude aldehyde **2** (948 mg, slightly impure) as a pale yellow oil. The crude product was used for the following reaction without further purification.

To an ice-cold suspension of $[Ph_3PCH_2CD_2CD_3]^+Br^-$ (948 mg, 4.47 mmol) in THF (16.8 ml) was added NaHMDS (2.35 ml, 1.9 M in THF, 4.47 mmol). The resulting orange-red mixture was stirred at room temperature for 40 min and cooled to -78 °C. To this solution was added a solution of the above aldehyde **2** in THF (16.5 ml) dropwise. The resulting solution was stirred at -78 °C for 2 h and added DMF (2.5 ml),

then at room temperature for 2 h, quenched with saturated aq. NH_4Cl, extracted with *n*-hexane. The combined organic layer was dried over Na_2SO_4 and concentrated under reduced pressure. The residue was purified by silica gel medium-pressure chromatography (eluent: 99:1 *n*-hexane/EtOAc to 92:8 *n*-hexane/EtOAc) to give **3** (722 mg, slightly impure) as colorless oil and was used for the following reaction without further purification.

To a solution of **3** (722 mg, slightly impure) in THF (161 ml) was added TBAF (19 ml, 1.0 M in THF, 19 mmol). The solution was heated under reflux for 2 h. After being cooled to room temperature, the solvent was removed under reduced pressure. The residue was purified by silica gel medium-pressure chromatography (eluent: 90:10 *n*-hexane/EtOAc to *n*-hexane/EtOAc = 88:12 to EtOAc) to give **4** (293 mg, slightly impure) as colorless oil and was used for the following reaction without further purification.

To a solution of **4** (130 mg, slightly impure) and imidazole (219 mg, 3.21 mmol) in DMF (3.0 ml) was added TESCl (232 µl, 1.38 mmol). The mixture was stirred for 1 h and was diluted with H_2O with vigorous stirring. The aqueous layer was extracted with *n*-hexane. The combined organic layer was washed with brine, dried over Na_2SO_4, and concentrated under reduced pressure. The residue was purified by silica gel medium-pressure chromatography (eluent: *n*-hexane to *n*-hexane/EtOAc = 94:6) to give **5** (126 mg, 42% in 4 steps) as a colorless oil.

To a solution of DMSO (184 µl, 2.58 mmol) in CH_2Cl_2 (1.8 ml) was added oxalyl chloride (103 µl, 1.19 mmol) at -78 °C under an argon atmosphere. After the reaction mixture was stirred at -78 °C for 20 min, a solution of **5** (126 mg, 245 µmol) in CH_2Cl_2 (2.2 ml) was slowly added. After stirring the reaction mixture at -65 °C for 1 h, Et_3N (368 µl, 2.62 mmol) was slowly added. The mixture was gradually warmed to room temperature for 95 min with stirring. The reaction mixture was quenched with saturated aq. NH_4Cl. The mixture was extracted with *n*-hexane. The organic layer was washed with brine, dried over Na_2SO_4, and concentrated under reduced pressure to afford crude aldehyde (107 mg) as pale yellow oil. The crude product was used for the following reaction without further purification. To a solution of the above aldehyde and 2-methyl-2-butene (5.15 ml, 48.5 mmol) in *t*-BuOH (15.6 ml) were added H_2O (3.6 ml), $NaH_2PO_4{\cdot}2H_2O$ (1.35 g, 8.62 mmol) and $NaClO_2$ (325 mg, 35.9 mmol) and the mixture was stirred at room temperature for 95 min. The reaction was quenched with saturated aq. NH_4Cl. The aqueous layer was extracted with EtOAc, and the combined organic layer was dried over Na_2SO_4 and concentrated under reduced pressure. The residue was purified by silica gel medium-pressure chromatography (eluent: AcOH/*n*-hexane/EtOAc = 0.1:98:2 to AcOH/*n*-hexane/EtOAc = 0.1:80:20) to give **6** (62.9 mg, 62 % in 2 steps) as pale yellow oil.

To a solution of **6** in THF (0.17 M) was added Et_3N (2.8 eq.) followed by ethyl chloroformate (2.4 eq.) at 0 °C. After being stirred at 0 °C for 10 min, L-Val/L-Ile (2.4 eq.) dissolved in 0.3 M aq. NaOH (0.42 M) was added and stirred for 1 h at room temperature. The reaction mixture was then acidified with 1 M aq. HCl and extracted with EtOAc. The combined organic layer was washed with brine, dried over Na_2SO_4, and concentrated under reduced pressure. The residue was purified by silica gel medium-pressure chromatography (eluent: AcOH/$CHCl_3$ = 0.1:100 to AcOH/$CHCl_3$/MeOH = 0.1:94:6) to give **7a** (86%) and (80:20 *n*-hexane/EtOAc to EtOAc) to give **7b** (slightly impure) both as colorless oils.

To a solution of **7a**/**7b** in THF (0.16 M) was added 1 M TBAF in THF (3.1 eq.). After stirring at room temperature for 2.5 h, the solvent was concentrated under reduced pressure. The reaction mixture was quenched with 1 M aq. HCl, and the resulting mixture was extracted with EtOAc. The combined organic layer was washed with brine, dried over Na_2SO_4, and concentrated under reduced pressure. The residue was purified by silica gel medium-pressure chromatography (eluent: AcOH/$CHCl_3$/MeOH = 0.1:99:1 to AcOH/$CHCl_3$/MeOH = 0.1:90:10) to give **8a** (quant.) and **8b** both as colorless oils.

To a solution of **8a**/**8b** in acetone (0.01 M) was added Jones reagent (4.0 M solution) at -20 °C until the orange color of the reagent persisted. After 10 min of stirring at -20 °C, *i*-PrOH was added to quench the remaining reagent. Then, EtOAc or EtOAc/*n*-hexane (1:1, in case of **8b**) and H_2O were added, and the aqueous layer was extracted again with EtOAc. The combined organic layer was washed with brine, dried over Na_2SO_4, and concentrated under reduced pressure. The residue was purified by silica gel medium-pressure chromatography (eluent: AcOH/$CHCl_3$/MeOH = 0.1:99:1 to AcOH/$CHCl_3$/MeOH = 0.1:90:10) to give **(+)-OPDA-*d*$_5$-Val** (80%) and **(+)-OPDA-*d*$_5$-Ile** (99%) as colorless oil.

(+)-OPDA-*d*$_5$-Val - yield 80%, colorless oil. $[\alpha]_D^{20}$+91.93 (c 1.10, $CHCl_3$). ^1H NMR (400 MHz, $CDCl_3$) δ (ppm): 7.74 (dd, *J* = 6.0, 2.8 Hz, 1H), 6.26-6.20 (bs, 1H), 6.18 (dd, *J* = 5.8, 1.8 Hz, 1H), 5.45-5.31 (m, 2H), 4.44 (dd, *J* = 8.2, 5.0 Hz, 1H), 3.03-2.93 (m, 1H), 2.52 (dt, *J* = 14.8, 4.6 Hz, 1H), 2.44 (ddd, *J* = 10.1, 5.9, 4.3 Hz, 1H), 2.24 (t, *J* = 7.0 Hz, 2H), 2.18-2.08 (m, 1H), 1.81-1.57 (m, 3H), 1.53-1.08 (m, 9H), 0.97 (d, *J* = 6.8 Hz, 3H), 0.94 (d, *J* = 7.0 Hz, 3H). ^{13}C NMR (100 MHz, $CDCl_3$) δ (ppm): 211.1, 175.5, 173.5, 167.3, 132.9, 132.4, 127.0, 57.9, 49.8, 44.4, 36.7, 31.1, 30.8, 29.7, 29.3, 29.2, 27.6, 25.8, 23.8, 20.1-19.7 (m), 19.3, 17.8, 13.4-12.5 (m). HRMS (ESI) m/z: $[M-H]^-$ Calcd for $C_{23}H_{31}D_5NO_4$ 395.2963; Found 395.2956.

(+)-OPDA-*d*$_5$-Ile – yield 99%, colorless oil. $[\alpha]_D^{25}$ +129.2 (*c* 0.23, $CHCl_3$). ^1H NMR (400 MHz, $CDCl_3$) δ (ppm): 7.75 (dd, *J* = 5.8, 2.8 Hz, 1H), 6.20 (dd, *J* = 5.8, 2.0 Hz, 1H), 6.04-5.95 (m, 1H), 5.42 (d, *J* = 11.2 Hz, 1H), 5.36 (ddd, *J* = 11.6, 6.5, 4.7 Hz, 1H), 4.61 (dd, *J* = 8.2, 5.5 Hz, 1H), 3.04-2.94 (m, 1H), 2.52 (dtd, *J* = 15.2, 5.4, 1.7 Hz, 1H), 2.45 (ddd, *J* = 9.5, 6.0, 4.7 Hz, 1H), 2.25 (t, *J* =7.4 Hz, 2H), 2.18 (ddd, *J* = 15.9, 9.5, 6.5 Hz, 1H), 2.02-1.90 (m, 1H), 1.78-1.09 (m, 14H), 0.96 (d, *J* =6.4 Hz, 3H), 0.94 (t, *J* =7.6 Hz, 3H). ^{13}C NMR (100 MHz, $CDCl_3$) δ (ppm): 211.5, 175.1, 173.8, 167.7, 133.0, 132.4, 126.9, 56.5, 49.9, 44.3, 37.6, 36.6, 30.7, 29.6, 29.2, 29.1, 27.5, 25.6, 25.1, 23.7, 19.9 (quintet, $J_{C\text{-}D}$ = 19.7 Hz), 15.5, 13.0 (septet, $J_{C\text{-}D}$ = 19.1 Hz), 11.6. IR (neat): 3329, 2929, 1701, 1209 cm^{-1}. HRMS (ESI) m/z: $[M-H]^-$ Calcd for $C_{24}H_{33}D_5NO_4$ 409.3120; Found 409.3110.

(+)-OPDA-*d*$_5$-Val ^{1}H and ^{13}C NMR spectra

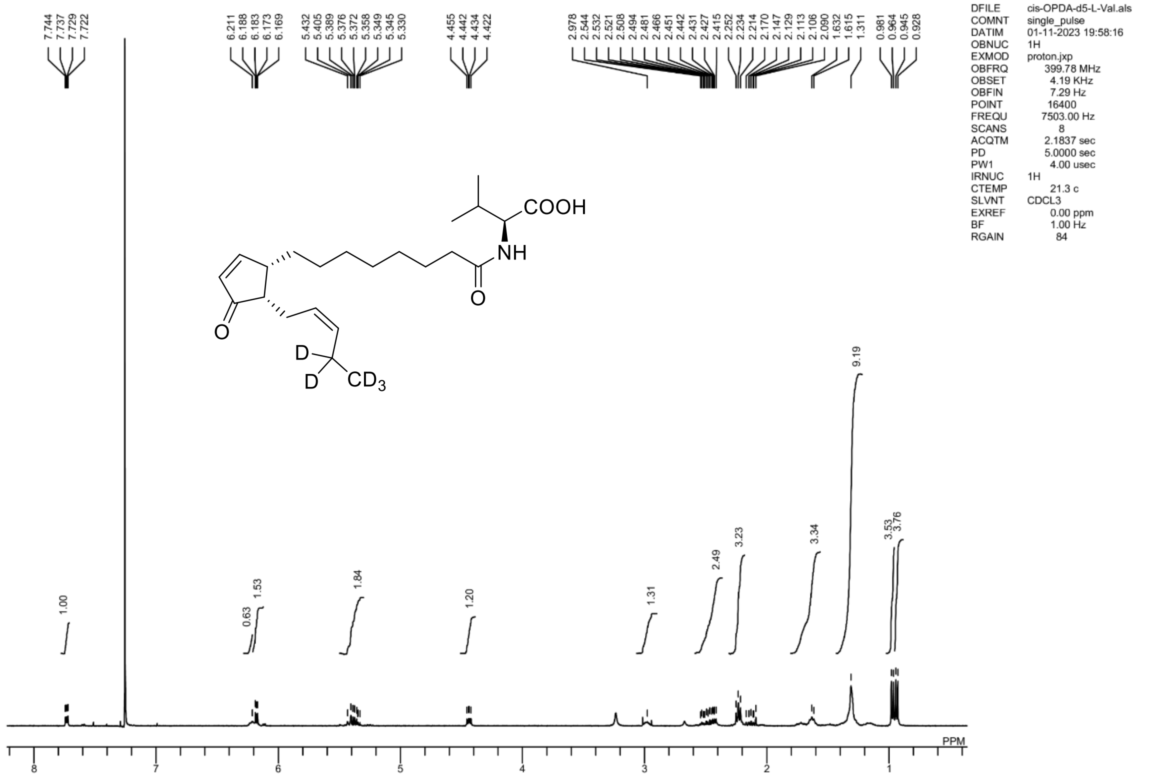

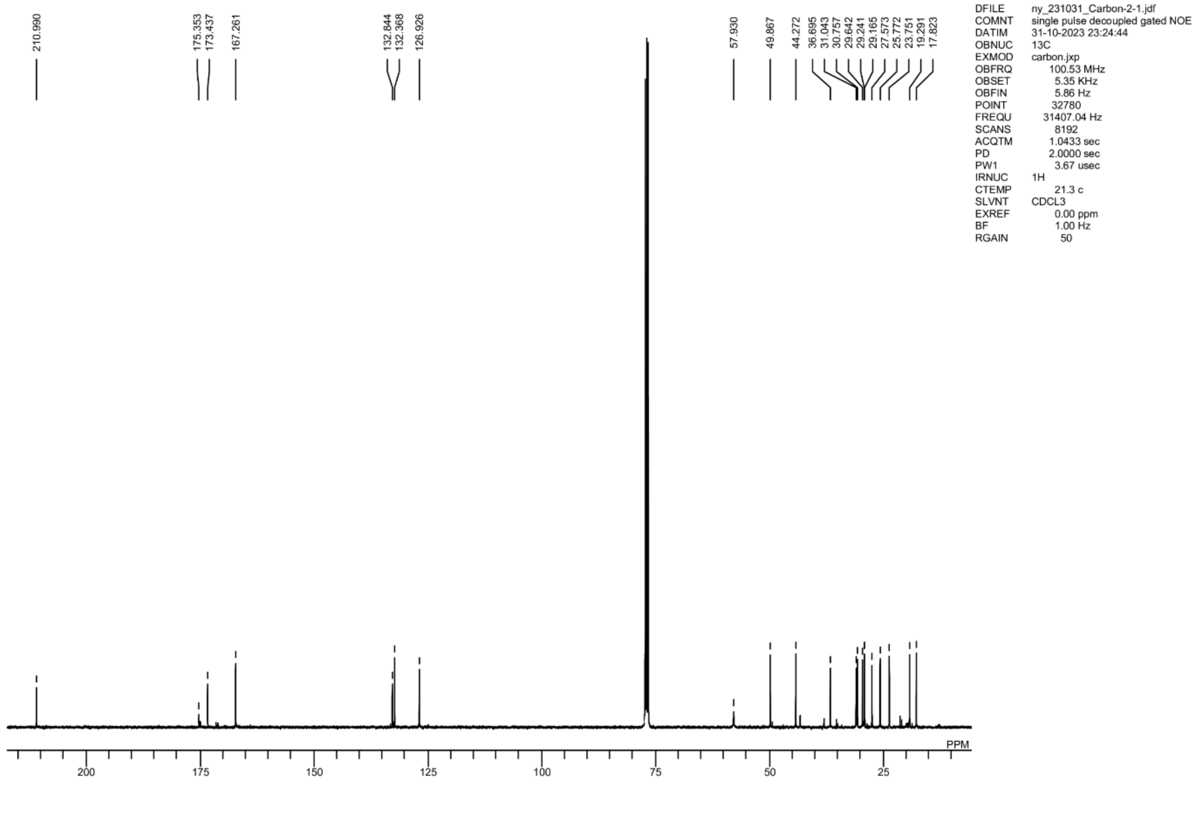

(+)-OPDA-*d*$_5$-Val ^{1}H and ^{13}C NMR spectra

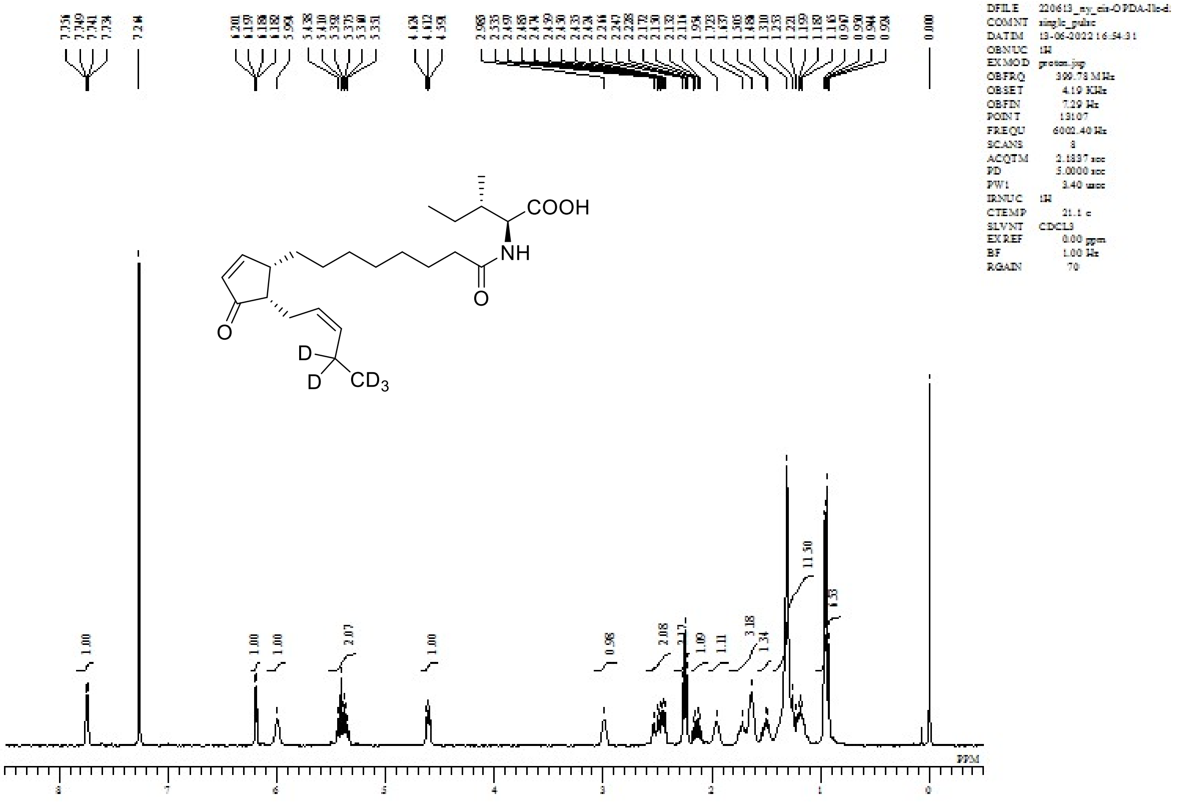

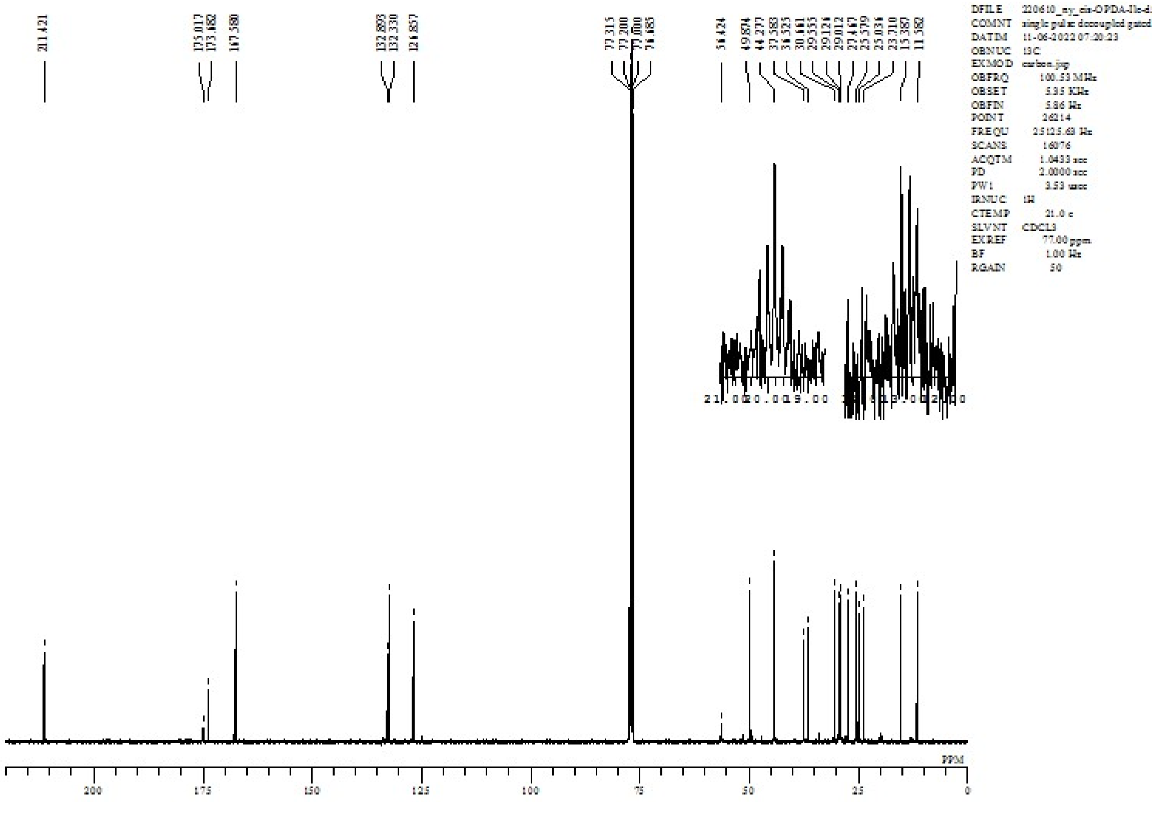

- Phytohormone measurements

Chemicals. Authentic and stable isotope labeled analytical standards **used for quantification** were purchased from OlChemIm Ltd. (Olomouc, Czech Republic): (±)-OPDA, (±)-JA, (-)-JA-Val, (-)-JA-Ile, (-)-JA-Phe, (±)-OPDA-d_5, (±)-JA-d_6, (-)-JA-d_2-Ile, IAA-$^{13}C_6$; IAA was purchased from Sigma Aldrich (St. Louis, MO, USA); (±)-JA-Ala, (±)-JA-Asp, (±)-JA-Gly, (±)-JA-Glu, (-)-JA-Met and (±)-JA-Trp were synthetised as described by Kramell et al. (1988); (±)-OPDA-Ala, (±)-OPDA-Asp, (±)-OPDA-Glu, (±)-OPDA-Ile, (±)-OPDA-Phe, (±)-OPDA-Trp, (±)-OPDA-Val and (±)-OPDA-d_5-Ile were synthetized following Mik et al. (2023). All the chemicals used for sample preparation and analysis were of analytical grade or higher.

Sample preparation. The samples from bacterial enzyme assays (1 ml) were centrifuged (20 000 *g*, 10 min, 8 °C), 2 µl of the supernatant and 10 µl of internal standard (IS) mixture (10 pmol of (±)-JA-d_6, 5 pmol of (-)-JA-d_2-Ile, (±)-OPDA-d_5, (±)-OPDA-d_5-Ile and 5 pmols IAA-$^{13}C_6$) were added into 28 µl of 20% aqueous acetonitrile. The samples were mixed with a pipette tip and analyzed by a liquid chromatography-tandem mass spectrometry system (LC-MS/MS).
The frozen plant material was powdered under liquid nitrogen with mortar and pestle and weighed in samples of 10 mg FW into 2 ml Eppendorf tubes. To each sample, 1 ml of 50% aqueous methanol, a mixture of IS (10 pmols of (±)-JA-d_6, 5 pmols (-)-JA-d_2-Ile, (±)-OPDA-d_5 and (±)-OPDA-d_5-Ile) and 4 ceria-stabilized zirconium oxide 2 mm beads (Retsch GmbH, Haan, Germany) were added. In feeding experiments with (+)-OPDA-d_5, (+)-OPDA-d_5-Ile and (+)-OPDA-d_5-Val 5 pmols of JA-d_2-Ile was added as IS. The samples were homogenized on a MM 400 mixer mill (Retsch GmbH, Haan, Germany) (27 Hz, 6 min, precooled holders) and centrifuged (25 800 *g*, 15 min, 4 °C). The supernatants were purified on solid phase extraction columns Oasis® HLB 1cc 30 mg columns (Waters, Milford, MA, USA) as described by Mik et al. (2023).

LC-MS/MS analysis. All samples were analyzed on an Agilent 6490 Triple Quadrupole LC/MS system coupled to a 1290 Infinity LC system (Agilent Technologies, Santa Clara, CA, USA) using chromatographic conditions and MS/MS parameters described by Široká et al. (2022) and Mik et al. (2023) or reported in **Supplemental Note Table 1**. The elution order of (+) and (-) JA-aa diastereomers was specified following Kramell et al. (1988) and Jikumaru et al. (2004). (±)-JA-Gly, (±)-JA-Glu, (±)-JA-Asp, (±)-JA-Trp, (±)-OPDA-Ala, (±)-OPDA-Asp, (±)-OPDA-Glu, (±)-OPDA-Ile, (±)-OPDA-Phe, (±)-OPDA-Trp and (±)-OPDA-Val were quantified as sum (Σ) of unresolved or partly resolved peaks. The JA-aa and OPDA-aa presence in the samples was identified based on the agreement of their RT and rates of confirmatory and reference MRMs with corresponding analytical standards. The separation of the compounds analyzed is depicted in the **Supplemental Note Figure 1.**

The MS system was operated in dynamic multiple reaction monitoring mode in positive and negative electrospray ionization mode. The nozzle voltage was set to 0 V, the capillary voltage to 2800/3000 V positive/negative mode, and the drying gas was at 130 °C with a flow rate of 14 l/min. The sheath gas was heated to 400 °C, and its flow rate was 12 l/min. The MassHunter Quantitative software package version B.09.00 (Agilent Technologies, Santa Clara, CA, USA) was used for data processing. The levels of the analytes were determined using a calibration curve prepared in the range of 0.0045 – 4.5 pmol for compounds in positive ionization and 0.0045 – 45 pmol for JA (negative ionization), logarithmically scaled. In feeding experiments with labelled compounds ((+)-OPDA-d_5, (+)-OPDA-d_5-Ile and (+)-OPDA-d_5-Val) the

levels of identified analytes were estimated semiquantitatively using peak areas and a calibration curve within the linear response.

Supplemental Note Table 1 LC-MS/MS method parameters.

Analyte	IS	Ionisation	Reference MRM (CE; eV)	Confirmatory MRM (CE; eV)	RT; min
(±)-OPDA	(±)-OPDA-d_5	$[M+H]^+$	293.2 > 275.2 (12)		13.7
(+)-OPDA-d_5		$[M+H]^+$	298.2 > 279.3 (12)		13.7
IAA	IAA-$^{13}C_6$	$[M+H]^+$	176.1 > 130.1 (24)		6.9
IAA-$^{13}C_6$ (IS)		$[M+H]^+$	182.1 > 136.0 (24)		6.9
(±)-JA	(±)-JA-d_6	$[M-H]^-$	209.2 > 58.8 (8)		10.1
(-)-JA-d_5*,#		$[M-H]^-$	214.2 > 58.8 (8)		10.1
(±)-JA-d_6 (IS)		$[M-H]^-$	215.2 > 58.8 (8)		10.1
(-)-JA-Ala/(+)-JA-Ala	(-)-JA-d_2-Ile	$[M+H]^+$	282.1 > 151.1 (12)	282.1 > 90.1 (16)	9.5/9.7
(±)-JA-Asp	(-)-JA-d_2-Ile	$[M+H]^+$	326.1 > 151.1 (14)	326.1 > 134.0 (16)	9.2
(±)-JA-Gly	(-)-JA-d_2-Ile	$[M+H]^+$	268.1. > 151.1 (12)	268.1 > 76.2 (16)	8.8
(±)-JA-Glu	(-)-JA-d_2-Ile	$[M+H]^+$	340.2 > 151.1 (20)	340.2 > 84.1 (40)	9.1
(-)-JA-Ile	(-)-JA-d_2-Ile	$[M+H]^+$	324.3 > 151.2 (16)	324.3 > 86.0 (26)	12.2
(-)-JA-d_5-Ile*,#		$[M+H]^+$	329.3 > 156.2 (16)	329.3 > 86.0 (26)	12.2
JA-d_2-Ile (IS)		$[M+H]^+$	326.3 > 151.2 (16)		12.2
(-)-JA-Met	(-)-JA-d_2-Ile	$[M+H]^+$	342.2 > 151.2 (20)	342.2 > 193.0 (10)	11.4
(-)-JA-Phe	(-)-JA-d_2-Ile	$[M+H]^+$	358.8 > 151.2 (16)	358.8 > 120.1 (30)	12.6
(±)-JA-Trp	(-)-JA-d_2-Ile	$[M+H]^+$	397.3 > 351.3 (12)	397.2 > 151.0 (18)	12.1
(-)-JA-Val	(-)-JA-d_2-Ile	$[M+H]^+$	310.3 > 151.3 (16)	310.2 > 72.1 (30)	11.4
(±)-OPDA-Ala	(±)-OPDA-d_5-Ile	$[M+H]^+$	364.3 > 275.1 (22)	364.3 > 90.0 (20)	13.4
(+)-OPDA-d_5-Ala*,#		$[M+H]^+$	369.3 > 280.0 (22)	369.3 > 90.0 (20)	13.4
(±)-OPDA-Asp	(±)-OPDA-d_5-Ile	$[M+H]^+$	408.3 > 275.3 (22)	408.3 > 134.1 (22)	13.3
(+)-OPDA-d_5-Asp*,#		$[M+H]^+$	413.3 > 280.0 (22)	413.3 > 134.1 (22)	13.3
(±)-OPDA-Glu	(±)-OPDA-d_5-Ile	$[M+H]^+$	422.3 > 275.3 (22)	422.3 > 148.1 (22)	13.0
(+)-OPDA-d_5-Glu*,#		$[M+H]^+$	427.2 > 280.0 (26)	427.2 > 148.1 (26)	13.0
(±)-OPDA-Ile	(±)-OPDA-d_5-Ile	$[M+H]^+$	406.2 > 86.1 (36)	406.2 > 275.1 (28)	14.4

(+)-OPDA-d_5-Ile*,#		$[M+H]^+$	411.2 > 86.1 (30)	411.2 > 280.0 (28)	14.4
(±)-OPDA-d_5-Ile (IS)		$[M+H]^+$	411.2 > 86.1 (30)	411.2 > 280.0 (28)	14.4
(±)-OPDA-Phe	(±)-OPDA-d_5-Ile	$[M+H]^+$	440.3 > 120.1 (40)	440.3 > 275.2 (30)	14.8
(+)-OPDA-d_5-Phe*,#		$[M+H]^+$	445.3 > 120.1 (32)	445.3 > 280.1 (30)	14.8
(±)-OPDA-Trp	(±)-OPDA-d_5-Ile	$[M+H]^+$	479.2 > 158.9 (28)	479.2 > 433.2 (22)	14.3
(±)-OPDA-Val	(±)-OPDA-d_5-Ile	$[M+H]^+$	392.3 > 72.2 (40)	392.3 > 275.1 (24)	14.1
(+)-OPDA-d_5-Val*,#		$[M+H]^+$	397.3 > 72.2 (20)	397.3 > 280.0 (24)	14.1

* The MRM were derived from fragmentation pattern and collision energies of not labeled compounds with respect to position of deuterated hydrogens on OPDA-d_5. Only used in feeding experiments with labeled compounds ((+)-OPDA-d_5, (+)-OPDA-d_5-Ile and (+)-OPDA-d_5-Val).

# The most probable chirality.

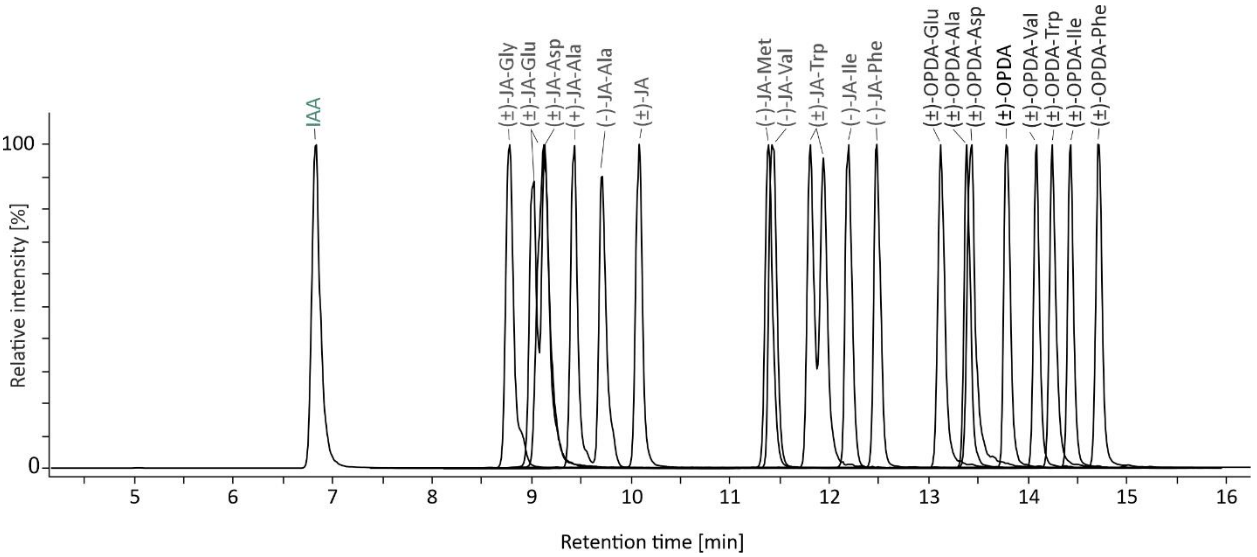

Supplemental Note Figure 1 Chromatographic separation of IAA, (±)-JA, (±)-OPDA, (±)-JA-aa and (±)-OPDA-aa.

Abbreviations used

AcOH	acetic acid
DMSO	dimethyl sulfoxide
DMF	*N*,*N*-dimethylformamide
EtOAc	ethyl acetate
HRMS	high-resolution mass spektrometry
IAA	2-(1*H*-indol-3-yl)acetic acid
IAA-$^{13}C_6$	2-(1*H*-indol-3-yl-3a,4,5,6,7,7a-$^{13}C_6$)acetic acid
(±)-JA	(±)-jasmonic acid

JA-Ala	jasmonoyl-*L*-alanine
	**Note:** other JA-amino acid conjugates analogically
JA-d_2-Ile	{2-[3-oxo-2-((*Z*)-pent-2-en-1-yl)cyclopentyl]acetyl-d_2-d_2}-*L*-isoleucine
JA-d_6	(±)-(*Z*)-2-(3-oxo-2-(pent-2-en-1-yl-3,4,4,5,5,5-d_6)cyclopentyl)acetic acid
NaHMDS	sodium hexamethyldisilazane
NMR	nuclear magnetic resonance
(±)-OPDA	8-[(1*S**,5*S**)-4-oxo-5-((*Z*)-pent-2-en-1-yl)cyclopent-2-en-1-y]octanoic acid
(±)-OPDA-Ala	{8-[(1*S**,5*S**)-4-oxo-5-((*Z*)-pent-2-en-1-yl)cyclopent-2-en-1-yl]octanoyl}-*L*-alanine
	**Note:** other (±)-OPDA - amino acid conjugates analogically
(±)-OPDA-d_5	8-[(1*S**,5*S**)-4-oxo-5-((*Z*)-pent-2-en-1-yl-4,4,5,5,5-d_5)cyclopent-2-en-1-y]octanoic acid
(±)-OPDA-d_5-Ala	{8-[(1*S**,5*S**)-4-oxo-5-((*Z*)-pent-2-en-1-yl-4,4,5,5,5-d_5)cyclopent-2-en-1-yl]octanoyl}-L-alanine
	**Note:** other (±)-OPDA-d_5 - amino acid conjugates analogically
(+)-OPDA-d_5	8-[(1*S*,5*S*)-4-oxo-5-((*Z*)-pent-2-en-1-yl-4,4,5,5,5-d_5)cyclopent-2-en-1-y]octanoic acid
(+)-OPDA-d_5-Ile	{8-[(1*S*,5*S*)-4-oxo-5-((*Z*)-pent-2-en-1-yl-4,4,5,5,5-d_5)cyclopent-2-en-1-yl]octanoyl}-L-isoleucine
(+)-OPDA-d_5-Val	{8-[(1*S*,5*S*)-4-oxo-5-((*Z*)-pent-2-en-1-yl-4,4,5,5,5-d_5)cyclopent-2-en-1-yl]octanoyl}-L-valine
TBAF	tetra-*n*-butylammonium fluoride
TBDPS	*tert*-butyldiphenylsilyl
t-BuOH	*tert*-butanol
TESCl	triethylsilyl chloride
THF	tetrahydrofuran

DESI-MSI

For DESI-MSI, wild-type Col-0 and *ill6-2* mutant Arabidopsis plants were grown in soil under long-day conditions (16 h light/8 h dark) in a cultivation chamber maintained at 21 °C, with a light intensity of approximately 170 µmol m^{-1} s^{-1} and 40-60% relative humidity. Wounding was conducted on rosette leaves of 3-week-old plants using a jaw tweezer press and wounded leaves were harvested together with unwounded control leaves 4 h after wounding. Sample leaves were freshly collected and the surface cuticle was removed by petroleum ether wash and the leaves were mounted on Superfrost glass slides (Thermo Fisher Scientific, Waltham, MA, USA) using non-conductive double-sided tape (Plano GmbH, Wetzlar, Germany). Sample slides were dried in a vacuum desiccator (Merck), scanned and subjected to DESI-MSI and DESI-MS/MSI analysis. DESI-MSI for *in situ* visualization of OPDA conjugates was performed using a Synapt G2-Si MS instrument coupled to a 2D-DESI source (Waters). The spray solvent (80% ACN (v/v) with 0.1% ammonia for imprints) was delivered at 2 µl/min and nebulized with 0.5 Mbps ultrapure nitrogen on sample slides. The DESI source was optimized as follows: tip-to-surface distance: 1 mm; tip-to-inlet distance: 5 mm; incidence angle: 55°; collection angle: 10°; capillary voltage: 4 kV; cone voltage: 30 eV; m/z range: 100–600, with 16 000–17 000 full width at half-maximum (FWHM) mass resolution. Data were acquired in the negative mode using MassLynx™ software (v4.1, Waters, Milford, MA, USA). Spectra, with a spatial resolution of 200 µm, were acquired every second. All acquired spectra were then re-calibrated with the exact mass of the standard compounds and normalized based on the total ion count (TIC) intensities. DESI-MS/MSI was performed on the consecutive samples with a 20-25 eV collision energy scanning of the wounded leaves and acquired MS/MS spectra were processed into peak lists, and the correct molecular formulas were first calculated using MassLynx™ software (v4.1) and then identified by their masses acquired from the standard solution. Afterwards, acquired MSI and MS/MSI data were imported into msIQuant 2.x (Uppsala University, Sweden) for the ion intensity maps generation.

Additional references

Jikumaru Y, Asami T, Seto H, Yoshida S, Yokoyama T, Obara N, Hasegawa M, Kodama O, Nishiyama M, Okada K, Nojiri H, Yamane H (2004) Preparation and biological activity of molecular probes to identify and analyze jasmonic acid-binding proteins. Bioscience Biotechnology and Biochemistry **68:** 1461-1466

Kramell R, Schmidt J, Schneider G, Sembdner G, Schreiber K (1988) Synthesis of N-(jasmonoyl) amino acid conjugates. Tetrahedron **44**: 5791-5807

Nonaka H, Ogawa N, Maeda N, Wang YG, Kobayashi Y (2010) Stereoselective synthesis of epi-jasmonic acid, tuberonic acid, and 12-oxo-PDA. Org Biomol Chem **8**: 5212-5223
