# Supplementary material for: Amide conjugates of the jasmonate precursor cis-(+)-12-oxo-phytodienoic acid regulate its homeostasis during plant stress responses
Source: Plant Physiol. 2024 Nov 28;197(1):kiae636. doi: 10.1093/plphys/kiae636 (PMC11663710; doi:10.1093/plphys/kiae636)
Supplement: kiae636_Supplementary_Data [file pp.02186.2024-s07.pdf › pp.02186.2024-s07.pdf]

Plant Physiology®

Homeostasis of cis-OPDA amide conjugates upon plant stress responses

Federica Brunoni, Jitka Široká, Anita Ament, Václav Mik, Tomáš Pospíšil, Michaela Kralova, Chao Zhang, Markéta Pernisová, Michal Karady, Vladimíra Nožková, Yuho Nishizato, Takuya Kaji, Rina Saito, Mohamed Htitich, Kristýna Floková, Claus Wasternack, Miroslav Strnad, Minoru Ueda, and Ondřej Novák

Review Timeline:

Submission Date:	28-Jun-2023
Editorial Decision:	07-Jul-2023
Resubmission Received:	01-Aug-2024
Editorial Decision:	20-Aug-2024
Revision Received:	02-Oct-2024
Editorial Decision:	18-Oct-2024
Revision Received:	01-Nov-2024
Accepted:	04-Nov-2024

07-Jul-2023

Dr. Federica Brunoni
Palacký University
Olomouc
Czech Republic

RE: Conjugation of cis-OPDA with amino acids is a conserved pathway affecting cis-OPDA homeostasis upon stress responses

Dear Dr. Federica Brunoni:

Thank you for giving us the opportunity to review your manuscript for publication in the Plant Physiology. The reviewers have found the manuscript of interest, but have raised a number of concerns that will require your attention. As these concerns are of some gravity and will entail substantial changes to the manuscript, you are likely to need additional time to address them. I must therefore reject the manuscript in its present form. Nonetheless, if you are able to address the points raised in review, I would welcome your resubmission of a manuscript that incorporates your responses.

In particular, I direct your attention to the following comments for consideration in a resubmitted manuscript.

Both reviewers appreciate the efforts conducted and the careful design of your experiments. Yet, they both agreed that the current manuscript remains fairly descriptive and therefore does not meet the standards of PP yet. One of the major concerns is that most of the research is conducted with exogenously applied OPDA(-conjugates). If you feel you can profoundly go more in to the action, formation and faith of endogenous OPDA-conjugates, then I would like to encourage you to resubmit. If you prefer not do so, as this will require extensive additional experimentation, I would like you not to consider resubmission.

Please note that any resubmission will be treated as a new entry to the system and is likely to go out for review.

If you do have any queries or concerns, you are welcome to contact me in the first instance.

Thank you again for allowing Plant Physiology to review your work. We look forward to hearing from you soon.

Sincerely,

Editor, Plant Physiology

-- Reviewer comments:
Reviewer #1 (Comments for the Author):

In this well-structured and well-written MS, the Authors focus on the analyses of OPDA-amino-acid molecules in plants, primary in the model plant Arabidopsis. The very limited knowledge on OPDA-Ile is greatly expanded to several OPDA-aa. Several biochemical, genetic, enzymatic and metabolic approaches are employed to describe the accumulation of the OPDA-aa with the aim to define the enzymes involved in the homeostasis of these molecules. The experiments are well designed and controls are included in most cases. Finally, the in vitro analysis of enzymes from different plant species as well as the study of mutant lines of Physcomitrium aim to study the conservation of the OPDA-aa: it is a very nice, although fairly limited, evolutionary approach.

Although the MS reads well, there are a number of points that should be addressed:

Major points:

1) Fig 3: What is the rationale to select OPDA-Val and OPDA-Glu as representatives of the 2 groups to design standards isotopically labeled on the amino acid moiety? OPDA-Val is quite active in inducing gene expression whereas OPDA-Glu is almost inactive in inducing this response (Fig 3A). Would it not be better to select a representative OPDA-aa more-active-than-OPDA-Glu?

2) Fig 4B: it is very clear that the sextuple gh3 mutant fails to accumulate OPDA-Asp after exogenous OPDA treatment. Since OPDA-Asp is highly induced after wounding, a natural/endogenous stress, it will be very interesting to analyze the biosynthesis of OPDA-Asp after wounding in the sextuple gh3 mutant to further support the conclusions.

3) Results shown in fig 5 suggest that AtILL6 plays a primary role in hydrolyzing several OPDA-aa. To support the putative role

of OPDA-aa as inactive pool of OPDA (to activate the canonical COI1-dependent responses), it may be good to analyses JA-responses (growth inhibition, gene expression, ect) in ill6 mutant and WT plants wound-stressed or/and treated with different OPDA and OPDA-aa. These simple experiments may be very informative.

4) Fig 6B: similar to the previous comments, to study the endogenous function of PpGH3 in the mutant lines, it'd be better to study the accumulation of OPDA-aa after wounding compared to exogenous OPDA treatment.

5) Fig 6B: what is the rationale to test the accumulation of OPDA-Asp in vivo? The in vitro assay suggest that OPDA-Ala is a likely product of the PpGH3 activity. Please add the analysis of OPDA-Ala in Physcomitrium WT and mutant lines.

6) Line 145 and generally in the text: please avoid the wording "lower land" referring to Physcomitrium, it is not an accepted scientific nomenclature and, I am afraid, it is simply not correct. Please refer to Physcomitrium as a Bryophyte, moss or non-vascular plant.

7) Are the markers described in line 213 "THI2.1, VSP1, ZAT10, GRX480, JAZ5 and PDF1.2" both cis-OPDA and JA-responsive? In its current format, it is difficult to understand if the expression of specific genes regulated by cis-OPDA or by JA, or they are generally regulated by both OPDA and JA.

8) A general concern is about the in vitro enzymatic assay: the analyses of enzymes with limited specificity, such as GH3 and ILR (accepting different substrates, including SA and IAA), suggest particular caution. Authors clearly state that the in vitro activity is a general proxy, and I appreciate that in vivo experiments always support their conclusions.

9) Were the Ppgh3 ko mutants described before? Please add the reference/s. In case these mutants were generated by the Authors, it is important to characterize these mutants: how were they generated (CRISPR-Cas, homologous recombination, T-DNA ect)? How were they confirmed as complete loss-of-function?

10) Line 347 "These results confirm that cis-OPDA amino acid conjugation is an evolutionarily conserved metabolic pathway in plants" this conclusion is an overinterpretation. Based on these results, it'd be wise to suggest a conclusion similar to: cis-OPDA amino acid conjugation is a conserved metabolic pathway in 2 evolutionary "distant" land plants, the bryophyte Physcomitrium and the angiosperm P. abies.

Medium issues:

11) Line 98 "JA-Ile is turned over by two inducible and intertwined catabolic pathways. One is oxidative and mediated by cytochrome P450 enzymes of the subfamily 94 (CYP94)" indeed, the oxidation of JA-Ile to JA-Ile-OH was initially suggested to be a catabolic pathway (Koo et al., 2011). However, it has been recently showed that JA-Ile-OH can induce COI1-JAZ interaction, though with a lower affinity compared that of JA-Ile (Heitz et al., 2012; Koo et al., 2014; Jimenez-Aleman et al., 2019; Poudel et al., 2019).

12) The reactive electrophilic activity of dn-OPDA and OPDA is not clearly introduced. It is probably important to briefly describe the RES activity of these molecules.

13) Fig 2C: the staining of JAZ9-GUS in mock is very limited. It'd be good to add new images in which basal JAZ9-GUS staining is clear, or maybe it'd be better to remove the data from 35S:JAZ9-GUS analyses in its present format, which in inconclusive. As alternative, the GUS enzymatic activity may be quantified, although this is probably beyond the scope of this manuscript.

14) Fig 3: is it possible to labeled the OPDA moiety (instead of the aa moiety) of the ({plus minus})-OPDA-aa? It could be used to define more clearly the conclusions proposed.

15) What is the rationale to test the GH3 proteins of Group II of P. abies? Why were other GH3 proteins discarded?

16) Fig 5: "Considering the low hydrolytic activity of recombinant AtILL6 against OPDA-Ala and OPDA-Ile, it was surprising that these two OPDA-aa accumulated in the ill6-2 mutant" in line with the previous comment, a stronger correlation between in vitro activity on OPDA and in vivo OPDA-aa biosynthesis may be obtained in OPDA-feed plants compared to wounded plants. May it be worth to assess OPDA-aa accumulation in WT and hydrolase mutants after exogenous OPDA treatment?

17) Fig 6B: what is the rationale to generate the Ppgh3 double mutant? The analysis of the single gh3.2 ko already show that GH3.2 has a primary role in OPDA-aa synthesis in vivo, isn't it?

18) Discussion, line 363 "We further demonstrated that these ({plus minus})-OPDA-aa possess cis-OPDA-like activity on typical transcriptional and physiological cis-OPDA- and JA-responses in Arabidopsis (Figure 2; Figure 3A)" these conclusions are not supported by the results. Data of Figure 3A show that most OPDA-aa fail to induce the expression of most JA-/OPDA-marker genes. Please take into consideration the results shown in Fig 3A and rephrase the sentence.

Minor points:

19) Fig 3B: it'd be good to add "in medium" and "in plant" in the sub-panel to help the readers.

20) AtGH3.1 to .6 are clearly involved in the hydrolysis of OPDA-Asp (Fig 4): it'd be good to test gene expression and root growth inhibition in WT vs gh3 sextuple mutant in response to OPDA-Asp. Did you perform these easy but informative experiments?

21) My curiosity: do algae accumulate OPDA-aas?

Reviewer #2 (Comments for the Author):

This study builds on the authors' prior work showing that OPDA-Ile and other OPDA-amino acid conjugates (OPDA-AAs) are naturally occurring compounds in Arabidopsis. Given current interest in OPDA signaling and the activity of JA-Ile, the topic is of general interest. Here, the authors report data to support the claim that OPDA is conjugated to AAs under stress conditions to stimulate OPDA formation. Some new information on the metabolism of OPDA-AAs by GH3 conjugases and ILR hydrolases is reported. The biological role of OPDA-AA conjugates remains unclear. Several suggestions are offered to improve the work and its presentation.

Main points

This lab recently reported (Mik et al 2023) that OPDA-Ile levels are induced in wounded Arabidopsis leaves. But in the present study they say that OPDA-Ile cannot be detected. This discrepancy needs to be resolved, and calls into doubt the premise that OPDA-Ile or other OPDA-AAs are biologically relevant stress signals as previously suggested.

A shortcoming of the work is its focus on the action and metabolism of exogenous OPDA-AAs. Although this line of investigation is important for establishing what enzymes have the capacity to metabolize OPDA-AAs, it does not prove how endogenous pools of OPDA-AAs are metabolized or what the endogenous compounds might be doing.

The simplest interpretation of data in Fig 2 is that exogenous JA, OPDA and OPDA-aa are all converted by JAR1 to JA-Ile in planta. If this is true, it is uninformative with regard to the role (if any) of endogenous OPDA-AAs.

The authors provide some evidence that several GH3s/ILRs are involved in metabolizing exogenous OPDA. But the data is not definitive and raises many questions. Rigorous proof requires analysis of endogenous ODPA-AA levels in gh3/ilr mutants. The extreme low levels (10 to 100-fold less than JA-Ile) of OPDA-AAs in Arabidopsis leaves makes it challenging to address this question and also calls into question the biological relevance of these compounds.

A second shortcoming of the work is the lack of any new information about the physiological function of OPDA-AAs. A simple hypothesis is that most stress-induced production of OPDA in Arabidopsis enters the peroxisome (for JA biosynthesis) or gets conjugated into Arabidopsides. A small amount of this OPDA, however, may "leak" into the cytosol where it is toxic because of the high reactivity of the compound. Conjugation to AAs may reflect a mechanism to neutralize this toxicity. It might be interesting to look at the role of cytosolic OPR2 in OPDA/OPDA-AA metabolism.

Other points

Rather than stating the effects of OPDA-aa are mediated in a JA-Ile-dependent manner, it is more accurate to say that the effects occur in a JAR1-dependent manner.

The Introduction should mention that cis-OPDA is a highly reactive molecule owing to the unsaturated carbonyl group.

Figure legends should state the concentration of OPDA-AA used.

As a matter of writing style: breaking up the text into smaller paragraphs, each focused on a specific point that is delineated in the first sentence of the paragraph, would make for greater ease of reading and comprehension.

In the Introduction, references cited in support of previous research are generally okay but a few omissions were noted, as indicated below for the authors' consideration.

Dear Editors of Plant Physiology,

We would like to submit the research article entitled "Homeostasis of cis-OPDA amide conjugates upon stress responses" for publication. A previous version of this manuscript was submitted last June to Plant Physiology (PP2023-RA-00960) and evaluated for rejection, with resubmission encouraged. In this new version, we included additional evidence supporting the natural occurrence of synthesis and hydrolysis of OPDA amino acid conjugates (OPDA-aa) upon stress. We addressed most of the concerns raised by the reviewers and exclusively focused on OPDA-aa occurrence in the model species Arabidopsis. Furthermore, a complementary study carried out in the liverwort Marchantia polymorpha from Andrea Chini and Roberto Solano's group is currently being prepared and will be submitted to Plant Physiology. Therefore, we would like these two manuscripts to be considered co-submissions and back-to-back publications if they were both accepted.

Highlights reported in the new version are provided below in a point-by-point response to the Editor's and reviewers' comments.

Sincerely,

Federica Brunoni

>>>

Date: 07-Jul-2023 07:34:03
Last Sent: 07-Jul-2023 07:34:03
Triggered By: Redacted
CC: Redacted
BCC: Redacted
Subject: PP2023-RA-00960: Decision Letter
Message: 07-Jul-2023

Dr. Federica Brunoni
Palacký University
Olomouc
Czech Republic

RE: Conjugation of cis-OPDA with amino acids is a conserved pathway affecting cis-OPDA homeostasis upon stress responses

Dear Dr. Federica Brunoni:

Thank you for giving us the opportunity to review your manuscript for publication in the Plant Physiology. The reviewers have found the manuscript of

interest, but have raised a number of concerns that will require your attention. As these concerns are of some gravity and will entail substantial changes to the manuscript, you are likely to need additional time to address them. I must therefore reject the manuscript in its present form. Nonetheless, if you are able to address the points raised in review, I would welcome your resubmission of a manuscript that incorporates your responses.

In particular, I direct your attention to the following comments for consideration in a resubmitted manuscript.

Both reviewers appreciate the efforts conducted and the careful design of your experiments. Yet, they both agreed that the current manuscript remains fairly descriptive and therefore does not meet the standards of PP yet. One of the major concerns is that most of the research is conducted with exogenously applied OPDA(-conjugates). If you feel you can profoundly go more in to the action, formation and faith of endogenous OPDA-conjugates, then I would like to encourage you to resubmit. If you prefer not do so, as this will require extensive additional experimentation, I would like you not to consider resubmission.

Please note that any resubmission will be treated as a new entry to the system and is likely to go out for review.

If you do have any queries or concerns, you are welcome to contact me in the first instance.

Thank you again for allowing Plant Physiology to review your work. We look forward to hearing from you soon.

Sincerely,

Editor, Plant Physiology

> First, we express our gratitude to the editor and reviewers for carefully evaluating our work and their valuable comments, which were deeply taken into account when preparing the revision of this manuscript. The major issue when studying the endogenous occurrence of OPDA-aa concerns the low abundance of these compounds that are not detected under steady-state growth conditions but only upon stress. We addressed this issue by testing the effect of different types of stress (biotic and abiotic and by exogenous application of OPDA and OPDA-aa). We observed that all these stress stimuli elicited the formation of OPDA-aa, highlighting that the amino acid conjugation of OPDA is likely a general, rather than a specific, plant response to stress. Since OPDA-aa were formed at a much higher level upon exogenous application of OPDA than in response to biotic and abiotic stress, we took advantage of this strong plant response to examine whether OPDA-aa retained any cis-OPDA-like activity and identify the enzymes responsible for their synthesis and hydrolysis.

*> We observed that OPDA-aa induced root-growth inhibition and that this effect required functional JA-Ile formation, and provided in this revised manuscript evidence that the first identified OPDA-aa OPDA-Ile is not a ligand for functional COI1-JAZ co-receptor (Fig. 3B). These results suggested that OPDA-aa are metabolized in planta. We further confirmed the hydrolysis of OPDA-aa by in planta-feeding assay with stable isotope labeled OPDA-aa. We performed these feeding experiments with deuterated (d_5) OPDA-Val and OPDA-Ile labeled on the OPDA moiety in this revised manuscript. These new standards allowed us to follow the fate of the OPDA-aa by monitoring the formation of d_5-derivatives over time (Fig. 3C and D; Supplemental Figure S2) and not only to assess their hydrolysis. Results from feeding experiments confirmed OPDA-Val and –Ile are cleaved into cis-OPDA and converted to OPC-4, JA, and JA-Ile by plants already 30 min after application and revealed that **not all the cis-OPDA deriving from the OPDA-aa hydrolysis is channeled into the JA biosynthesis**, as it can be re-conjugated with Glu, Asp, and Ile. A schematic representation of the in planta metabolic conversion of radiolabeled OPDA-Val and OPDA-Ile is provided in Supplemental Figure S2C.*

In the second part of this study, we showed that the synthesis and hydrolysis of cis-OPDA amino acid conjugates are mediated by members of the amidosynthetase GH3 and the amidohydrolase ILR1/ILL families. The identification of the metabolic enzymes responsible for OPDA-aa synthesis and hydrolysis allowed us to demonstrate that OPDA-aa are rapidly and endogenously accumulated after wounding in amidohydrolase mutants and amidohydrolases, whose genes are upregulated upon wounding (Widemann et al., 2013; Zhang et al., 2016), are responsible for the rapid hydrolysis of OPDA-aa (Fig. 5B). In this revised manuscript, we additionally showed that the identified cis-OPDA-conjugating GH3s redundantly contribute to the formation of OPDA-aa upon wounding (Fig. 4C).

*Collectively, this evidence demonstrates that oppositely to JA, and similarly to IAA, amino acid conjugation of cis-OPDA is not required for plant hormone activation, **OPDA-aa function in the catabolism or temporary storage of cis-OPDA in stress responses** instead of acting as chemical signals per se.*

Considering that OPDA-aa are endogenous low abundant cis-OPDA derivatives only formed upon stress and rapidly hydrolyzed back to cis-OPDA in response to wounding, in this revised manuscript, we investigated whether the formation of OPDA-aa might be localized at the damaged site of the leaf. We showed that OPDA-aa equally accumulated in the wounded and unwounded area of damaged leaves in Col-0 and ill6 loss-of-function mutant, demonstrating that the low abundance of OPDA-aa is not a consequence of a tissue-specific OPDA-aa formation (Fig. 6A). Interestingly, upon wounding, cis-OPDA accumulated predominantly at the damaged site in Col-0 leaves. In contrast, the cis-OPDA distribution was widespread all over the leaf in the ill6-2 mutant, albeit the leaf damage induced an increase of cis-OPDA level in both genotypes. This suggests that ILL6 is required to channel cis-OPDA at the damaged site in response to wounding.

Endogenous conjugation of cis-OPDA (and deconjugation of OPDA-aa) implies that not all the cis-OPDA is metabolized in the peroxisome and that plants have evolved mechanisms to regulate its homeostasis through transport (Schulze et al., 2019) and metabolism (Floková et al., 2016; Mik et al., 2023; this study), similarly to other important phytohormones like JA and IAA. Part of the chloroplast-derived cis-OPDA accesses the cytosol, perhaps through the postulated "leak" pathway (Theodoulou et al., 2005). We included new proofs about the subcellular localization of GH3 enzymes, demonstrating for the first time a dual cytosolic and nuclear localization of Arabidopsis GH3.3 protein (Fig. 4D and E) that points to cytoplasm and nucleus as potential sites for GH3 activity. This new evidence, together with the previously described ER localization of Arabidopsis ILR1/ILL enzymes (Zhang et al., 2016; Sanchez Carranza et al., 2016), confirm ***a homeostatic regulation of the cytosolic cis-OPDA pool in the ER*** *and prompted us to propose a model for cis-OPDA and OPDA-aa subcellular homeostasis (Fig. 6B). Once synthesized in the chloroplast, cis-OPDA is mainly reduced and β-oxidized to produce JA in the peroxisome. Part of the chloroplast-derived cis-OPDA reaches the cytosol, where GH3 enzymes conjugate it with amino acids to form OPDA amino acid conjugates (OPDA-aa). OPDA amide conjugates are hydrolyzed back to cis-OPDA in the endoplasmic reticulum (ER) by ILR1/ILL enzymes, and cis-OPDA is then released in the nucleus. Nuclear cis-OPDA can activate the expression of cis-OPDA-responsive genes or be conjugated with amino acids by GH3s. According to this model,* ***cis-OPDA amino acid conjugation might be important in regulating nuclear cis-OPDA levels through the ER****.*

References:

- Widemann, E., Miesch, L., Lugan, R., Holder, E., Heinrich, C., Aubert, Y., ... & Heitz, T. (2013). The amidohydrolases IAR3 and ILL6 contribute to jasmonoyl-isoleucine hormone turnover and generate 12-hydroxyjasmonic acid upon wounding in Arabidopsis leaves. *Journal of Biological Chemistry*, 288(44), 31701-31714.
- Zhang, T., Poudel, A. N., Jewell, J. B., Kitaoka, N., Staswick, P., Matsuura, H., & Koo, A. J. (2016). Hormone crosstalk in wound stress response: wound-inducible amidohydrolases can simultaneously regulate jasmonate and auxin homeostasis in Arabidopsis thaliana. *Journal of Experimental Botany*, 67(7), 2107-2120.
- Schulze, A., Zimmer, M., Mielke, S., Stellmach, H., Melnyk, C. W., Hause, B., & Gasperini, D. (2019). Wound-induced shoot-to-root relocation of JA-Ile precursors coordinates Arabidopsis growth. *Molecular Plant*, 12(10), 1383-1394.
- Floková, K., Feussner, K., Herrfurth, C., Miersch, O., Mik, V., Tarkowská, D., ... & Novák, O. (2016). A previously undescribed jasmonate compound in flowering Arabidopsis thaliana–The identification of cis-(+)-OPDA-Ile. *Phytochemistry*, 122, 230-237.
- Mik, V., Pospíšil, T., Brunoni, F., Grúz, J., Nožková, V., Wasternack, C., ... & Široká, J. (2023). Synthetic and analytical routes to the L-amino acid conjugates of cis-OPDA and their identification and quantification in plants. *Phytochemistry*, 215, 113855.
- Theodoulou, F. L., Job, K., Slocombe, S. P., Footitt, S., Holdsworth, M., Baker, A., ... & Graham, I. A. (2005). Jasmonic acid levels are reduced in COMATOSE ATP-binding cassette transporter mutants. Implications for transport of jasmonate precursors into peroxisomes. *Plant Physiology*, 137(3), 835-840.
- Sanchez Carranza, A. P., Singh, A., Steinberger, K., Panigrahi, K., Palme, K., Dovzhenko, A., & Dal Bosco, C. (2016). Hydrolases of the ILR1-like family of Arabidopsis thaliana modulate auxin

response by regulating auxin homeostasis in the endoplasmic reticulum. *Scientific Reports*, 6(1), 24212.

-- Reviewer comments:

Reviewer #1 (Comments for the Author):

In this well-structured and well-written MS, the Authors focus on the analyses of OPDA-amino-acid molecules in plants, primary in the model plant Arabidopsis. The very limited knowledge on OPDA-Ile is greatly expanded to several OPDA-aa. Several biochemical, genetic, enzymatic and metabolic approaches are employed to describe the accumulation of the OPDA-aa with the aim to define the enzymes involved in the homeostasis of these molecules. The experiments are well designed and controls are included in most cases. Finally, the in vitro analysis of enzymes from different plant species as well as the study of mutant lines of Physcomitrium aim to study the conservation of the OPDA-aa: it is a very nice, although fairly limited, evolutionary approach.

> We appreciated these general considerations from reviewer #1 and agreed that the results from Physcomitrium and spruce included in the previous version of this manuscript embodied a limited evolutionary approach. For this reason, we have decided to focus exclusively on OPDA-aa occurrence in the model species Arabidopsis in this revised version. Another research group is currently preparing a complementary and more in-depth study in the liverwort Marchantia polymorpha. It will be submitted to Plant Physiology as a back-to-back.

Although the MS reads well, there are a number of points that should be addressed:

Major points:

1) Fig 3: What is the rationale to select OPDA-Val and OPDA-Glu as representatives of the 2 groups to design standards isotopically labeled on the amino acid moiety? OPDA-Val is quite active in inducing gene expression whereas OPDA-Glu is almost inactive in inducing this response (Fig 3A). Would it not be better to select a representative OPDA-aa more-active-than-OPDA-Glu?

> We thank reviewer #1 for this suggestion. Following this recommendation we designed new OPDA-aa standards labeled on cis-OPDA moiety (d_5-OPDA-Val and d_5-OPDA-Ile), repeated the in planta-feeding assay and monitored the formation of d_5-derivatives over time. The results from these experiments can be found in the Figure 3C and D, Supplementary Figure S2A and B, and a scheme of the in

planta metabolic conversion of labeled OPDA-Val and OPDA-Ile is reported in Supplementary Figure S2C.

2) Fig 4B: it is very clear that the sextuple gh3 mutant fails to accumulate OPDA-Asp after exogenous OPDA treatment. Since OPDA-Asp is highly induced after wounding, a natural/endogenous stress, it will be very interesting to analyze the biosynthesis of OPDA-Asp after wounding in the sextuple gh3 mutant to further support the conclusions.

> We only detected OPDA-Asp upon treatment with cis-OPDA in plants (Figure 1C) and no OPDA-Asp accumulated upon wounding in our experimental conditions (Figure 1A). However, following this suggested experiment, we monitored the accumulation of OPDA-aa in the gh3 sextuple mutant upon wounding and found that this mutant failed to accumulate OPDA-Glu and OPDA-Phe 2 h post-wounding, whereas OPDA-Ala level were similar between Col-0 and gh3 sextuple mutant. These new results can be found in Figure 4C.

3) Results shown in fig 5 suggest that AtILL6 plays a primary role in hydrolyzing several OPDA-aa. To support the putative role of OPDA-aa as inactive pool of OPDA (to activate the canonical COI1-dependent responses), it may be good to analyses JA-responses (growth inhibition, gene expression, ect) in ill6 mutant and WT plants wound-stressed or/and treated with different OPDA and OPDA-aa. These simple experiments may be very informative.

> We agree that based on results from leaf wounding, ILL6 seems to play a primary role in hydrolyzing OPDA-aa. Nonetheless, when it comes to examine the contribution of ILL6 on the hydrolysis of OPDA-aa upon treatment with OPDA-aa, the lack of ILL6 in the ill6-2 loss-of-function mutant used in this study did not translate in any significant difference of cis-OPDA content, root length and gene expression compared to the wild-type. We believe that together with gene redundancy, the tissue-specific and developmentally controlled expression of ILR1/ILL genes can hinder the physiological significance of the individual ILR1/ILL activity. For further studies, we will plan to generate higher order knock-out mutants and overexpressing lines to overcome this issue.

4) Fig 6B: similar to the previous comments, to study the endogenous function of PpGH3 in the mutant lines, it'd be better to study the accumulation of OPDA-aa after wounding compared to exogenous OPDA treatment.

> We have removed results from Physcomitrium patens in this new version of the manuscript and exclusively focused on the study of the conjugative pathway in Arabidopsis.

5) Fig 6B: what is the rationale to test the accumulation of OPDA-Asp in vivo? The in vitro assay suggest that OPDA-Ala is a likely product of the PpGH3 activity. Please add the analysis of OPDA-Ala in Physcomitrium WT and mutant lines.

> We have removed results from Physcomitrium patens in this new version of the manuscript and exclusively focused on the study of the conjugative pathway in Arabidopsis.

6) Line 145 and generally in the text: please avoid the wording "lower land" referring to Physcomitrium, it is not an accepted scientific nomenclature and, I am afraid, it is simply not correct. Please refer to Physcomitrium as a Bryophyte, moss or non-vascular plant.

> Addressed.

7) Are the markers described in line 213 "THI2.1, VSP1, ZAT10, GRX480, JAZ5 and PDF1.2" both cis-OPDA and JA-responsive? In its current format, it is difficult to understand if the expression of specific genes regulated by cis-OPDA or by JA, or they are generally regulated by both OPDA and JA.

> Addressed. See L233-236 in the revised manuscript.

8) A general concern is about the in vitro enzymatic assay: the analyses of enzymes with limited specificity, such as GH3 and ILR (accepting different substrates, including SA and IAA), suggest particular caution. Authors clearly state that the in vitro activity is a general proxy, and I appreciate that in vivo experiments always support their conclusions.

> We are aware that results from enzymatic assays not always matches with those obtained by studying the same pathway in planta and this is the reason why we reported both results obtained with the two approaches. We are glad that reviewer #1 appreciates our approach.

9) Were the Ppgh3 ko mutants described before? Please add the reference/s. In case these mutants were generated by the Authors, it is important to characterize these mutants: how were they generated (CRISPR-Cas, homologous recombination, T-DNA ect)? How were they confirmed as complete loss-of-function?

> We have removed results from Physcomitrium patens in this new version of the manuscript and exclusively focused on the study of the conjugative pathway in Arabidopsis.

10) Line 347 "These results confirm that cis-OPDA amino acid conjugation is an evolutionarily conserved metabolic pathway in plants" this conclusion is an overinterpretation. Based on these results, it'd be wise to suggest a conclusion similar to: cis-OPDA amino acid conjugation is a conserved metabolic pathway in 2 evolutionary "distant" land plants, the bryophyte Physcomitrium and the angiosperm P. abies.

> We have removed results from Physcomitrium patens and Picea abies in this new version of the manuscript and exclusively focused on the study of the conjugative pathway in Arabidopsis.

Medium issues:

11) Line 98 "JA-Ile is turned over by two inducible and intertwined catabolic pathways. One is oxidative and mediated by cytochrome P450 enzymes of the subfamily 94 (CYP94)" indeed, the oxidation of JA-Ile to JA-Ile-OH was initially suggested to be a catabolic pathway (Koo et al., 2011). However, it has been recently showed that JA-Ile-OH can induce COI1-JAZ interaction, though with a lower affinity compared that of JA-Ile (Heitz et al., 2012; Koo et al., 2014; Jimenez-Aleman et al., 2019; Poudel et al., 2019).

> Addressed. See L108-111.

12) The reactive electrophilic activity of dn-OPDA and OPDA is not clearly introduced. It is probably important to briefly describe the RES activity of these molecules.

> Addressed. See L122-125.

13) Fig 2C: the staining of JAZ9-GUS in mock is very limited. It'd be good to add new images in which basal JAZ9-GUS staining is clear, or maybe it'd be better to remove the data from 35S:JAZ9-GUS analyses in its present format, which in inconclusive. As alternative, the GUS enzymatic activity may be quantified, although this is probably beyond the scope of this manuscript.

> Addressed.

14) Fig 3: is it possible to labeled the OPDA moiety (instead of the aa moiety) of the ({plus minus})-OPDA-aa? It could be used to define more clearly the conclusions proposed.

> Addressed. See also answer to point 1.

15) What is the rationale to test the GH3 proteins of Group II of P. abies? Why were other GH3 proteins discarded?

> We have removed results Picea abies in this new version of the manuscript and exclusively focused on the study of the conjugative pathway in Arabidopsis.

16) Fig 5: "Considering the low hydrolytic activity of recombinant AtILL6 against OPDA-Ala and OPDA-Ile, it was surprising that these two OPDA-aa accumulated in the ill6-2 mutant" in line with the previous comment, a stronger correlation between in vitro activity on OPDA and in vivo OPDA-aa biosynthesis may be obtained in OPDA-feed plants compared to wounded plants. May it be worth to assess OPDA-aa accumulation in WT and hydrolase mutants after exogenous OPDA treatment?

> See answer to point 3.

17) Fig 6B: what is the rationale to generate the Ppgh3 double mutant? The analysis of the single gh3.2 ko already show that GH3.2 has a primary role in OPDA-aa synthesis in vivo, isn't it?

> We have removed results from Physcomitrium patens in this new version of the manuscript and exclusively focused on the study of the conjugative pathway in Arabidopsis.

18) Discussion, line 363 "We further demonstrated that these ({plus minus})-OPDA-aa possess cis-OPDA-like activity on typical transcriptional and physiological cis-OPDA- and JA-responses in Arabidopsis (Figure 2; Figure 3A)" these conclusions are not supported by the results. Data of Figure 3A show that most OPDA-aa fail to induce the expression of most JA-/OPDA-marker genes. Please take into consideration the results shown in Fig 3A and rephrase the sentence.

> We have removed this sentence.

Minor points:

19) Fig 3B: it'd be good to add "in medium" and "in plant" in the sub-panel to help the readers.

> Addressed.

20) AtGH3.1 to .6 are clearly involved in the hydrolysis of OPDA-Asp (Fig 4): it'd be good to test gene expression and root growth inhibition in WT vs gh3 sextuple mutant in response to OPDA-Asp. Did you perform these easy but informative experiments?

> Yes, we have performed these experiments and did not observe any significant difference between gh3 sextuple mutant and Col-0. Results from the root growth inhibition assay reported in Figure 2A and B already showed that OPDA-Asp, together with OPDA-Glu have no effect on root growth already in Col-0 plants. OPDA-Asp was also found ineffective in inducing the expression of the selected marker genes (see Figure 3A). Therefore, it should not be expected that OPDA-Asp has any effect on root growth and gene expression in the gh3 sextuple mutant.

21) My curiosity: do algae accumulate OPDA-aas?

> We did not inspect the OPDA conjugative pathway in algae, but we agree with reviewer #1 that it would be very interesting to investigate it.

Reviewer #2 (Comments for the Author):

This study builds on the authors' prior work showing that OPDA-Ile and other OPDA-amino acid conjugates (OPDA-AAs) are naturally occurring compounds in Arabidopsis. Given current interest in OPDA signaling and the activity of JA-Ile, the topic is of general interest. Here, the authors report data to support the claim that OPDA is conjugated to AAs under stress conditions to stimulate OPDA formation. Some new information on the metabolism of OPDA-AAs by GH3 conjugases and ILR hydrolases is reported. The biological role of OPDA-AA conjugates remains unclear. Several suggestions are offered to improve the work and its presentation.

> We agreed with the general criticism of reviewer #2. In this revised manuscript, we provided additional evidence about the metabolic fate, function, and formation of OPDA-aa. We demonstrate that OPDA-aa function in the catabolism or temporary storage of cis-OPDA in stress responses instead of acting as chemical signals per se, confirming a homeostatic regulation of the cytosolic cis-OPDA pool and propose the existence of a subcellular pathway controlling nuclear cis-OPDA levels.

Main points

This lab recently reported (Mik et al 2023) that OPDA-Ile levels are induced in wounded Arabidopsis leaves. But in the present study they say that OPDA-Ile cannot be detected. This discrepancy needs to be resolved, and calls into doubt the premise that OPDA-Ile or other OPDA-AAs are biologically relevant stress signals as previously suggested.

> Addressed. See 187-195.

A shortcoming of the work is its focus on the action and metabolism of exogenous OPDA-AAs. Although this line of investigation is important for establishing what enzymes have the capacity to metabolize OPDA-AAs, it does not prove how endogenous pools of OPDA-AAs are metabolized or what the endogenous compounds might be doing.

> The major issue when studying the endogenous occurrence of OPDA-aa concerns the low abundance of these compounds that are not detected under steady-state growth conditions but only upon stress. We addressed this issue by testing the effect of different types of stress (biotic and abiotic and by exogenous application of OPDA and OPDA-aa). We observed that all these stress stimuli elicited the formation of OPDA-aa, highlighting that the amino acid conjugation of OPDA is likely a general, rather than a specific, plant response to stress. Since OPDA-aa were formed at a much higher level upon exogenous application of OPDA than in response to biotic and abiotic stress, we took advantage of this strong plant response to examine whether OPDA-aa retained any cis-OPDA-like activity and identify the enzymes responsible for their synthesis and hydrolysis.

We showed that the synthesis and hydrolysis of cis-OPDA amino acid conjugates are mediated by members of the amidosynthetase GH3 and the amidohydrolase ILR1/ILL families. The identification of the metabolic enzymes responsible for OPDA-aa synthesis and hydrolysis allowed us to demonstrate that OPDA-aa are rapidly and endogenously accumulated after wounding in amidohydrolase mutants and amidohydrolases, whose genes are upregulated upon wounding (Widemann et al., 2013; Zhang et al., 2016), are responsible for the rapid hydrolysis of OPDA-aa (Fig. 5B). In this revised manuscript, we additionally showed that the identified cis-OPDA-conjugating GH3s redundantly contribute to the formation of OPDA-aa upon wounding (Fig. 4C).

Collectively, this evidence demonstrates that oppositely to JA, and similarly to IAA, amino acid conjugation of cis-OPDA is not required for plant hormone activation, OPDA-aa function in the catabolism or temporary storage of cis-OPDA in stress responses instead of acting as chemical signals per se.

Considering that OPDA-aa are endogenous low abundant cis-OPDA derivatives only formed upon stress and rapidly hydrolyzed back to cis-OPDA in response to wounding, in this revised manuscript, we investigated whether the formation of OPDA-aa might be localized at the damaged site of the leaf. We showed that OPDA-aa equally accumulated in the wounded and unwounded area of damaged leaves in Col-0 and ill6 loss-of-function mutant, demonstrating that the low abundance of OPDA-aa is not a consequence of a tissue-specific OPDA-aa

formation (Fig. 6A). Interestingly, upon wounding, cis-OPDA accumulated predominantly at the damaged site in Col-0 leaves. In contrast, the cis-OPDA distribution was widespread all over the leaf in the ill6-2 mutant, albeit the leaf damage induced an increase of cis-OPDA level in both genotypes. This suggests that ILL6 is required to channel cis-OPDA at the damaged site in response to wounding.

Endogenous conjugation of cis-OPDA (and deconjugation of OPDA-aa) implies that not all the cis-OPDA is metabolized in the peroxisome and that plants have evolved mechanisms to regulate its homeostasis through transport (Schulze et al., 2019) and metabolism (Floková et al., 2016; Mik et al., 2023; this study), similarly to other important phytohormones like JA and IAA. Part of the chloroplast-derived cis-OPDA accesses the cytosol, perhaps through the postulated "leak" pathway (Theodoulou et al., 2005). We included new proofs about the subcellular localization of GH3 enzymes, demonstrating for the first time a dual cytosolic and nuclear localization of Arabidopsis GH3.3 protein (Fig. 4D and E) that points to cytoplasm and nucleus as potential sites for GH3 activity. This new evidence, together with the previously described ER localization of Arabidopsis ILR1/ILL enzymes (Zhang et al., 2016; Sanchez Carranza et al., 2016), confirms a homeostatic regulation of the cytosolic cis-OPDA pool in the ER and prompted us to propose a model for cis-OPDA and OPDA-aa subcellular homeostasis (Fig. 6B). Once synthesized in the chloroplast, cis-OPDA is mainly reduced and β-oxidized to produce JA in the peroxisome. Part of the chloroplast-derived cis-OPDA may reach the cytosol, where GH3 enzymes can conjugate it with amino acids to form OPDA amino acid conjugates (OPDA-aa). OPDA amide conjugates might be hydrolyzed back to cis-OPDA in the endoplasmic reticulum (ER) by ILR1/ILL enzymes, and cis-OPDA may be then released into the nucleus. Nuclear cis-OPDA can activate the expression of cis-OPDA-responsive genes or be inactivated by conjugation with amino acids by nuclear GH3s. According to this model, cis-OPDA amino acid conjugation might be important in regulating nuclear cis-OPDA levels through the ER.

References:

- Widemann, E., Miesch, L., Lugan, R., Holder, E., Heinrich, C., Aubert, Y., ... & Heitz, T. (2013). The amidohydrolases IAR3 and ILL6 contribute to jasmonoyl-isoleucine hormone turnover and generate 12-hydroxyjasmonic acid upon wounding in Arabidopsis leaves. *Journal of Biological Chemistry*, 288(44), 31701-31714.
- Zhang, T., Poudel, A. N., Jewell, J. B., Kitaoka, N., Staswick, P., Matsuura, H., & Koo, A. J. (2016). Hormone crosstalk in wound stress response: wound-inducible amidohydrolases can simultaneously regulate jasmonate and auxin homeostasis in Arabidopsis thaliana. *Journal of Experimental Botany*, 67(7), 2107-2120.
- Schulze, A., Zimmer, M., Mielke, S., Stellmach, H., Melnyk, C. W., Hause, B., & Gasperini, D. (2019). Wound-induced shoot-to-root relocation of JA-Ile precursors coordinates Arabidopsis growth. *Molecular Plant*, 12(10), 1383-1394.

- Floková, K., Feussner, K., Herrfurth, C., Miersch, O., Mik, V., Tarkowská, D., ... & Novák, O. (2016). A previously undescribed jasmonate compound in flowering Arabidopsis thaliana–The identification of cis-(+)-OPDA-Ile. *Phytochemistry*, 122, 230-237.
- Mik, V., Pospíšil, T., Brunoni, F., Grúz, J., Nožková, V., Wasternack, C., ... & Široká, J. (2023). Synthetic and analytical routes to the L-amino acid conjugates of cis-OPDA and their identification and quantification in plants. *Phytochemistry*, 215, 113855.
- Theodoulou, F. L., Job, K., Slocombe, S. P., Footitt, S., Holdsworth, M., Baker, A., ... & Graham, I. A. (2005). Jasmonic acid levels are reduced in COMATOSE ATP-binding cassette transporter mutants. Implications for transport of jasmonate precursors into peroxisomes. *Plant Physiology*, 137(3), 835-840.
- Sanchez Carranza, A. P., Singh, A., Steinberger, K., Panigrahi, K., Palme, K., Dovzhenko, A., & Dal Bosco, C. (2016). Hydrolases of the ILR1-like family of Arabidopsis thaliana modulate auxin response by regulating auxin homeostasis in the endoplasmic reticulum. *Scientific Reports*, 6(1), 24212.

The simplest interpretation of data in Fig 2 is that exogenous JA, OPDA and OPDA-aa are all converted by JAR1 to JA-Ile in planta. If this is true, it is uninformative with regard to the role (if any) of endogenous OPDA-AAs.

> We agree with reviewer #2's conclusion about results from Figure 2A and B. We have indeed repeated this growth inhibition assay by treating the JA-Ile deficient gh3.10-2,jar1-11 double ko mutant with (±)-OPDA-aa and collected even more substantial evidence supporting that the root-growth inhibitory effect of these (±)-OPDA-aa required in planta conjugation of JA to Ile. Nonetheless, our data confirm the existence of a homeostatically regulated cis-OPDA pool between the cytosol and the endoplasmic reticulum, and our findings hint at a subcellular pathway that might directly control nuclear cis-OPDA levels by ER-to-nucleus flux of cis-OPDA. However, we agree with reviewer #2 that we must consider homeostasis of OPDA-aa as an additional determinant in different stress responses and developmental processes.

The authors provide some evidence that several GH3s/ILRs are involved in metabolizing exogenous OPDA. But the data is not definitive and raises many questions. Rigorous proof requires analysis of endogenous ODPA-AA levels in gh3/ilr mutants. The extreme low levels (10 to 100-fold less than JA-Ile) of OPDA-AAs in Arabidopsis leaves makes it challenging to address this question and also calls into question the biological relevance of these compounds.

> We agree with reviewer #2 that the low abundance of these compounds and the fact that they are not detected under steady-state growth conditions but only upon stress represent a major challenge when studying the biological relevance of OPDA-aa. Nonetheless, we find fascinating that oppositely to JA, and similarly to IAA, amino acid conjugation of cis-OPDA is not required for plant hormone activation. OPDA-aa serve as a reservoir of inactive cis-OPDA. We suggest that an ER-to-nucleus flux of cis-OPDA might underpin nuclear cis-OPDA uptake.

Accordingly, OPDA-aa are hydrolyzed by ILR1/ILLs in the ER. Consequently, free cis-OPDA can reach the nucleus, acting as a signaling molecule, or be inactivated by GH3 enzymes. A model for cis-OPDA and OPDA-aa subcellular pathway is reported in Figure 6B of the revised manuscript.

A second shortcoming of the work is the lack of any new information about the physiological function of OPDA-AAs. A simple hypothesis is that most stress-induced production of OPDA in Arabidopsis enters the peroxisome (for JA biosynthesis) or gets conjugated into Arabidopsides. A small amount of this OPDA, however, may "leak" into the cytosol where it is toxic because of the high reactivity of the compound. Conjugation to AAs may reflect a mechanism to neutralize this toxicity. It might be interesting to look at the role of cytosolic OPR2 in OPDA/OPDA-AA metabolism.

> We thank reviewer #2 for raising this point. Our data confirm the presence of cytosolic cis-OPDA that is temporarily conjugated to amino acids and moves as OPDA-aa to the ER where it is hydrolyzed back to free cis-OPDA and either accesses the nucleus or moves to the peroxisome. We have not investigated a possible role of cytosolic OPR2 in OPDA/OPDA-aa metabolism, but we agree that it might be an exciting possibility for a further study.

Other points

Rather than stating the effects of OPDA-aa are mediated in a JA-Ile-dependent manner, it is more accurate to say that the effects occur in a JAR1-dependent manner.

> We have repeated the root growth inhibition assay using the JA-Ile deficient gh3.10-2,jar1-11 double ko mutant.

The Introduction should mention that cis-OPDA is a highly reactive molecule owing to the unsaturated carbonyl group.

> Addressed. See L122-125.

Figure legends should state the concentration of OPDA-AA used.

> Addressed.

As a matter of writing style: breaking up the text into smaller paragraphs, each focused on a specific point that is delineated in the first sentence of the paragraph, would make for greater ease of reading and comprehension.

> We implemented the writing style by starting more often new paragraphs.

In the Introduction, references cited in support of previous research are generally okay but a few omissions were noted, as indicated below for the authors' consideration.

> *We have implemented the references cited in the introduction.*

20-Aug-2024

Dr. Federica Brunoni
Laborator rustovych regulatoru
Olomouc
Czech Republic

RE: Homeostasis of cis-OPDA amide conjugates upon stress responses

Dear Dr. Brunoni:

We have received reviews of your manuscript, which you may view at the end of this letter. On the basis of the advice received and my own evaluation, I believe that, pending revision, this article may ultimately be acceptable for publication in Plant Physiology.

Both reviewers list a number of minor issues, which can be solved mostly by textual editing. Reviewer #1 additionally has some important experimental concerns, which I expect you to address. This could comprise pictures of leaves at later stages of infection (issue #1), additional control samples for the immunoblot analysis to support the GH3 localisation conclusions (issue #2) and imaging of a control leaf of e.g. the aos mutant (issue #3).#####

When you are ready to submit your revision, click the below link and find the red arrow under your Author Tasks heading for this revision submission.

If you want to visit this version of the manuscript, you may click the link below and then click on the folder that reads "Post Decision Manuscripts".

Link Not Available

When uploading the revised version of this article, please be sure to include as complete an answer as possible to the referees' criticisms during the first round of reviews in the "response to reviewers" section.

Plant Physiology considers a revision for up to 60 days after a manuscript has been provisionally accepted; limited extension of this timeframe is at the discretion of the Monitoring Editor. A reminder notice will be sent to you 2 weeks before the deadline, at which time you may choose to contact the editorial office to request an extension; if so, please provide a justification for your request. After the expiration of this deadline, the article would need to be submitted as a resubmission and would be handled as such.

Thank you very much for giving us an opportunity to review this work. I look forward to receiving the next version.

Sincerely,

Editor, Plant Physiology

Want to add this revision deadline to your calendar? Click below!

--- Reviewer comments:
Reviewer #2 (Comments for the Author):

This re-submitted revised manuscript employs a powerful combination of biochemical, analytical, and genetic approaches to investigate the metabolism and putative function of OPDA amino acid conjugates (OPDA-AAs). The authors' performed new experiments to make the paper stronger (with a few exceptions; see below). They also re-interpret their data to reach the conclusion that OPDA-Ile does not function as a canonical signal in the JA response pathway.The new d5-labeling experiments provide strong evidence that the biologically activity of exogenous OPDA-AAs results from in vivo hydrolysis, conversion to JA,

and subsequent conjugation to Ile. This is a cautionary lesson regarding how to interpret the biological activity of exogenous compounds, and strongly suggests that OPDA-Ile is not a biologically relevant signaling compound. A few minor suggestions are offered for further improvement of the presentation.

Main points
1. Line 185-192: I appreciate the authors addressing the discrepancy between the reported results and their earlier results (Mik et al) with respect to detection of OPDA-Ile in UW vs W leaves. Here they report that wounding does not induce OPDA-Ile accumulation. From what I can tell, OPDA-Ile was only detected in Botrytis infected leaves. This made me wonder what these leaves look like after 3 days of infection; are the leaves undergoing severe necrosis and likely degradation of OPDA-rich chloroplast lipids? This issue has bearing on understanding the conditions in which OPDA-Ile is produced.

2. The GH3.3 localization data in Fig 4D-F is not as strong as it could be and in fact seems to raise more questions than it answers. My understanding is that many previous studies have established GH3s as cytosolic -- not nuclear -- proteins. The DiMombro 2018 paper stated to cite dual localization of GH3 to cytosol/nucleus actually states that the predominant local is the cytosol. Here the authors claim that GH3.3 is also located in the nucleus. Given this surprising finding, additional controls seem warranted. For example a GFP alone control is not included; Fig 4D left panel is labeled "GFP" but I think it is actually GH3.3-GFP (and should be labeled so) according to the legend. One concern is that the nuclear localization results from GFP cleavage in vivo. Fig 4F attempts to address this issue but interpretation of the western blot would benefit from lanes showing GFP alone and a WT control (without GFP), in order establish the identity of the band as GH3.3-GFP. In short, I'm not convinced about the nuclear localization of GH3.3. Omitting the localization data would not detract from the main message.

3.The mass spec imaging data in Fig 6A is interesting and I think somewhat novel. I am, however, surprised that low abundance compounds such as OPDA-Ile, which was not detected in wounded leaves using more sensitive LC-MS (previous figure), is detected in wounded leaves by imaging. This raises the question of whether the signal obtained is specific for OPDA-Ile (or other derivatives). It seems that a key control is imaging of a control leaf (eg., aos mutant) that does not produce OPDA.

Minor points
The reference in the abstract to nuclear vs cytosolic pools of OPDA is completely speculative and thus is better suited for the Discussion than the Abstract.

Line 207-209: change "indicate" to "suggest": The data have no bearing on whether these compounds "allow the plant to adapt to stress".

Line 112; 247-248: Thines et al 2007 was the first to show that OPDA does not promote formation of JAZ-COI1 complexes

Line 98: Sheard et al (2010 Nature) should be credited as first having described JA-Ile as "molecular glue".

Reviewer #3 (Comments for the Author):

Siroka et al quantify the temporal dynamics of OPDA-aa conjugates produced in Arabidopsis after wounding and B. cinerea infection, and test if any of them have a biological function when applied externally. Contrary to several confusing previous reports, by using a jar1 mutant impaired in bioactive JA-Ile production, this work shows that these OPDA-aa conjugates stunt root growth and impact molecular phenotypes (eg. JAZ1 degradation, gene expression) by their in-planta conversion to JA-Ile and not autonomously. In fact, while OPDA-aa are not detectable before wounding, they accumulate at later time points than JA and JA-Ile. By combining state-of-the-art analytics and synthesis with mutant analysis, this work provides a much-needed clarification to an oftentimes erroneous interpretation of molecular bioactivity in the JA field. Furthermore, the authors identify GH3 amido-synthetases and ILR1/ILL amido-hydrolases regulating OPDA-aa homeostasis, potentially impacting OPDA catabolism or its temporary storage. The manuscript is clearly written, and the data is analysed rigorously.

Here are a few minor suggestions the authors may consider :
- Lines 64-66, 162-163, the subcellular distribution of cis-OPDA pools was not characterized in this paper and would require a whole set of new methods and experiments. I recommend the authors to minimize comments on this topic across the manuscript, and restrict putative interpretations to the discussion.

- Similarly, the overexpression (OE) of fusion proteins in protoplasts is not the state-of-the art method to localize proteins, nor is their OE in stable transgenic lines (Fig.4). The authors should clearly state the limitations of these observations. In any case, does GH3.3 OE lead to growth phenotypes, or perturbed OPDA-aa profiles?

- Line 103, ILR1 mentioned twice in the same sentence. Verify

- Legend Fig3A. Specify at which timepoint samples were harvested after treatment

Reviewer #2 (Comments for the Author):

This re-submitted revised manuscript employs a powerful combination of biochemical, analytical, and genetic approaches to investigate the metabolism and putative function of OPDA amino acid conjugates (OPDA-AAs). The authors' performed new experiments to make the paper stronger (with a few exceptions; see below). They also re-interpret their data to reach the conclusion that OPDA-Ile does not function as a canonical signal in the JA response pathway. The new d5-labeling experiments provide strong evidence that the biologically activity of exogenous OPDA-AAs results from in vivo hydrolysis, conversion to JA, and subsequent conjugation to Ile. This is a cautionary lesson regarding how to interpret the biological activity of exogenous compounds, and strongly suggests that OPDA-Ile is not a biologically relevant signaling compound. A few minor suggestions are offered for further improvement of the presentation.

Main points
1. Line 185-192: I appreciate the authors addressing the discrepancy between the reported results and their earlier results (Mik et al) with respect to detection of OPDA-Ile in UW vs W leaves. Here they report that wounding does not induce OPDA-Ile accumulation. From what I can tell, OPDA-Ile was only detected in Botrytis infected leaves. This made me wonder what these leaves look like after 3 days of infection; are the leaves undergoing severe necrosis and likely degradation of OPDA-rich chloroplast lipids? This issue has bearing on understanding the conditions in which OPDA-Ile is produced.

> OPDA-Ile was the first identified and studied OPDA-aa (Phytochemistry, 2016, 122: 230-237; PloS one, 2016, 11.9: e0162829), and similarly to the other OPDA-aa, is a low abundant OPDA metabolite not detected under steady-state growth conditions but only upon stress. In this study, OPDA-Ile was detected in seedlings infected by the necrotrophic fungal pathogen Botrytis cinerea, and pictures of B. cinerea- and mock-inoculated seedlings 4 days after infection are provided in Figure 1 for reviewers that can be found at the end of this document, as requested by the reviewer.

Furthermore, we emphasize that our analytical method can quantify the compounds above the limit of detection. When we do not detect a specific compound, it means that either that compound is not present or is below the limit of detection. When detected, OPDA-aa levels ranged between 1 to 10 pmol/g fresh weight (FW), making them low abundant (and sometimes even close to the limit of detection). This means that the failure to detect OPDA-Ile in wounded leaves in this study might be a combination of biological (different plant growth conditions and plant age) and analytical factors. Overall, our results demonstrate that biotic, abiotic, and exogenous applications of OPDA and OPDA-aa are stimuli that elicit OPDA conjugation with several amino acids (and not only Ile) and that different OPDA-aa are more or less prone to be hydrolyzed.

2. The GH3.3 localization data in Fig 4D-F is not as strong as it could be and in fact seems to raise more questions than it answers. My understanding is that many previous studies have established GH3s as cytosolic -- not nuclear -- proteins. The DiMombro 2018 paper stated to cite dual localization of GH3 to cytosol/nucleus actually states that the predominant local is the cytosol. Here the authors claim that GH3.3 is also located in the nucleus. Given this surprising finding, additional controls seem warranted. For example a GFP alone control is not included;

Fig 4D left panel is labeled "GFP" but I think it is actually GH3.3-GFP (and should be labeled so) according to the legend. One concern is that the nuclear localization results from GFP cleavage in vivo. Fig 4F attempts to address this issue but interpretation of the western blot would benefit from lanes showing GFP alone and a WT control (without GFP), in order establish the identity of the band as GH3.3-GFP. In short, I'm not convinced about the nuclear localization of GH3.3. Omitting the localization data would not detract from the main message.

> We understand the reviewer's concerns about the nuclear localization of GH3.3-GFP. We were also surprised about our finding, as it diverged from the general concept that GH3s are exclusively localized in the cytosol. Unfortunately, there are not many previous studies establishing the intracellular localization of GH3 enzymes. To our knowledge, this aspect has only been investigated in a few reports. It was attempted by Ludwig-Müller et al., who reported cytosolic localization of Physcomitrium patens GH3-1 by inspecting the GFP signal in P. patens protoplasts transfected with 35S:GH3-1:GFP (New phytologist, 2009, 181.2: 323-338). Subsequently, Di Mambro et al. investigated whether the ER or the cytosol is the compartment of activity of Arabidopsis GH3.17 by subcellular fractionation on an isopycnic sucrose density gradient. GH3.17-GFP was exclusively detected in the fractions where the cytosolic marker NES-YC3.6 was also detected, and no GH3.17-GFP polypeptides were localized in fractions containing the ER. Thus, the authors concluded that GH3.17-GFP is mainly localized in the cytosol (Current Biology, 2019, 29.7: 1199-1205. e4.). While in these previous reports the GH3 subcellular localization was studied in moss or Arabidopsis considering only two cellular compartments (ER and cytosol), a localization study of Arabidopsis GH3s which considers the whole cell was still lacking. In this study, we inspected the intracellular localization of Arabidopsis GH3.3 by transiently expressing 35S-GFP-AtGH3.3 in Arabidopsis protoplasts and generating Arabidopsis transgenic lines stably expressing UBQ10-GFP-GH3.3. We monitored the fluorescent signal originating from the AtGH3-GFP fusion protein in the whole cell for the first time. Both assays returned the same intracellular localization pattern, and the immunoblotting confirmed that the GFP-derived signal originated from the GFP-GH3.3 fusion protein and not free GFP.
We are aware that the subcellular localization of GH3s should also be further investigated, and we are currently working on it. Our future research will focus on understanding the factors that influence the dual cytosolic and nuclear localization of GH3 enzymes. Nonetheless, these additional results will be part of another manuscript in which we focus on studying the activity of GH3 enzymes in the nucleus. While the primary goal of our study was not to assess the subcellular localization of GH3 enzymes, we believe that the discovery of their potential dual cytosolic and nuclear localization is a significant contribution to the scientific community. This essential information should be widely available and considered in future research and studies. We have included the lanes showing the GFP alone and wild-type controls in Figure 2 for reviewers that can be found at the end of this document, as requested by the reviewer.

3.The mass spec imaging data in Fig 6A is interesting and I think somewhat novel. I am, however, surprised that low abundance compounds such as OPDA-Ile, which was not detected in wounded leaves using more sensitive LC-MS (previous figure), is detected in wounded leaves by imaging. This raises the question of whether the signal obtained is specific for OPDA-Ile (or other derivatives). It seems that a key control is imaging of a control leaf (eg., aos mutant) that does not produce OPDA.

> DESI-MSI is a technique that allows for real-time visualization of analytes on surfaces with minimal sample preparation, reducing the risk of signal loss. In contrast, LC-MS/MS requires more extensive sample preparation, including homogenization and extraction, which can concentrate samples but also introduce

potential signal loss. DESI-MSI can sometimes be more sensitive in detecting certain compounds due to lower matrix effects, while LC-MS/MS can be affected by higher matrix effects, potentially obscuring low-abundance compounds, such as OPDA-Ile. This means DESI-MSI may have lower detection limits for specific analytes, making it suitable for detecting low-abundance compounds, whereas LC-MS/MS might not detect them as effectively.
We agree with the reviewer's observation that the signal of OPDA-Ile is detected in the DESI-MSI results from Col-0 wounded leaves (although the signal is extremely low) but not in the LC/MS-MS measurements, which can be explained with a different limit of detection intrinsic of these two analytical methods. Altogether, DESI-MSI is ideal for spatial distribution studies and real-time analysis, while LC-MS/MS is suitable for detailed quantitative and qualitative analysis of complex mixtures. Both techniques have their strengths and can complement each other depending on the analysis needs. However, DESI-MSI can sometimes raise questions about signal specificity. It is important to mention that high-resolution measurement, determination of exact masses of analytes, and their subsequent MS/MS fragmentation guarantee unambiguous identification. On the other hand, LC-MS/MS typically offers high specificity due to the separation step in liquid chromatography but did not demonstrate sufficient sensitivity in our experimental setup.

Minor points
The reference in the abstract to nuclear vs cytosolic pools of OPDA is completely speculative and thus is better suited for the Discussion than the Abstract.

> Addressed.

Line 207-209: change "indicate" to "suggest": The data have no bearing on whether these compounds "allow the plant to adapt to stress".

> Addressed.

Line 112; 247-248: Thines et al 2007 was the first to show that OPDA does not promote formation of JAZ-COI1 complexes

> Addressed.

Line 98: Sheard et al (2010 Nature) should be credited as first having described JA-Ile as "molecular glue".

> Addressed.

Reviewer #3 (Comments for the Author):

Siroka et al quantify the temporal dynamics of OPDA-aa conjugates produced in Arabidopsis after wounding and B. cinerea infection, and test if any of them have a biological function when applied externally. Contrary to several confusing previous reports, by using a jar1 mutant impaired in bioactive JA-Ile production, this work shows that these OPDA-aa conjugates stunt root growth and impact molecular phenotypes (eg. JAZ1 degradation, gene expression) by their

in-planta conversion to JA-Ile and not autonomously. In fact, while OPDA-aa are not detectable before wounding, they accumulate at later time points than JA and JA-Ile. By combining state-of-the-art analytics and synthesis with mutant analysis, this work provides a much-needed clarification to an oftentimes erroneous interpretation of molecular bioactivity in the JA field. Furthermore, the authors identify GH3 amido-synthetases and ILR1/ILL amido-hydrolases regulating OPDA-aa homeostasis, potentially impacting OPDA catabolism or its temporary storage. The manuscript is clearly written, and the data is analysed rigorously.

Here are a few minor suggestions the authors may consider:
- Lines 64-66, 162-163, the subcellular distribution of cis-OPDA pools was not characterized in this paper and would require a whole set of new methods and experiments. I recommend the authors to minimize comments on this topic across the manuscript, and restrict putative interpretations to the discussion.

> Addressed.

- Similarly, the overexpression (OE) of fusion proteins in protoplasts is not the state-of-the art method to localize proteins, nor is their OE in stable transgenic lines (Fig.4). The authors should clearly state the limitations of these observations. In any case, does GH3.3 OE lead to growth phenotypes, or perturbed OPDA-aa profiles?

> Transient expression of proteins fused with a fluorescent tag is widely used in protein localization studies. Using a constitutive promoter has advantages when the gene of interest is typically expressed at low levels and/or tissue-specifically. Such a strategy, for example, was used to assess the subcellular localization of ILR1/ILL enzymes. Sanchez Carranza et al. showed the ER localization of ILR1/ILL enzymes by transiently co-expressing the translational fusions of AtILR1, ILL2, and ILL6 with GFP with an ER-mCherry marker in Arabidopsis protoplasts (Scientific Reports, 2016, 6.1: 24212 2016). Zhang et al. reported the same subcellular localization pattern for CFP-fused AtIAR3 and ILL6 transiently co-expressed with CYP94B3-mRFP in tobacco leaf epidermal cells (Journal of Experimental Botany, 2016, 67.7: 2107-2120). In both studies, the constitutive promoter 35S was used to express the ILR1/ILL fusion proteins.
In this study, we have investigated the intracellular localization of GH3 enzymes by both transient and stable expression methods, knowing that more evidence than only results from transient expression is needed to assess the correct subcellular localization of GH3s. We used constitutive promoters such as 35S and UBQ10 because our gene of interest was expressed at low levels. Nonetheless, we are aware that the subcellular localization of GH3s should also be investigated by expressing the fusion protein under the native promoter, and we are currently working on it. This additional result will be part of another manuscript in which we focus on studying the activity of GH3 enzymes in the nucleus.
While the primary goal of our study was not to assess the subcellular localization of GH3 enzymes, we believe that the discovery of their potential dual cytosolic and nuclear localization is a significant contribution to the scientific community. This essential information should be widely available and considered in future research and studies. Please also refer to the answer above to the reviewer #2 point 2 on this topic, as it provides additional insights into our research and its implications.

Our work is a continuous process, and we are currently in the process of phenotyping and profiling the GFP-GH3 OE line. Unfortunately, we do not have these results yet, but we are committed to sharing our findings as soon as they are available.

- Line 103, ILR1 mentioned twice in the same sentence. Verify

> Addressed.

- Legend Fig3A. Specify at which time point samples were harvested after treatment

> Addressed.

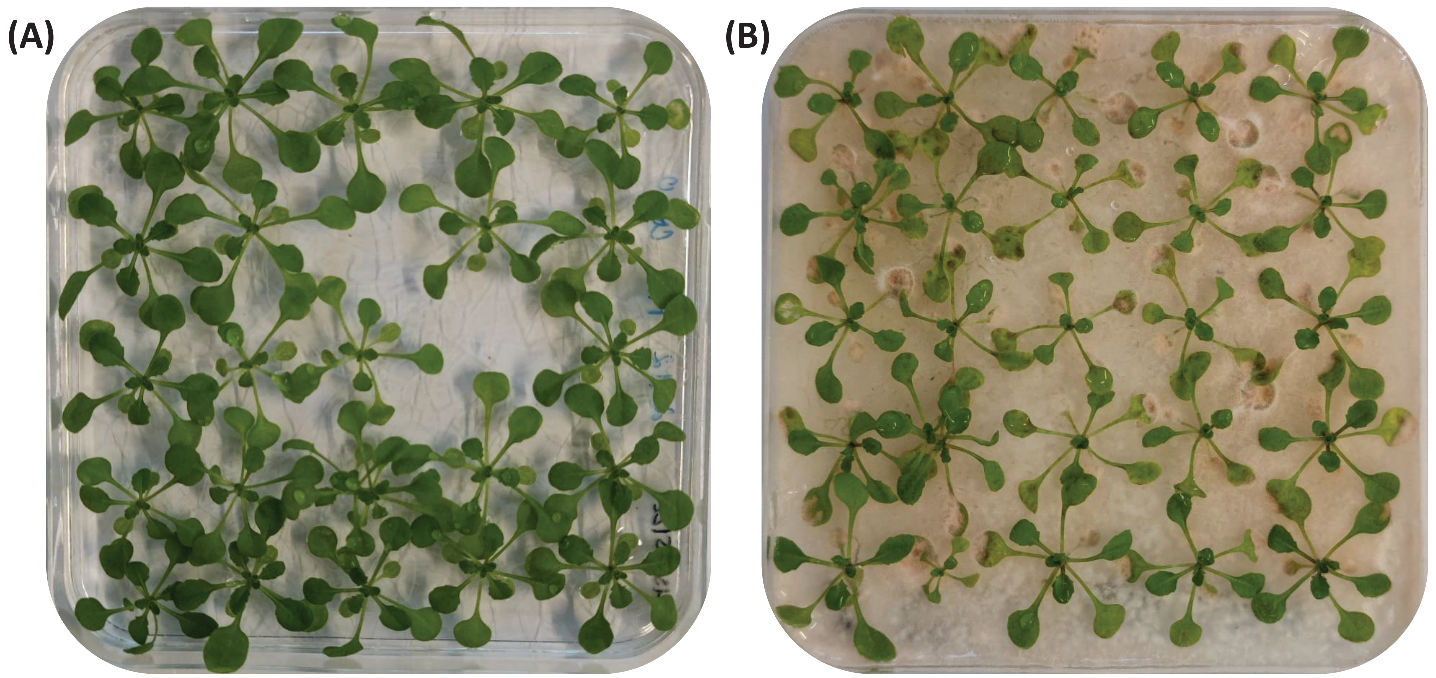

Figure 1 for reviewers *Botrytis cinerea* infection on Arabidopsis seedlings. Two-week-old *in vitro*-grown Arabidopsis Col-0 seedlings were either mock-inoculated (A) or infected with a suspension of *B. cinerea* spores (B). Pictures were taken 4 days after the inoculation.

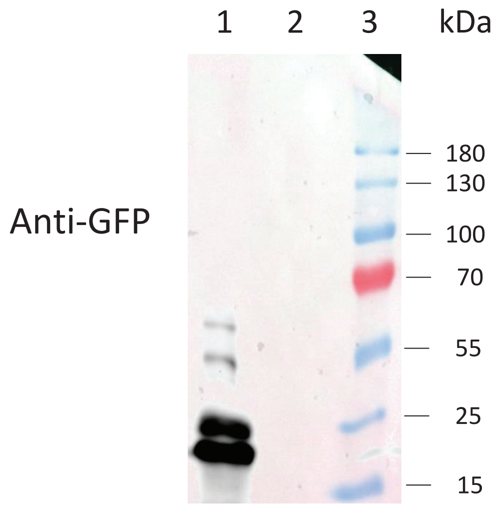

Figure 2 for reviewers Western-Blot analysis of free GFP and Arabidopsis wild-type. Lane 1, GFP from *E. coli* cell lysate of IPTG-induced harboring GFP; lane 2, total protein extracted from Arabidopsis Col-0 seedling; lane 3, protein marker. Molecular weight of GFP protein: 27 kDa.

18-Oct-2024

Dr. Federica Brunoni
Laborator rustovych regulatoru
Olomouc
Czech Republic

RE: Homeostasis of cis-OPDA amide conjugates upon stress responses

Dear Dr. Federica Brunoni:

We are pleased to accept your manuscript for publication in the Plant Physiology. This acceptance is contingent on revision based on the comments of our reviewers. In particular, please consider the following:

Both reviewers remain critical about certain aspects of the paper as detailed in their comments. Yet, both reviewers also consider you manuscript interesting and much of the data provided will be useful to the field. I agree with these impressions and therefore your manuscript can be accepted under the condition you add a section 'Limitations to this study' in the discussion section. Within this section you should critically assess your own data/approach, particularly within the frame of the remaining concerns of the reviewers. This should not be an extensive section but nonetheless it should do a decent job in addressing the very valid concerns of the two reviewers. Fyi, if you want inspiration for such as session, consult the papers that appear in the journal Cell. These have very relevant and useful 'Limitations to this study' paragraphs at the end of the discussion. That is the kind of addition I would like to suggest to add.

Please highlight all changes and include a detailed annotation to changes to the text, with line numbers, and noting your responses to the comments.

To submit your revised manuscript, click:

Link Not Available

If you cannot return the revised manuscript within 8 weeks of receipt, please let us know. Otherwise, we will assume that you have elected not to revise the manuscript and are withdrawing it.

Thank you for allowing us to review your work. We look forward to hearing from you soon.

Sincerely,

Editor, Plant Physiology

-- Reviewer comments:
Reviewer #2 (Comments for the Author):

I thank the authors for their response to my comments on the revised manuscript.

If the nuclear localization results are included, I would encourage the authors to discuss the limitations of their approach (eg, transient overexpression; effect of tag on localization signal, etc) in the event this turns out to be an artifact.

I am not familiar with the specifics of DESI-MSI and will defer to those with more expertise. For the record, I remain skeptical that a MS imaging approach can specifically identify a compound whose extreme low abundance cannot be detected by normal LC-MS. Again, the authors' should discuss the limitations of the approach.

Reviewer #4 (Comments for the Author):

As stated in my earlier review, the authors present very solid results on the role of OPDA-aa conjugates after wounding and necrotroph infection that will clarify somewhat conflicting interpretations in the field.

I had only two recommendations (apart from minor changes in the text), which were unfortunately not considered by the authors

1. GH3.3 localization is not a major finding of this work, but drawing conclusions from OE lines may prove inaccurate in the long term (in agreement with Rev2), particularly if plant selection was not done properly. Shifting the data to the supplement and toning down the localization interpretation was not done in the revised version, but is still advisable. In fact, OE of cytosolic proteins often results in nuclear signals as pointed out by Rev2 as well

2. As GH3.3 OE lines were used to show localization (Fig4), it remains unclear how come the authors did not perform any phenotypic analyses during the 2-month revision time. If the authors used T1 or segregating T2 lines to draw conclusions for localisation, it only strengthens the concerns raised in point1.

--- Reviewer comments:
Reviewer #2 (Comments for the Author):

I thank the authors for their response to my comments on the revised manuscript.

If the nuclear localization results are included, I would encourage the authors to discuss the limitations of their approach (eg, transient overexpression; effect of tag on localization signal, etc) in the event this turns out to be an artifact.

I am not familiar with the specifics of DESI-MSI and will defer to those with more expertise. For the record, I remain skeptical that a MS imaging approach can specifically identify a compound whose extreme low abundance cannot be detected by normal LC-MS. Again, the authors' should discuss the limitations of the approach.

> We want to take this opportunity to thank reviewer #2 for carefully evaluating the several versions of this manuscript and for their constructive suggestions. We have now implemented the discussion with a section "Limitations of the study" that it can be found in the new version of this manuscript, including a critical assessment of the LC-MS/MS versus DESI-MSI approaches.

Reviewer #4 (Comments for the Author):

As stated in my earlier review, the authors present very solid results on the role of OPDA-aa conjugates after wounding and necrotroph infection that will clarify somewhat conflicting interpretations in the field.

> We thank the reviewer #4 for their valuable comments and suggestions. The final version of this manuscript benefitted from the critical revision we have received from all the reviewers that have been involved during the revision process.

I had only two recommendations (apart from minor changes in the text), which were unfortunately not considered by the authors

1. GH3.3 localization is not a major finding of this work, but drawing conclusions from OE lines may prove inaccurate in the long term (in agreement with Rev2), particularly if plant selection was not done properly. Shifting the data to the supplement and toning down the localization interpretation was not done in the revised version, but is still advisable. In fact, OE of cytosolic proteins often results in nuclear signals as pointed out by Rev2 as well

> We have now implemented the discussion with a section "Limitations of the study" that it can be found in the new version of this manuscript, including a critical assessment of the subcellular localization study of GH3.3.

2. As GH3.3 OE lines were used to show localization (Fig4), it remains unclear how come the authors did not perform any phenotypic analyses during the 2-month revision time. If the authors

used T1 or segregating T2 lines to draw conclusions for localisation, it only strengthens the concerns raised in point1.

> We thank the reviewer #4 for raising this point. Confocal microscopy was performed on the third generation of at least eight stable transgenic lines derived from different first-generation plants, with a consistent subcellular localization pattern observed across all lines. This information can now be found in the legend of Figure 4E. We want to emphasize that dissecting phenotypic differences might require more efforts when dealing with gene redundancy, as it is the case for the GH3 family. As previously stated in our reply to the reviewer #4 during the first round of revision, our work is a continuous process, and we are currently in the process of phenotyping and profiling the GFP-GH3 OE line. Unfortunately, we could not provide OPDA-aa profiling from the GFP-GH3 OE line within the given time because one of our MS instruments was out of order for a long time during the revision. Nonetheless, we are committed continuing to study OPDA-aa metabolism and localization, and will share our findings in subsequent publications.

04-Nov-2024
Dr. Federica Brunoni
Laborator rustovych regulatoru
Olomouc
Czech Republic

MSID: PP2024-RA-02186DR2
MS TITLE: Homeostasis of cis-OPDA amide conjugates upon plant stress responses

Dear Dr. Federica Brunoni:

I am pleased to inform you that your revised manuscript "Homeostasis of cis-OPDA amide conjugates upon plant stress responses" has been accepted for publication in Plant Physiology. Your manuscript will be checked for consistency with journal formatting and image standards by one of our Science Editors, who will contact you shortly. They may have a number of queries and suggestions for you. Please keep in mind that they work to increase the online visibility of your article and its attraction for readers from the widest of backgrounds. You will have the opportunity to review any revisions, and I hope you will work constructively with them to get the most out of your research. You will also have the opportunity to review all changes when you receive the author proofs for your article.

Once validated for publication, your article will be sent to a Science Editor for a final review of your manuscript and source files. After your final files are uploaded, your article will appear in the next available issue and the online Preview version of your article will be posted after your signed Copyright and Checklist forms and final source files are received.

Please be sure to include the following in your manuscript file:
Harvard style citations
List of all author contributions and funding information at the beginning of manuscript after the titles and author list.
List all supplemental materials associated with the article, with titles, at the end of the ms, before the references and acknowledgments section.
Complete Figure Legends for all figures appearing in the article listed before the Literature Cited.

LICENSE INFORMATION Shortly after your paper is sent to production, you will receive an email from our publisher's author support team, **SciPris**, with information on article processing charges, as well as the types of Open Access license you would be able to purchase. The accepted version of your manuscript will not appear on our Advance Articles page until the license is selected and signed, so please look out for an email from SciPris. For more information see the Article Publication Fees section of our author guidelines: https://academic.oup.com/plcell/pages/General-Instructions

Finally, we encourage your submission of artwork for the journal cover. If there is an image or illustration related to your paper that you would like to have considered, please email it in .pdf or .tif format to clowe@aspb.org. An image title, brief, 2-3 sentence description of the image, and the name of the person credited for the image are also encouraged. If your image is selected to appear on the online cover of the journal, you will be contacted with the scheduled issue date and further information.

Thank you and congratulations on your Plant Physiology paper!

Sincerely,
Editor, Plant Physiology

=====
FIRST AUTHOR PROFILES
=====

We recognize the hard work that goes into being First Author of a Plant Physiology paper by publishing a "First Author Feature" for each First Author alongside your paper.

If the first author(s) would like to submit information for a feature on Facebook and Twitter, fill out the form by clicking on the link below:

Plant Physiology First Author Feature Form: https://forms.office.com/r/ZBCGZfhm8F
If you cannot access the form through the link above, please contact Rachel at rbelsky@aspb.org.

To upload an image of yourself to accompany your profile, please put your photo in the correct journal folder at the link below. You must label your image: Author First Name Last Name Article Title.

First Author Profile Image Dropbox: https://www.dropbox.com/scl/fo/xz029p7qjr2jml2rp2kmq/h?dl=0&rlkey=6fkxcaqmxx35ap152b2osy1fz

=====
IMPORTANT REMINDER: PEER REVIEW REPORTS
=====

If you opted to publish a peer review report along with your article during the original submission process, it will be prepared by the editorial staff and publicly posted with your manuscript, inside the zip file that contains any other supplemental material. As a reminder, the peer review report is a public record of all comments from editors and reviewers, as well as your prior responses, as you received them in the decision letters for each draft of your manuscript. If you agreed to publish this report and have changed your mind, or are not sure if you selected this option, please contact the editorial office as soon as possible before signing the license agreement from our publisher.

-- Reviewer comments:
